# Exploring the Antiglioma Mechanisms of Luteolin Based on Network Pharmacology and Experimental Verification

**DOI:** 10.1155/2021/7765658

**Published:** 2021-11-27

**Authors:** Renxuan Huang, Rui Dong, Nan Wang, Beiwu Lan, Hongyang Zhao, Yufei Gao

**Affiliations:** ^1^Department of Neurosurgery, China-Japan Union Hospital of Jilin University, Changchun, Jilin, China; ^2^Department of Neurology, China-Japan Union Hospital of Jilin University, Changchun, Jilin, China

## Abstract

Luteolin, a natural flavone compound, exists in a variety of fruits and vegetables, and its anticancer effect has been shown in many studies. However, its use in glioma treatment is hampered due to the fact that the underlying mechanism of action has not been fully explored. Therefore, we elucidated the potential antiglioma targets and pathways of luteolin systematically with the help of network pharmacology and molecular docking technology. The druggability of luteolin, including absorption, excretion, distribution, and metabolism, was assessed via the Traditional Chinese Medicine Systems Pharmacology Database and Analysis Platform (TCMSP). The potential targets of luteolin and glioma were extracted from public databases, and the intersecting targets between luteolin and glioma were integrated and visualized by a Venn diagram. In addition, GO and KEGG pathway analysis was engaged in Metascape. The network of the luteolin-target-pathway was visualized by Cytoscape. Ultimately, the interactions between luteolin and predicted key targets were confirmed by Discovery studio software. According to the ADME results, luteolin shows great potential for development into a drug. 4860 glioma-associated targets and 280 targets of luteolin were identified, of which 205 were intersection targets. 6 core targets of luteolin against glioma, including AKT1, JUN, ALB, MAPK3, MAPK1, and TNF, were identified via PPI network analysis of which AKT1, JUN, ALB, MAPK1, and TNF harbor diagnostic value. The biological processes of luteolin are mainly involved in the response to inorganic substances, response to oxidative stress, and apoptotic signaling pathway. The essential pathways of luteolin against glioma involve pathways in cancer, the PI3K-Akt signaling pathway, the TNF signaling pathway, and more. Meanwhile, luteolin's interaction with six core targets was verified by molecular docking simulation and its antiglioma effect was verified by *in vitro* experiments. This study suggests that luteolin has a promising potential for development into a drug and, moreover, it displays preventive effects against glioma by targeting various genes and pathways.

## 1. Introduction

Glioma is the most common and malignant brain tumor, with more than 10,000 cases diagnosed per year and a five-year survival rate of only 5% [1]. The current standard of therapy is surgery coupled with adjuvant radiotherapy and chemotherapy with temozolomide (TMZ) that can be combined with intermediate-frequency alternating electric field therapy, if necessary [2, 3]. However, this treatment does not significantly change survival time, and the median overall survival is still approximately 15 months [4]. Moreover, individually targeted therapies often fail because glioma cells show remarkable heterogeneity, both between and within tumors [5]. Therefore, it is extremely important to investigate new effective drugs for glioma treatment.

**Figure 1:**
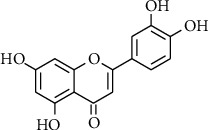
Chemical structure of luteolin (downloaded from the PubChem database: CID: 5280445).

Natural products have shown a wide range of pharmacological or biological activities, rendering them potential candidates of treatment strategies against central nervous system diseases such as neurological disorders, neurodegenerative diseases, and tumors [6, 7]. It has been observed previously that traditional Chinese medicines (TCMs) are effective in the treatment of glioma, which has aroused the attention of researchers worldwide [8]. One of the active ingredients in many types of TCMs is 3′,4′,5,7-tetrahydroxyflavone, also known as luteolin (Figure 1) [9, 10]. Luteolin shows various pharmacological properties, including neuroprotective, cardioprotective, anti-inflammatory, antidiabetic, antimicrobial, antioxidant, and pro-oxidant effects, as the previous studies verified [11–13]. Importantly, its ability to cross the blood-brain barrier makes it a potential therapeutic drug for central nervous system diseases including glioma [14, 15]. Interestingly, an antiglioma effect of luteolin has previously been demonstrated. Chakrabarti and colleagues showed that luteolin inhibited glioma cell migration and invasion and blocked angiogenic and survival pathways [16]. Wang and colleagues demonstrated that luteolin induced apoptosis of glioma through inducing ER stress and mitochondrial dysfunction [17]. However, its potential mechanism of action on glioma cells remains unclear and needs further elucidation.

Network pharmacology can help to explore the systemic effects of TCMs by combining the methods of biology, pharmacology, and bioinformatics. These analyses provide the potential biological processes and pathways by which TCMs may work. Molecular docking is an emerging technology for drug design that can simulate the interaction among small molecules and their receptors. This technique has been widely used in pharmacological testing studies, thus providing a basis for drug design [18]. In addition, molecular dynamics simulation was designed to validate molecular docking and to investigate the dynamic motions and conformational changes that occur in the docked structure [19]. These technologies help us to access the potential mechanisms of action of TCMs in treatment and ultimately develop new drugs. This study aims to explore the pharmaceutical potential of luteolin and systematically assesses the therapeutic targets and mechanisms of luteolin in glioma via network pharmacology and the molecular docking technique.

## 2. Materials and Methods

### 2.1. Pharmacological Properties of Luteolin

Traditional Chinese Medicine Systems Pharmacology Database and Analysis Platform (TCMSP: https://tcmspw.com/tcmsp.php) is a unique systems pharmacology platform designed for TCMs. It provides information on the pharmacological properties of natural drugs including drug-likeness (DL), oral bioavailability (OB), intestinal epithelial permeability (Caco-2), blood-brain barrier (BBB), and more [20]. In this study, by employing the TCMSP database with the search term “luteolin,” we investigated its pharmacokinetic properties.

### 2.2. Collection of Luteolin and Glioma-Related Target Genes

Luteolin-related targets were collected via PharmMapper using default values (http://www.lilab-ecust.cn/pharmmapper/submitfile.html) [21], SwissTarget Prediction (https://www.swisstargetprediction.ch) [22], and the Comparative Toxicogenomics Database (CTD; http://ctdbase.org/) [23]. The mol file of luteolin was obtained from PubChem (https://pubchem.ncbi.nlm.nih.gov/). Glioma-related targets were identified via several public databases including Online Mendelian Inheritance in Man (OMIM, https://omim.org/), Therapeutic Target Database 2020 (TTD) (http://db.idrblab.net/ttd/) [24], GeneCards (https://www.genecards.org/), and DisGeNET (v7.0) (http://www.disgenet.org/). All corresponding target gene symbols were derived from the UniProt database (http://www.uniprot.org/) [25].

### 2.3. Protein-Protein Interaction (PPI) Network Construction and Core Target Analysis of Luteolin-Glioma Interaction

The intersecting targets of luteolin and glioma represent the glioma targets of luteolin and were visualized with a web tool (*Draw Venn Diagram*, http://bioinformatics.psb.ugent.be/webtools/Venn/). Based on these common targets, a PPI network was constructed via importing the luteolin against glioma target genes to STRING (11.0) database (https://string-db.org/) using a medium confidence of 0.4 [26]. This PPI network was visualized by the Cytoscape software (version 3.8.0) [27]. Hub targets of this PPI network were mined with Cytohubba and MCODE, and targets were screened from the top 10 nodes of degree for maximum neighborhood component (MNC) and maximal clique centrality (MCC). The hub targets were screened with MCODE using default settings. Finally, the overlapping targets of Cytohubba and MCODE, representing the hub targets, were visualized via *Draw Venn Diagram*.

### 2.4. GO and KEGG Pathway Enrichment Analysis

The Gene Ontology (GO) and Kyoto Encyclopedia of Genes and Genomes (KEGG) enrichment of glioma were performed using the Funrich software (3.1.3) and the clusterProfiler package R (v3.14.3). The GO and KEGG enrichment analysis of luteolin against glioma was conducted using the functional annotation tool of Metascape (https://metascape.org) and “*P* Value Cutoff” as well as “Min Overlap” was set to 0.01 and 3, respectively. GO functionally annotates key targets as cellular components (CCs), molecular functions (MFs), and biological processes (BPs). KEGG enrichment analysis reveals the potential biological process of hub targets. Eventually, the bubble chart of the KEGG enrichment analysis was displayed using the bioinformatics platform (http://www.bioinformatics.com.cn/).

### 2.5. Glioma and Luteolin-Related Target Pathway Map Analysis

The intersection targets of glioma and luteolin were uploaded to KEGG Mapper, an analysis tool of the KEGG website (https://www.kegg.jp/) to provide a visualization of the possible complex interactions of luteolin in the glioma setting.

### 2.6. Analysis of Diagnostic Value

RNAseq data of glioma tissues (GBMLGG), extracted from TCGA, and corresponding normal tissue data originating from GTEx, were downloaded via UCSC XENA (https://xenabrowser.net/datapages/). Then, the diagnostic value of six core targets was assessed by employing pROC and ggplot2 R software packages.

### 2.7. Molecular Docking

Molecular docking has been proven to be an efficient tool for the discovery of novel drugs and the identification of small molecules that bind to target proteins based on structural molecular biology data [28, 29]. A total of six core targets were included in the molecular docking simulation, including AKT1, JUN, ALB, MAPK3, MAPK1, and TNF. The molecular structures of these targets were obtained from the Protein Data Bank (PDB). The 3D structure of luteolin was dealt with using ChemOffice software and was prepared using the “Prepare Ligands” command to generate an effective three-dimensional conformation by Discovery Studio software (version 4.5.0, BIOVIA, USA). The original small molecule binding site was defined as the active site by the “Define and Edit Binding Site” command. The target proteins were prepared by using the “Prepare Protein” command to remove polyconformations and replenish missing amino acid residues. Finally, the “CDOCKER” module, which is based on the CHARMm-algorithm, was executed for simulation of molecular docking. Docking score and binding energy (kcal/mol) were evaluated to assess the affinity of luteolin to target proteins.

### 2.8. Molecular Dynamics Simulation

The general AMBER force field was used to model luteolin, while the ff14SB force field was used to model proteins [30]. Hydrogen atoms were added using the tLEAP module of the Amber Tools program. Initial parameterization of luteolin was carried out using the Gaussian 09 software. All the molecular dynamics simulations were performed with the Sander and Pmemd modules of the AMBER14 software [31]. Each complex was placed in a TIP3P water box after the addition of antechamber ions to maintain the system electrically neutral, leading to a minimum of 10.0 Å between the solvent and the nearest box edge. In the MD simulation process, nonbonded interactions were calculated utilizing the Particle-Mesh Ewald method with a cutoff of 10 Å [32], and the SHAKE algorithm was used to constrain all bonds involving hydrogen atoms with a 2 fs timestep [33]. Under constant volume conditions, the system was heated from 0 K to 300 K within 60 ps, followed by equilibration of the solvent density at a constant pressure system (*T* = 300 K, *P* = 1 atm) and finally, sampling for 100 ns at constant pressure, saving one frame (conformation) per ps for subsequent analysis.

### 2.9. Validation of the Luteolin Effect on Glioma Cells

U87MG [34] and SHG44 [35] cell lines (from Shanghai Zishi Biotech Co., Ltd.) were cultured in Dulbecco's modified Eagle medium (DMEM) with 10% fetal bovine serum (Invitrogen, USA) at 37°C with 5% CO_2_. Luteolin was purchased from MedChemExpress (CAS No.:491-70-3). The cells were plated in 96-well plates (8000/well) for an MTT assay and in 6-well plates (3 × 10^5^/well) for western blot (WB) and semiquantitative real-time-PCR (sqRT-PCR) analysis. After incubation with luteolin for 24 h, 10 *μ*l MTT solution (5 mg/mL) was added to the 96-well plates for a 4 h incubation period and absorbance values at 490 nm were measured. The measured values were expressed as a ratio by comparing them to the control group values. More details for WB and sqRT-PCR were described previously [36]. In brief, cells were lysed in radio immunoprecipitation assay (RIPA) lysis buffer (P0013B, Beyotime). Clear lysates were harvested and boiled with loading buffer (20315ES05, Yeasen) for 10 min, separated by SDS-PAGE, and afterwards transferred to PVDF membranes. The membranes were incubated with primary antibodies at 4°C overnight and with HRP-conjugated secondary antibodies the following day. Finally, a Syngene Bio Imaging instrument (Synoptics, Cambridge) was used for chemiluminescence detection and visualization. The total RNA was extracted according to the protocol of the Axygen® AxyPrep Multisource RNA Midiprep Kit (AP-MD-MS-RNA-25, Axygen). The reverse transcription reaction was performed by the Hifair® II 1st Strand cDNA Synthesis Kit (11119ES60, Yeasen), and the sqRT-PCR was performed on a Bio-rad CFX-96 real-time PCR system using Hieff® qPCR SYBR® Green Master Mix (11201ES03, Yeasen). mRNA levels were normalized to those of ACTB. The primer sequences are *AKT1*: 5′-AGCGACGTGGCTATTGTGAAG-3′ (forward) and 5′-GCCATCATTCTTGAGGAGGAAGT-3′ (reverse); *ALB*: 5′-TGCAACTCTTCGTGAAACCTATG-3′ (forward) and 5′-TGCAACTCTTCGTGAAACCTATG-3′ (reverse); *JUN*: 5′- TCCAAGTGCCGAAAAAGGAAG-3′ (forward) and 5′-CGAGTTCTGAGCTTTCAAGGT-3′ (reverse); *TNF*: 5′-GAGGCCAAGCCCTGGTATG-3′ (forward) and 5′- CGGGCCGATTGATCTCAGC-3′ (reverse); *JUN*: 5′- TCCAAGTGCCGAAAAAGGAAG-3′ (forward) and 5′-CGAGTTCTGAGCTTTCAAGGT-3′ (reverse); *MAPK1*: 5′- TACACCAACCTCTCGTACATCG-3′ (forward) and 5′-CATGTCTGAAGCGCAGTAAGATT-3′ (reverse); and *MAPK3*: 5′-ATGTCATCGGCATCCGAGAC-3′ (forward) and 5′- GGATCTGGTAGAGGAAGTAGCA-3′ (reverse). ACTB (B661102) was purchased from Sangon Biotech (Shanghai) Co., Ltd., and the antibodies were purchased from Santa Cruz Biotechnology (Shanghai) Co., Ltd.

### 2.10. Statistical Analysis

Data are expressed as the mean ± SD. Statistical analysis was performed by Student's *t*-test using GraphPad Prism 8 (La Jolla, CA, USA), and *P* < 0.05 was considered as being statistically significantly different. All experiments were performed at least in triplicate.

## 3. Results

### 3.1. Pharmacological Properties of Luteolin

Luteolin was studied via TCMSP to analyze its ADME characteristics (Table 1). Luteolin shows drug development potential because it complied with DL ≥ 0.18, OB ≥ 30%, and “Lipinski's Rule of Five” (including MW, AlogP, TPSA, Hdon, and Hacc).

### 3.2. Identification of Luteolin- and Glioma-Related Targets

**Figure 2:**
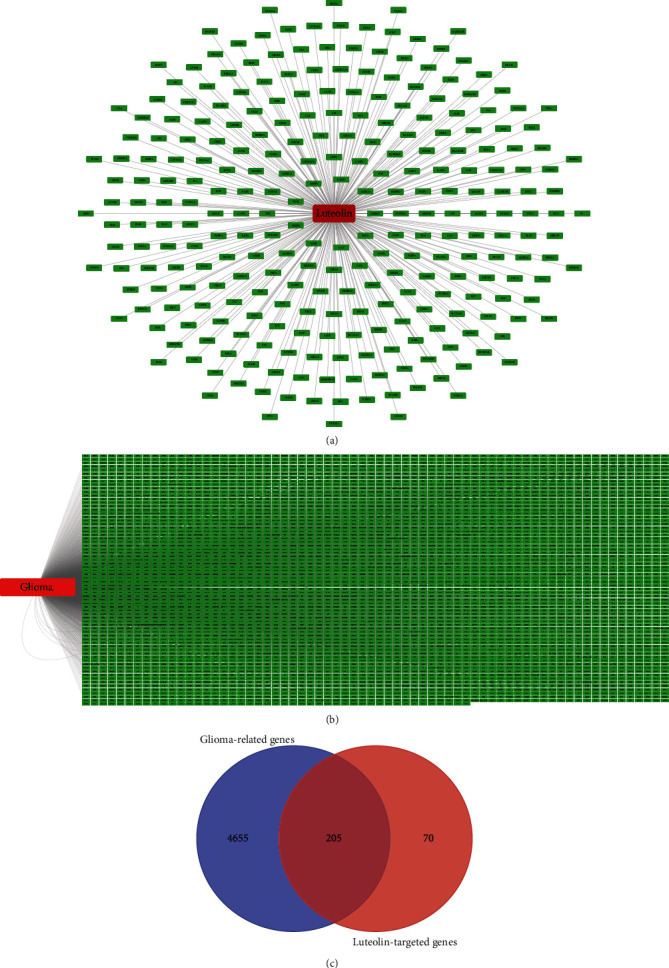
Common targets of luteolin and glioma.

To reveal the potential targets of luteolin in glioma cells, all 275 targets of luteolin were identified with the help of Swiss Target Prediction (111), CTD (124), and PharmMapper (71) as shown in Figure 2(a). In addition, there remained 4860 glioma-associated targets (Figure 2(b)), among which 4917 genes were identified with the help of GeneCards, 3097 of DisGeNET, 17 of TTD, and 9 of OMIM. As a result, a total of 205 intersection targets were screened for the following study of luteolin targets in glioma cells (Figure 2(c)). All of these targets are shown in the supplementary material section (Tables S1 and S2).

### 3.3. GO and KEGG Pathway Enrichment Analysis

**Figure 3:**
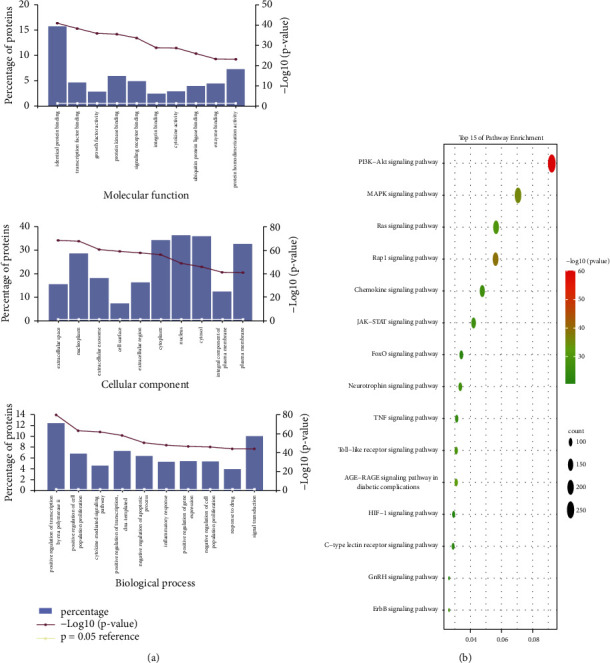
GO and KEGG pathway enrichment analysis for glioma.

First, we performed a GO analysis of glioma-related targets through Funrich (3.1.3) to reveal the potentially involved therapeutic pathways. 10 significantly enriched BPs, CCs, and MFs terms are shown in Figure 3(a) (*P* < 0.05). The results suggest that the glioma-related targets could act either through identical protein binding, transcription factor binding, extracellular space, nucleoplasm, positive regulation of transcription by RNA polymerase II, or positive regulation of cell population proliferation. For the KEGG enrichment analysis, we used the clusterProfiler R package (v3.14.3). The results of the KEGG analysis included 56 signaling pathways (*P* < 0.05) (Table S3). The top 15 markedly enriched signaling pathways of glioma are shown in Figure 3(b), in which the “PI3K-Akt signaling pathway” comprised 281 counts (the largest number of involved targets) and the lowest *P* value (9.99E-61).

**Figure 4:**
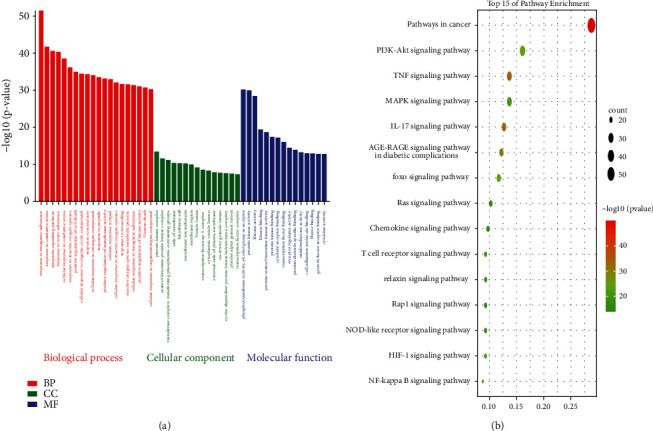
GO and KEGG pathway enrichment analysis for the targets of luteolin in glioma cells.

To shed light on the potential biological function of luteolin in glioma cells, 205 intersection targets were submitted to Metascape to analyze their biological function. 15 significantly enriched BP, CC, and MF terms are shown in Figure 4(a) (*P* < 0.01) with regard to inorganic substance GO:0010035, protein kinase complex GO:1902911, phosphotransferase activity, and an alcohol group as acceptor GO:0016773 as the top ones, respectively. The result of GO enrichment analysis is summarized in Table S4. The analysis of the KEGG pathway analysis revealed that 205 common targets of luteolin and glioma were enriched in 183 pathways (*P* < 0.05), including 49 signaling pathways (Table S5). The KEGG enrichment analysis revealed the pathway by which luteolin exerts its therapeutic effect on glioma cells. Figure 4(b) shows the top 15 markedly enriched signaling pathways of luteolin in glioma cells, in which the “pathways in cancer” feature comprised 59 counts (the largest number of involved targets) and the lowest *P* value (1.55E-50).

### 3.4. Glioma- and Luteolin-Related Target Pathway Map Analysis

**Figure 5:**
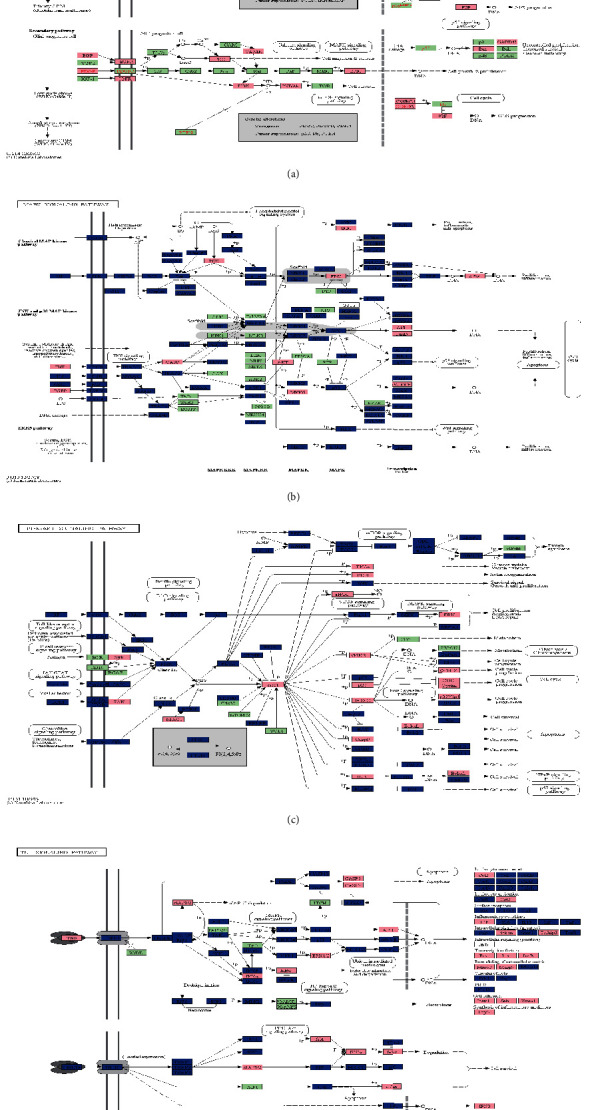
The integrated pathways map of luteolin in glioma setting.

The glioma pathway map and the signaling pathways of the luteolin-target-pathway network were combined into an integrated pathway map to provide a visualization of the complex interactions of luteolin in the glioma setting (Figure 5). As shown in Figure 5(a), glioma disease pathways comprise a calcium signaling pathway and a MAPK pathway involved in cell migration and mitosis as well as the mTOR signaling pathway involved in cell survival. Another pathway related to cell growth and proliferation is the ErbB signaling and the cytokine-cytokine receptor interaction pathway. The three most important luteolin-related signaling pathways in the glioma setting include the hsa04010: MAPK-signaling pathway (Figure 5(b)), the hsa04151: PI3K-Akt signaling pathway (Figure 5(c)), and the hsa04668: TNF signaling pathway (Figure 5(d)). According to this map, AKT, ERK, and TNF are involved in three signaling pathways and play a pro-proliferation role in glioma. However, luteolin may inhibit glioma through these targets as well.

### 3.5. Luteolin-Target-Pathway Network

**Figure 6:**
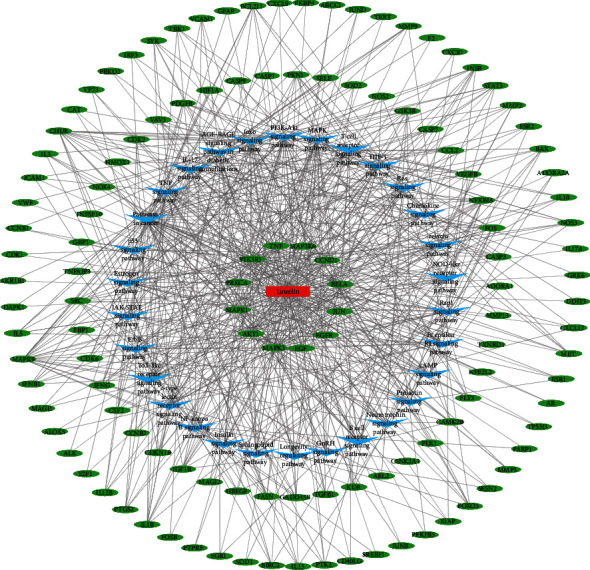
Luteolin-target-pathway network.

To clarify the mechanism of luteolin in the treatment of glioma through multiple pathways and targets, the luteolin-target-pathway network was visualized by Cytoscape 3.8.0 in Figure 6, which included 155 nodes (124 targets and 30 pathways) and 719 edges. These signaling pathways and enriched targets may be the critical antiglioma mechanisms of luteolin, including the PI3K-Akt-, the TNF-, the p53-, the HIF-1-, the MAPK-, the NF-kappa B-, the JAK-STAT-, the Ras-signaling pathway and others.

### 3.6. PPI Network Analysis of Luteolin in the Glioma Setting

**Figure 7:**
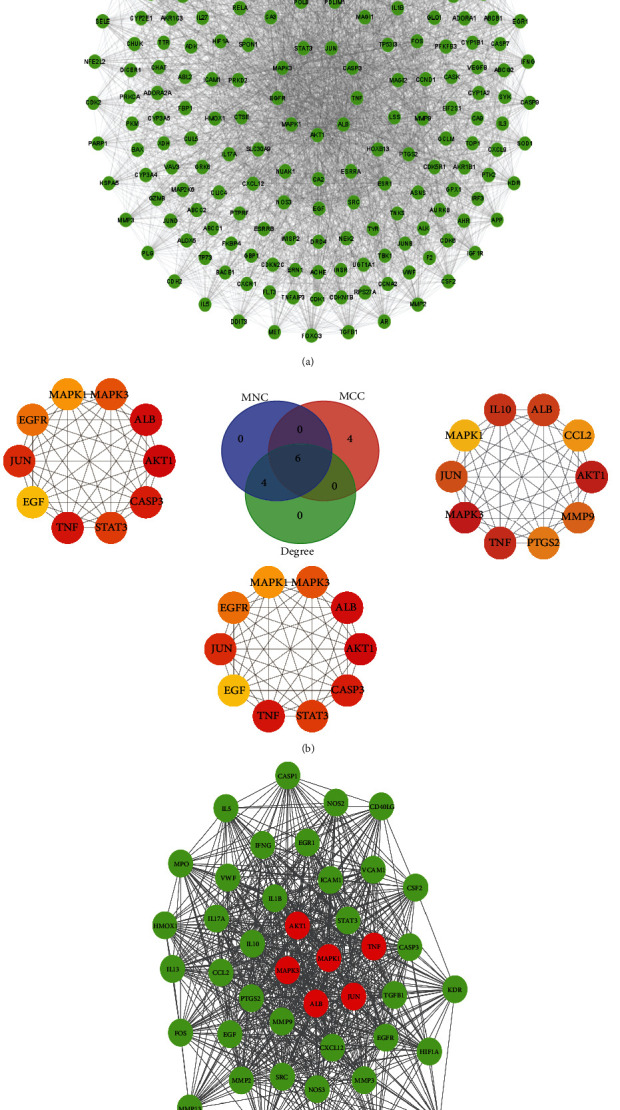
PPI network of luteolin with the hub targets in a glioma setting was screened by CytoHubba and MCODE.

The STRING database was used to further reveal the functional mechanisms of luteolin in glioma. The PPI network of luteolin in the glioma setting had 203 nodes and 3225 edges, in which nodes and edges meant targets and their interactions, respectively. An initial PPI network was established by Cytoscape 3.8.0 (Figure 7(a)). Six hub targets were obtained from CytoHubba analysis including AKT1, JUN, ALB, MAPK3, MAPK1, and TNF (Figure 7(b)). The MCODE analysis showed the most significant modules with scores of 35.143 (Figure 7(c)) which contained 43 nodes with 738 edges. This module comprised six hub targets in accordance with the results obtained from the CytoHubba analysis.

### 3.7. Diagnostic Value of Hub Targets

**Figure 8:**
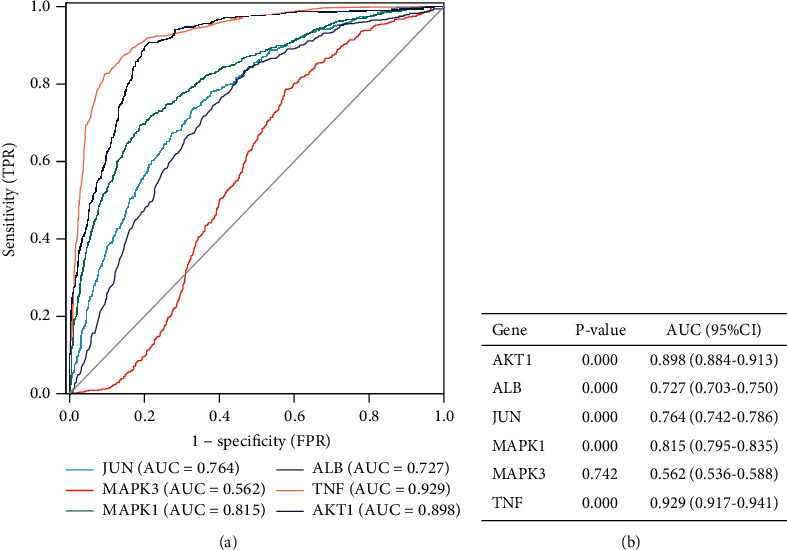
The diagnostic value of hub targets.

We analyzed the diagnostic value of the six core targets through constructing ROC curves and their corresponding area under the curve (AUC) from TCGA and GTEx datasets.  Figure 8 shows that the AUC for AKT1, ALB, JUN, MAPK1, MAPK3, and TNF in glioma patients and normal controls was 0.898 (95% confidence interval (CI), 0.884–0.913; *P* < 0.05), 0.727 (95% CI, 0.703–0.750; *P* < 0.05), 0.764 (95% CI, 0.742–0.786; *P* < 0.05), 0.815 (95% CI, 0.795–0.835; *P* < 0.05), 0.562 (95% CI, 0.536–0.588; *P* > 0.05), and 0.929 (95% CI, 0.917–0.941; *P* < 0.05).

### 3.8. Validation Analysis of Luteolin Effects on Glioma by Molecular Docking and Molecular Dynamics Simulation

**Figure 9:**
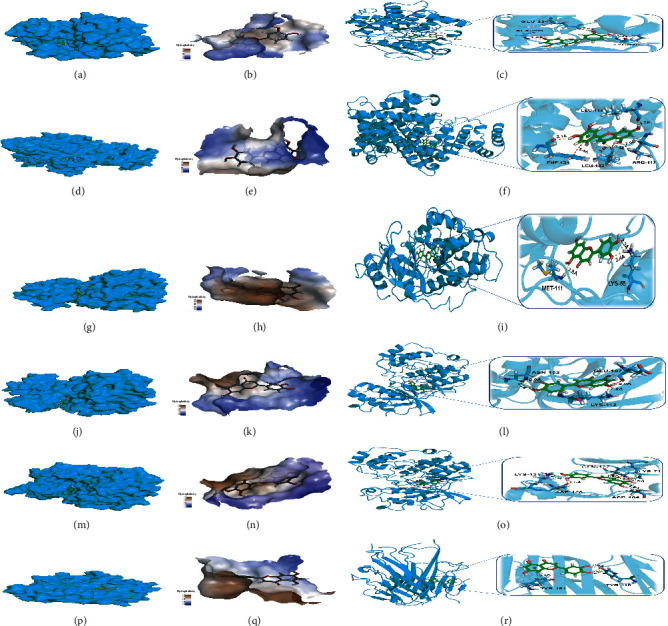
The molecular docking between luteolin and core target proteins.

Based on the PPI network, six core targets were selected for molecular docking (Figure 9). The binding affinities of the target proteins and luteolin indicated as docking scores and binding energy were shown by comparison to the original ligand of the target proteins. These docking scores and binding energies are shown in Table 2. It indicates that luteolin has a stronger or similar effect to prototype ligands, such as AKT1, ALB, JUN, MAPK1, MAPK3, and TNF.  Table 3 shows the specific docking bonds of luteolin to six targets.

**Figure 10:**
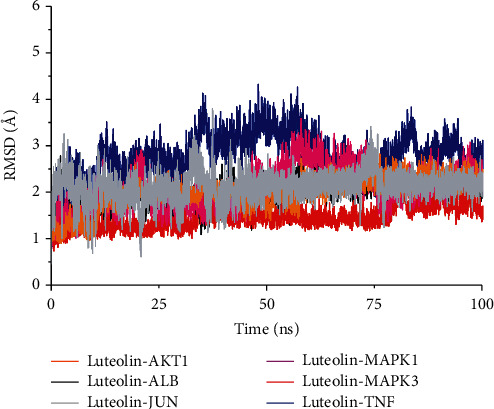
The molecular dynamics simulation between luteolin and the core target proteins.

To analyze the fluctuation of the interfaces of luteolin-protein complexes, each complex was simulated for 100 ns. The results of the molecular dynamics simulations of luteolin with the six core proteins are shown in Figure 10 and demonstrate that the root-mean-square deviation (RMSD) of the luteolin-protein complex was stable. The fluctuations in RMSD values of luteolin-MAPK3, luteolin-JUN, luteolin-AKT1, and luteolin-ALB were smaller than those of luteolin-TNF and luteolin-MAPK1, thus indicating luteolin shows a higher binding affinity to MAPK3, JUN, AKT1, and ALB.

### 3.9. Effect of Luteolin on Glioma Cells

**Figure 11:**
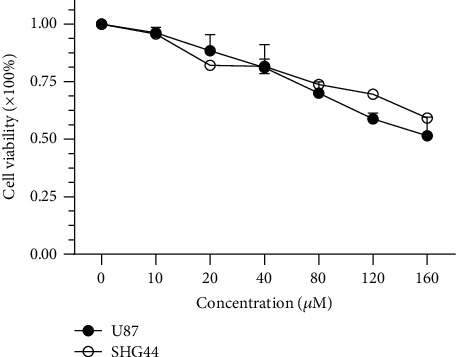
Effect of luteolin on the viability of glioma cells.

To validate the effect of luteolin on glioma cells, the classical glioma cell lines U87 and SHG44 were selected to perform cell-based experiments. The results of the MTT assay showed that the viability of the two cell lines decreased compared to control cells when the luteolin concentration was increased (Figure 11(a)). Cell viabilities of U87/SHG44 treated for 24 h with 10, 20, 40, 80, 120, and 160 *μ*M luteolin were 96.25%/95.65%, 88.45%/82.04%, 81.11%/81.66%, 69.95%/73.78%, 58.81%/69.52%, and 51.46%/59.14%, respectively. Thus, luteolin can effectively decrease the viability of glioma cells in a concentration-dependent manner.

**Figure 12:**
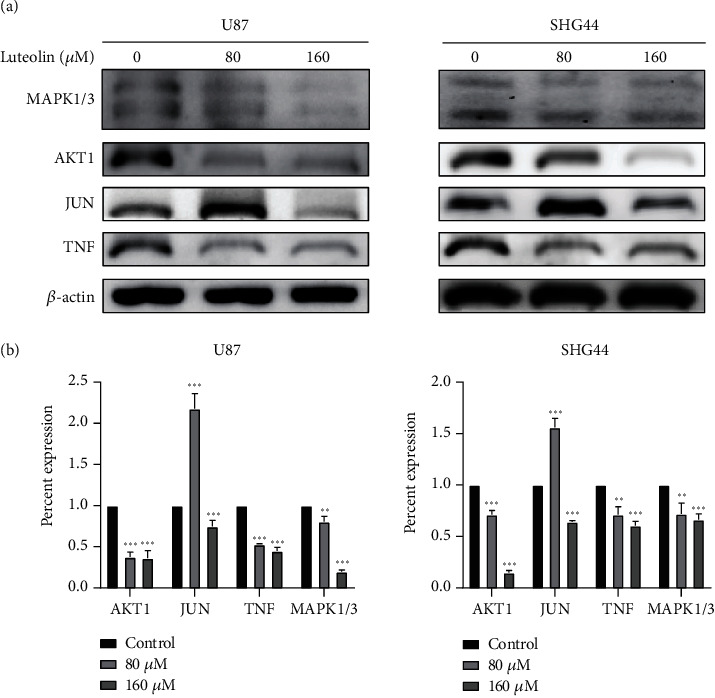
The expression of six core target proteins was determined by western blotting.

**Figure 13:**
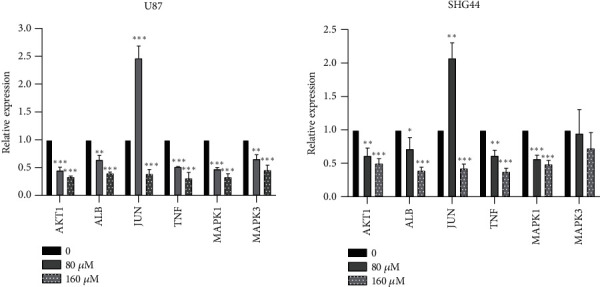
The expression of six core target mRNAs was determined by sqReal-Time PCR.

Western blotting and sqReal-Time PCR were used to verify the results of the molecular docking and molecular dynamics simulations of luteolin and its targets. After treatment with luteolin for 24 h, a significantly decreased expression of AKT1, TNF, and MAPK1 in the two cell lines was observed when compared to the control group, with JUN decreasing only after 160 *μ*M incubation (Figure 12). We did not detect ALB protein expression in these cell lines. The sqReal-Time PCR results showed that expression of AKT1, ALB, TNF, and MAPK1 decreased when compared to the control group and JUN decreased only after 160 *μ*M incubation. Moreover, only U87 cells displayed a significantly decreased expression of MAPK3 (Figure 13).

## 4. Discussion

Although the study of glioma treatment has come a long way, glioma still has a very high morbidity and mortality rate [37]. This is due to the fact that, at present, the drugs used for glioma treatment are clinically less effective than necessary [38, 39]. A number of studies on the anticancer effect of luteolin have been performed, and it has been revealed that luteolin has a remarkable antiglioma effect in various cellular and animal models [16, 17, 40]. However, there is still uncertainty about the detailed and molecular mechanisms of luteolin action in glioma cells. This study aimed to identify possible luteolin's targets and pathways through network pharmacology, molecular docking, molecular dynamics simulation methods, and experimental verification.

Potential poor pharmacokinetics and toxicity impede drug discovery and development. Therefore, assessment of the ADME properties should be the first step in the drug discovery process, leading to a significantly accelerated drug discovery process and at the same time reducing side effects and costs [41]. The two most vital metrics for ADME properties estimation of compounds are DL and OB [42]. DL is designed to evaluate whether a compound is chemically suitable to develop into a drug. OB represents a measure of a drug's oral dose that is taken up by the blood and ultimately produces a pharmacological effect and, therefore, reflects the effectiveness of oral drugs for most oral TCMs. For natural plant-derived drug development, a standard of OB ≥ 30% and of DL ≥ 0.18 is required [43]. Moreover, Lipinski's rule of five, comprising molecular weights (MWs) < 500 Daltons and the lipid-water partition coefficient (ALogP) < 5, as well as the numbers of hydrogen-bond donors (Hdon) < 5 and the number of bond acceptors (Hacc)＜10, can identify some very essential drug properties. A compound that complies with this rule of five and disposes of the required pharmacokinetic properties as well as bioavailability is generally considered a more potent drug candidate [44]. The pharmacokinetic properties of luteolin meet these requirements, as shown in Table 1. Another important parameter that should be considered in glioma treatment is the “BBB” potency, which represents the capability of compounds to enter the central nervous system. The compounds with BBB < -0.3 are considered nonpenetrating. However, although luteolin shows lower scores in BBB, some studies confirmed that luteolin could cross the blood-brain barrier effectively [15, 45]. The above data indicate that luteolin has a great potential for drug development.

In previous studies, luteolin has been utilized in the treatment of multiple cancer cell lines through multiple biological effects such as induction of apoptosis, cell cycle arrest, and inhibition of metastasis and angiogenesis [40]. These results are consistent with our MTT assay result, which showed that luteolin could effectively decrease glioma viability. In this context, the results showed that luteolin may regulate the response to inorganic substances, to oxidative stress, the apoptotic signaling pathway, the protein kinase complex, and a series of kinase activities to exert its therapeutic effects on glioma disease (Figure 4). KEGG pathway analysis indicated that the 49 key signaling pathways may be the antiglioma mechanisms of luteolin that include pathways in cancer, PI3K-Akt-, TNF-, and MAPK-signaling pathways. (Figure 4). By target harvesting, six core genes including AKT1, JUN, ALB, MAPK3, MAPK1, and TNF were screened from a total of 205 targets of luteolin against the glioma network. To better understand the possible interaction modes of the core genes, molecular docking and molecular dynamics simulation were performed. The results of the molecular docking analysis showed that luteolin has a stronger or similar binding affinity to prototype ligands than to the six core targets. Moreover, our molecular dynamics simulation showed that these six luteolin-protein complexes were stable. Finally, western blotting and sqRT-PCR were used to verify the predicted targets for luteolin in glioma. The results of Western blotting and sqRT-PCR showed that luteolin was able to inhibit AKT1, MAPK1, and TNF expression in a concentration-dependent manner and JUN was inhibited only at a concentration of 160 *μ*M. We could not detect ALB protein in western blots, but the results of sqRT-PCR showed that luteolin was able to inhibit its expression. Taken together, these outcomes suggest that the six core genes maybe potential targets of luteolin and may play an important role in the future treatment of glioma.

Surprisingly, ALB was described as a potential novel protein marker for glioma [46, 47]. It is worth noting that ALB is a protein with a high abundance and a long half-life in blood [48], and ALB was considered as a promising carrier for oncology treatment-related drugs because of several essential properties. It is a natural carrier of native ligands and other hydrophobic cargo and is able to cross the vascular endothelium. More importantly, it is more preferentially internalized and metabolized by rapidly growing, nutrient-deficient cancer cells [49]. Its cancer-targeting properties, remarkable half-life, and natural ligand-binding properties make it a prospective and already translatable vehicle for luteolin delivery. AKT1 is one of three highly related serine/threonine-protein kinases that regulate many processes involving metabolism, proliferation, DNA repair, cell cycle, and cell survival, contributing to glioma progression, aggressiveness, and resistance to treatment [50]. Consequently, the combination of AKT inhibitors is a promising strategy for glioma treatment [50]. MAPK1 and MAPK3 are the two MAPKs/ERKs that play an essential role in the MAPK/ERK cascade, contributing to the signaling cascade that regulates various cellular processes involving proliferation, differentiation, transcription regulation, and development [51]. The majority of glioblastomas exhibit activation of the extended RAS-MAPK and PI3K-AKT signaling pathways which were considered to be common oncogenic alterations [52]. Luteolin induces apoptosis in glioblastoma cell lines through inhibiting the AKT- and MAPK-signaling pathways and thus contributes to a beneficial treatment [53]. In addition, the PTEN status of gliomas has recently emerged as a major predictor for the success of therapies that are targeting receptor tyrosine kinases [54]. TNF is a major regulator of inflammation that is overexpressed and mainly secreted by macrophages in the tumor microenvironment [55]. However, TNF is also released by gliomas and plays an essential role in promoting tumorigenesis, proliferation, metastasis, and inhibiting apoptosis through the NF-*κ*B signaling pathway [56–58]. JUN is one of the basic leucine zipper-containing dimeric transcription factors that recognizes and binds to the enhancer heptamer motif 5′-TGA[CG]TCA-3' [59]. It regulates wide-ranging cellular processes, including cell proliferation, death, survival, and differentiation through forming stable heterodimers with Fos, Maf, and ATF multigene families, known as activator protein-1 (AP-1) [60]. AP-1 is constitutively activated in glioma and plays an important role in cell proliferation [61]. Small AP-1 activity inhibitory molecules have already been developed and tested with positive effects as a promising target for cancer prevention and therapy [62]. These results closely coincide with our predictive biological processes and signaling pathways involved in antiglioma from GO and KEGG analyses and the results of the pathway map analysis. The above results suggest that AKT1, JUN, ALB, MAPK3, MAPK1, and TNF may be essential targets of luteolin in glioma disease. Besides, AKT1, JUN, ALB, MAPK1, and TNF display good diagnostic value in glioma. More importantly, AKT1, JUN, MAPKs, and TNF contribute to resistance to chemotherapy and radiotherapy, one of the greatest barriers in the treatment of glioma [63, 64]. These results imply that luteolin not only is a multitarget drug but also increases therapy combination efficacy synergistically with other therapeutic strategies to overcome drug resistance. This makes luteolin a potential drug candidate for application in the clinic.

## 5. Conclusions

Taken together, we verified that luteolin is a prospective drug for the development of efficient multitargeted antiglioma TCMs with pharmacology, molecular docking technology, molecular dynamics simulation, and experimental verification. This study provides novel insights into luteolin in future clinical translational research. However, the database used in network pharmacology may omit some potential targets for difficulties in data acquisition. Next, we will validate the further antiglioma potency and mechanisms of luteolin *in vitro* and *in vivo*.

## Figures and Tables

**Table 1:** Pharmacological properties of luteolin.

Name	MW	AlogP	Hdon	Hacc	OB (%)	Caco-2	BBB	DL	FASA-	TPSA	RBN	HL
Luteolin	286.25	2.07	4	6	36.16	0.19	−0.84	0.25	0.39	111.13	1	15.94

**Table 2:** The docking scores and binding energy of 6 hub targets and luteolin.

Protein	PDB ID	Docking scores		Binding energy (kcal/mol)	
		Ligands	Luteolin	Ligands	Luteolin
AKT1	3OCB	−7.45	−6.91	−65.454	−60.811
ALB	4LA0	−6.62	−7.53	−56.512	−71.015
JUN	2GMX	−7.48	−7.94	−59.486	−61.932
MAPK1	3QYW	−6.59	−5.87	−64.588	−57.007
MAPK3	4QTB	−7.26	−7.78	−68.534	−75.113
TNF	5MU8	−7.12	−5.67	−57.624	−41.571

**Table 3:** Hydrogen-bond interaction parameters for luteolin with target protein residues.

Protein	Protein residues	Bond	Distances (Å)
AKT1	LYS-158	H-bond	2.4
	ALA-230	H-bond	2.0
	GLU-234	H-bond	1.8

ALB	LEU-115	H-bond	1.7
	ARG-117	H-bond	2.0
	LEU-182	H-bond	3.4
		H-bond	2.4
	PHE-134	H-bond	2.1

JUN	LYS-55	H-bond	2.3
		H-bond	2.4
	MET-111	H-bond	2.8

MAPK1	GLU-107	H-bond	2.0
	LYS-112	H-bond	2.0
	ASN-152	H-bond	2.0
		H-bond	2.2

MAPK3	LYS-71	H-bond	2.6
		H-bond	2.6
	GLN-122	H-bond	1.9
	ASP-128	H-bond	1.6
	LYS-131	H-bond	2.7
	ASP-184	H-bond	1.8

TNF	TYR-119	H-bond	2.0
		H-bond	2.2

## Data Availability

The data are included within the supplementary information files.

## Additional Points

The graphical abstract is the workflow of targets prediction and verification.

## Supplementary Materials

Supplementary Materials The Supplementary Materials to this article include the following: the prediction targets of luteolin, the prediction targets of glioma, the signaling pathways in KEGG enrichment analysis of glioma-related targets, the result of GO enrichment analysis, and the 49 signaling pathways in KEGG pathway analysis for the targets of luteolin in glioma cells.
