# Supplementary material for: Exploring the Antiglioma Mechanisms of Luteolin Based on Network Pharmacology and Experimental Verification
Source: Evid Based Complement Alternat Med. 2021 Nov 27;2021:7765658. doi: 10.1155/2021/7765658 (PMC8643232; doi:10.1155/2021/7765658)
Supplement: Supplementary Materials — The Supplementary Materials to this article include the following: the prediction targets of luteolin, the prediction targets of glioma, the signaling pathways in KEGG enrichment analysis of glioma-related targets, the result of GO enrichment analysis, and the 49 signaling pathways in KEGG pathway analysis for the targets of luteolin in glioma cells. [file 7765658.f1.zip › 7765658.f1/Graphical abstract.pdf]

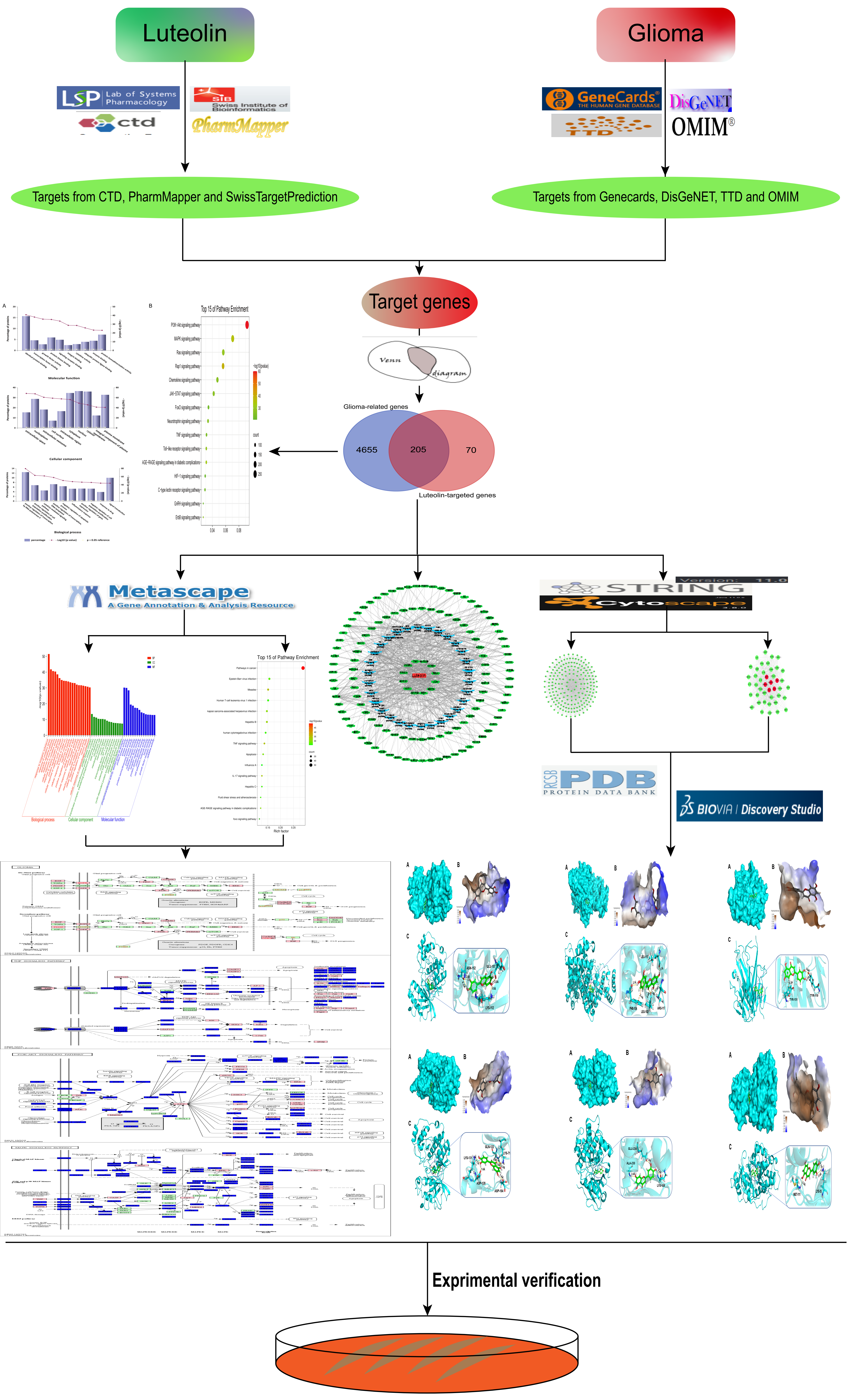
Luteolin
Glioma
Lab of Systems Pharmacology
ctd
Swiss Institute of Bioinformatics
PharmMapper
GeneCards
THE HUMAN GENE DATABASE
DisGeNET
OMIM®
TTD
Targets from CTD, PharmMapper and SwissTargetPrediction
Targets from Genecards, DisGeNET, TTD and OMIM
Target genes
Venn diagram
Glioma-related genes
4655
205
70
Luteolin-targeted genes
Top 15 of Pathway Enrichment
Molecular function
Cellular component
Biological process
Metascape
A Gene Annotation & Analysis Resource
STRING
Cytoscape
Biological process
Cellular component
Molecular function
Rich factor
PDB
PROTEIN DATA BANK
BIOVIA | Discovery Studio
Exprimental verification
