# Supplementary material for: Exploring the Antiglioma Mechanisms of Luteolin Based on Network Pharmacology and Experimental Verification
Source: Evid Based Complement Alternat Med. 2021 Nov 27;2021:7765658. doi: 10.1155/2021/7765658 (PMC8643232; doi:10.1155/2021/7765658)
Supplement: Supplementary Materials — The Supplementary Materials to this article include the following: the prediction targets of luteolin, the prediction targets of glioma, the signaling pathways in KEGG enrichment analysis of glioma-related targets, the result of GO enrichment analysis, and the 49 signaling pathways in KEGG pathway analysis for the targets of luteolin in glioma cells. [file 7765658.f1.zip › 7765658.f1/Supplementary Material (2).pdf]

Supplementary Material

Exploring the anti-glioma mechanisms of luteolin based on network pharmacology and experimental verification

Renxuan Huang, Rui Dong, Nan Wang, Beiwu Lan, Hongyang Zhao, Yufei Gao

Table S1. Prediction targets of luteolin.

Uniport id	Gene Symbols
P33527	ABCC1
Q92887	ABCC2
Q9UNQ0	ABCG2
P22303	ACHE
P09917	ALOX5
P08243	ASNS
Q07812	BAX
Q07817	BCL2L1
Q13490	BIRC2
P29466	CASP1
P42574	CASP3
P55210	CASP7
P55211	CASP9
P04040	CAT
P13500	CCL2
O76076	CCN5
P20248	CCNA2
P14635	CCNB1
P24385	CCND1
P29965	CD40LG
P04233	CD74
P19022	CDH2
P33151	CDH5
P46527	CDKN1B
P42773	CDKN2C
O15111	CHUK
P04141	CSF2

P48061	CXCL12
Q07325	CXCL9
P05177	CYP1A2
Q16678	CYP1B1
P33261	CYP2C19
P11712	CYP2C9
P05181	CYP2E1
P08684	CYP3A4
P20815	CYP3A5
P35638	DDIT3
P01133	EGF
P18146	EGR1
P05198	EIF2S1
O75460	ERN1
Q9Y624	F11R
P49327	FASN
P09467	FBP1
P01100	FOS
P53539	FOSB
O43524	FOXO3
O75293	GADD45B
P48507	GCLM
P14136	GFAP
Q9HC97	GPR35
P07203	GPX1
Q99075	HBEGF
Q16665	HIF1A
P09601	HMOX1
P11021	HSPA5
P05362	ICAM1
P01574	IFNB1
P01579	IFNG
P22301	IL10
P29460	IL12B
P35225	IL13
Q16552	IL17A
P01584	IL1B
Q8NEV9	IL27
P08700	IL3
P05113	IL5
P06213	INSR
Q14653	IRF3
P05412	JUN

P17275	JUNB
P17535	JUND
P21397	MAOA
P28482	MAPK1
P27361	MAPK3
P45984	MAPK9
P45452	MMP13
P14780	MMP9
P05164	MPO
O94916	NFAT5
Q16236	NFE2L2
P25963	NFKBIA
P35228	NOS2
P29474	NOS3
Q99571	P2RX4
P01127	PDGFB
P14618	PKM
P53350	PLK1
P17252	PRKCA
P35354	PTGS2
Q05397	PTK2
Q9ULZ3	PYCARD
Q04206	RELA
P16581	SELE
P58004	SESN2
P31947	SFN
Q4U2R8	SLC22A6
P11169	SLC2A3
P14672	SLC2A4
P13866	SLC5A1
P00441	SOD1
P04179	SOD2
P36956	SREBF1
P40763	STAT3
Q9UHD2	TBK1
P01137	TGFB1
P01375	TNF
P21580	TNFAIP3
P50591	TNFSF10
P11387	TOP1
Q53FA7	TP53I3
Q9H3D4	TP63
O15350	TP73

Q16881	TXNRD1
P22309	UGT1A1
P35503	UGT1A3
Q9UKW4	VAV3
P19320	VCAM1
P04275	VWF
P56703	WNT3
P17861	XBP1
P47989	XDH
P98170	XIAP
Q9NPH5	NOX4
P15121	AKR1B1
Q15078	CDK5R1
Q00535	CDK5
P36888	FLT3
P00918	CA2
Q8WWL7	CCNB3
P06493	CDK1
O95067	CCNB2
P30542	ADORA1
P43166	CA7
Q04760	GLO1
P05067	APP
P43405	SYK
P49841	GSK3B
P09874	PARP1
P02766	TTR
O43570	CA12
P08253	MMP2
P22748	CA4
P39900	MMP12
P28907	CD38
O60218	AKR1B10
Q9H2K2	TNKS2
O95271	TNKS
P05089	ARG1
Q13332	PTPRS
P14061	HSD17B1
Q00534	CDK6
P08183	ABCB1
P37059	HSD17B2
P16050	ALOX15
P18054	ALOX12

Q92731	ESR2
P11511	CYP19A1
P29274	ADORA2A
P68400	CSNK2A1
P03372	ESR1
P13569	CFTR
P04745	AMY1B
P43250	GRK6
P24941	CDK2
O14746	TERT
P00915	CA1
Q16790	CA9
P14679	TYR
P35869	AHR
P11474	ESRRA
P53355	DAPK1
P30518	AVPR2
P08069	IGF1R
P00533	EGFR
P00734	F2
P11309	PIM1
Q96GD4	AURKB
P21917	DRD4
P27986	PIK3R1
P06737	PYGL
P12931	SRC
P35968	KDR
P08254	MMP3
P07451	CA3
P23280	CA6
Q16512	PKN1
Q9ULX7	CA14
P08581	MET
P51955	NEK2
P25024	CXCR1
Q13554	CAMK2B
Q9UM73	ALK
P31749	AKT1
Q9HC98	NEK6
P04054	PLA2G1B
P35218	CA5A
P56817	BACE1
P30530	AXL

O60285	NUAK1
P52895	AKR1C2
Q04828	AKR1C1
P42330	AKR1C3
P17516	AKR1C4
Q8N1Q1	CA13
P14550	AKR1A1
Q16875	PFKFB3
B2RXH2	KDM4E
P00747	PLG
P10275	AR
P01834	IGKC
P01857	IGHG1
P0CG47	UBB
P62987	UBA52
P62979	RPS27A
P0CG48	UBC
P42684	ABL2
P55263	ADK
P02768	ALB
Q9NZN5	ARHGEF12
P18440	NAT1
O14815	CAPN9
Q9UKF6	CPSF3
O15075	DCLK1
O00338	SULT1C2
P06746	POLB
Q01094	E2F1
P48449	LSS
O95718	ESRRB
Q02790	FKBP4
Q01543	FLI1
P51570	GALK1
P10144	GZMB
P08631	HCK
Q92826	HOXB13
Q9HCP0	CSNK1G1
O94966	USP19
Q9NWW6	NMRK1
Q9H3U1	UNC45A
Q86UL8	MAGI2
Q9BYC5	FUT8
Q9BZI7	UPF3B

Q14449	GRB14
O00151	PDLIM1
Q53H47	SETMAR
Q96C23	GALM
Q96SI9	STRBP
O14936	CASK
Q9BZL6	PRKD2
Q9Y5X1	SNX9
Q9BZC1	CELF4
Q9H2C0	GAN
P49765	VEGFB
Q9UPY3	DICER1
Q9Y265	RUVBL1
Q9Y696	CLIC4
Q9Y6I3	EPN1
P14091	CTSE
P28329	CHAT
Q96QZ7	MAGI1
Q6PML9	SLC30A9
Q9BV79	MECR
Q6NVY1	HIBCH
Q93034	CUL5
O76064	RNF8
P12694	BCKDHA
O75351	VPS4B
P32455	GBP1
P52564	MAP2K6
Q96H20	SNF8
Q9HCB6	SPON1
Q13705	ACVR2B
P19623	SRM
Q9BY41	HDAC8
P10586	PTPRF

Table S2. Prediction targets of glioma.

Uniport id	Gene Symbols
Q9NQ94	A1CF
O95477	ABCA1
P78363	ABCA4
P08183	ABCB1
P21439	ABCB4
Q9NP58	ABCB6

P33527	ABCC1
O15438	ABCC3
O15439	ABCC4
O15440	ABCC5
O95255	ABCC6
P61221	ABCE1
Q9UNQ0	ABCG2
P24752	ACAT1
P12821	ACE
P25106	ACKR3
A6NK06	ACOD1
Q8WXI4	ACOT11
O00154	ACOT7
P15309	ACP3
Q8NEB7	ACRBP
Q96GR2	ACSBG1
P33121	ACSL1
O95573	ACSL3
O60488	ACSL4
Q9ULC5	ACSL5
Q9NR19	ACSS2
P60709	ACTB
Q562R1	ACTBL2
P68032	ACTC1
P63261	ACTG1
P63267	ACTG2
O96019	ACTL6A
O43707	ACTN4
P61160	ACTR2
Q04771	ACVR1
P36896	ACVR1B
P14621	ACYP2
P00813	ADA
Q9NZK5	ADA2
O14672	ADAM10
P78536	ADAM17
Q9P0K1	ADAM22
O75077	ADAM23
Q9UKQ2	ADAM28
Q13443	ADAM9
Q9UHI8	ADAMTS1
Q9UNA0	ADAMTS5
Q9UP79	ADAMTS8

P55265	ADAR
P78563	ADARB1
Q9NS39	ADARB2
Q96PN6	ADCY10
Q08462	ADCY2
P40145	ADCY8
P18509	ADCYAP1
P41586	ADCYAP1R1
Q8N7X0	ADGB
O14514	ADGRB1
Q9UHX3	ADGRE2
Q9BY15	ADGRE3
P48960	ADGRE5
Q5T601	ADGRF1
Q9Y653	ADGRG1
Q9HBW9	ADGRL4
Q15848	ADIPOQ
Q96A54	ADIPOR1
P55263	ADK
P35318	ADM
P30542	ADORA1
P29274	ADORA2A
P25100	ADRA1D
P35348	ADRA1A
P18089	ADRA2B
P07550	ADRB2
P30566	ADSL
Q8IUX7	AEBP1
Q99490	AGAP2
Q15109	AGER
P52594	AGFG1
Q8N302	AGGF1
Q53H12	AGK
Q9UKV8	AGO2
O00116	AGPS
Q09666	AHNAK
P35869	AHR
O95433	AHSA1
Q9GZX7	AICDA
P55008	AIF1
O95831	AIFM1
O14862	AIM2
Q12904	AIMP1

Q13155	AIMP2
Q9UKB5	AJAP1
Q96IF1	AJUBA
Q02952	AKAP12
Q13023	AKAP6
P15121	AKR1B1
P31749	AKT1
Q96B36	AKT1S1
P31751	AKT2
Q9Y243	AKT3
P13716	ALAD
P02768	ALB
Q8TES7	FBF1
P00352	ALDH1A1
P47895	ALDH1A3
Q9UM73	ALK
Q9UHK6	AMACR
P02760	AMBP
P23109	AMPD1
P03950	ANG
Q15389	ANGPT1
O15123	ANGPT2
Q9Y264	ANGPT4
O95841	ANGPTL1
Q9UKU9	ANGPTL2
Q8N2N9	ANKRD36B
Q8WVL7	ANKRD49
Q5XXA6	ANO1
Q4KMQ2	ANO6
P39687	ANP32A
P15144	ANPEP
P58335	ANTXR2
P04083	ANXA1
P07355	ANXA2
P08758	ANXA5
P08133	ANXA6
P20073	ANXA7
O95782	AP2A1
O14727	APAF1
P25054	APC
O95996	APC2
Q9UJX6	ANAPC2
Q9ULZ1	APLN

P35414	APLNR
Q9UH17	APOBEC3B
P02655	APOC2
P05090	APOD
P02649	APOE
P05067	APP
Q06481	APLP2
Q8NEU8	APPL2
P07741	APRT
P29972	AQP1
P55087	AQP4
O14520	AQP7
O43315	AQP9
P10275	AR
P10398	ARAF
O60936	NOL3
Q7LC44	ARC
P48444	ARCN1
P15514	AREG
P62330	ARF6
Q53QZ3	ARHGAP15
Q8N264	ARHGAP24
Q9NRY4	ARHGAP35
Q9BRR9	ARHGAP9
P52565	ARHGDIA
O15085	ARHGEF11
Q92974	ARHGEF2
Q96DR7	ARHGEF26
Q8N1W1	ARHGEF28
Q15052	ARHGEF6
Q14155	ARHGEF7
P36404	ARL2
P36405	ARL3
P40617	ARL4A
P49703	ARL4D
O00327	ARNTL
P52961	ART1
Q8N9N2	ASCC1
P50553	ASCL1
Q16515	ASIC2
Q9UHC3	ASIC3
Q96FT7	ASIC4
P04424	ASL

P45381	ASPA
Q86U10	ASPG
Q8IZT6	ASPM
P15336	ATF2
P18848	ATF4
Q9Y2D1	ATF5
P18850	ATF6
Q8WYN0	ATG4A
Q96DT6	ATG4C
Q86TL0	ATG4D
O95352	ATG7
P31939	ATIC
Q13315	ATM
P54259	ATN1
P05023	ATP1A1
P13637	ATP1A3
P14415	ATP1B2
Q9Y6H3	ATP23
O75787	ATP6AP2
Q04656	ATP7A
P20848	SERPINA2
Q13535	ATR
Q9H6X2	ANTXR1
O75882	ATRN
O14965	AURKA
Q96GD4	AURKB
Q9Y2T1	AXIN2
P30530	AXL
Q96A70	AZIN2
O43286	B4GALT5
Q9NXR7	BABAM2
Q92934	BAD
Q99933	BAG1
O95817	BAG3
Q13145	BAMBI
Q92560	BAP1
Q96QZ7	MAGI1
Q9NR55	BATF3
Q07812	BAX
Q96GW7	BCAN
P51572	BCAP31
P56945	BCAR1
P54687	BCAT1

O95999	BCL10
Q9C0K0	BCL11B
P10415	BCL2
Q07817	BCL2L1
Q9HB09	BCL2L12
Q92843	BCL2L2
P20749	BCL3
P41182	BCL6
Q6W2J9	BCOR
Q02338	BDH1
P30411	BDKRB2
P23560	BDNF
Q14457	BECN1
Q9BXY8	BEX2
P21810	BGN
Q8NFJ8	BHLHE22
O14503	BHLHE40
Q9C0J9	BHLHE41
Q9NZM4	BICRA
Q13489	BIRC3
O15392	BIRC5
P35226	BMI1
P12644	BMP4
P18075	BMP7
Q13873	BMPR2
Q12983	BNIP3
Q12830	BPTF
P15056	BRAF
Q6UWU4	C6orf89
Q7Z569	BRAP
P38398	BRCA1
P51587	BRCA2
P46736	BRCC3
O60885	BRD4
Q9NPI1	BRD7
Q9H0E9	BRD8
Q9HCU9	BRMS1
Q5PSV4	BRMS1L
P32247	BRS3
P35613	BSG
Q9BSF8	BTBD10
O00478	BTN3A3
P20290	BTF3

P78410	BTN3A2
P62324	BTG1
P78543	BTG2
Q06187	BTK
Q9Y297	BTRC
O43683	BUB1
Q9H8K7	PAAT
Q13901	C1D
Q07021	C1QBP
Q0P641	C2orf80
Q9NS85	CA10
O75493	CA11
Q9NS71	GKN1
P00918	CA2
Q16790	CA9
Q9Y376	CAB39
Q8IZS8	CACNA2D3
Q9HB71	CACYBP
Q9BY67	CADM1
Q8N3J6	CADM2
Q8N126	CADM3
Q8NFZ8	CADM4
P01258	CALCA
P06881	CALCA
P30988	CALCR
Q05682	CALD1
P0DP23	CALM1
P0DP24	CALM2
P0DP25	CALM3
P27482	CALML3
P27797	CALR
Q13554	CAMK2B
Q13555	CAMK2G
Q96RR4	CAMKK2
Q7Z624	CAMKMT
P49913	CAMP
Q96JM3	CHAMP1
P40121	CAPG
P04632	CAPNS1
Q9BWT7	CARD10
Q9BXL7	CARD11
Q6XLA1	CASC2
Q8IU53	CASC2

O14936	CASK
P07498	CSN3
P29466	CASP1
P42575	CASP2
P42574	CASP3
P55212	CASP6
Q14790	CASP8
P55211	CASP9
Q86V15	CASZ1
P04040	CAT
Q03135	CAV1
P51636	CAV2
Q6NZI2	CAVIN1
O95810	CAVIN2
P22681	CBL
Q13185	CBX3
P45973	CBX5
O95931	CBX7
Q8TAB7	CCDC26
P06307	CCK
P55774	CCL18
P13500	CCL2
P78556	CCL20
O00626	CCL22
P13501	CCL5
P80075	CCL8
O00622	CCN1
P29279	CCN2
P78396	CCNA1
P14635	CCNB1
O95067	CCNB2
P24385	CCND1
P30279	CCND2
P30281	CCND3
P24864	CCNE1
P41002	CCNF
Q16589	CCNG2
P51946	CCNH
P22674	CCNO
Q8ND76	CCNY
P46092	CCR10
P51679	CCR4
Q9UK39	NOCT

Q9ULM6	CNOT6
P51681	CCR5
P51684	CCR6
P32248	CCR7
P50991	CCT4
P50990	CCT8
Q6YHK3	CD109
P48509	CD151
Q86VB7	CD163
Q04900	CD164
P06729	CD2
P41217	CD200
P25063	CD24
P20963	CD247
P26842	CD27
Q9NZQ7	CD274
Q5ZPR3	CD276
P10747	CD28
P28906	CD34
P25942	CD40
P29965	CD40LG
P16070	CD44
P15529	CD46
Q08722	CD47
P09326	CD48
P34810	CD68
P32970	CD70
P04233	CD74
P33681	CD80
P27701	CD82
P42081	CD86
Q9NPY3	CD93
P14209	CD99
Q12834	CDC20
P30304	CDC25A
P30305	CDC25B
P30307	CDC25C
P60953	CDC42
O75419	CDC45
Q96GN5	CDCA7L
P12830	CDH1
Q9UM11	FZR1
P55287	CDH11

P55290	CDH13
Q13634	CDH18
P19022	CDH2
Q9UJ99	CDH22
P55283	CDH4
P33151	CDH5
P06493	CDK1
Q15131	CDK10
O94921	CDK14
Q96Q40	CDK15
Q00536	CDK16
P24941	CDK2
O14519	CDK2AP1
O75956	CDK2AP2
P11802	CDK4
Q00535	CDK5
Q00534	CDK6
P50613	CDK7
P49336	CDK8
P38936	CDKN1A
P46527	CDKN1B
P49918	CDKN1C
P42771	CDKN2A
Q8N726	CDKN2A
P42772	CDKN2B
P42773	CDKN2C
P55273	CDKN2D
Q16667	CDKN3
Q99626	CDX2
P13688	CEACAM1
P06731	CEACAM5
P49715	CEBPA
P17676	CEBPB
P49716	CEBPD
Q03701	CEBPZ
Q92879	CELF1
O95319	CELF2
Q9HC77	CENPJ
Q71F23	CENPU
Q6ZU80	CEP128
Q53EZ4	CEP55
P27544	CERS1
O00748	CES2

Q9P2B7	CFAP97
P08603	CFH
P23528	CFL1
O15519	CFLAR
Q9BUX1	CHAC1
Q13111	CHAF1A
O14646	CHD1
Q14839	CHD4
O00258	GET1
Q8TDI0	CHD5
Q9P2D1	CHD7
O14757	CHEK1
O96017	CHEK2
P36222	CHI3L1
P35790	CHKA
O00533	CHL1
Q96FC9	DDX11
Q8IZ52	CHPF
Q8WUD6	CHPT1
Q70JA7	CHSY3
Q9P2E5	CHPF2
Q99828	CIB1
Q96RK0	CIC
P33076	CIITA
Q9UPZ9	CILK1
Q8N5K1	CISD2
Q9NSE2	CISH
Q96RK1	CITED4
Q8WWK9	CKAP2
Q07065	CKAP4
Q14008	CKAP5
Q05315	CLC
Q9UBD9	CLCF1
P51788	CLCN2
P51790	CLCN3
P51793	CLCN4
P51795	CLCN5
P51798	CLCN7
O00501	CLDN5
Q9NY25	CLEC5A
O00299	CLIC1
Q9Y696	CLIC4
P49759	CLK1

O15516	CLOCK
Q96KA5	CLPTM1L
Q8IY22	CMIP
P30085	CMPK1
Q5EBM0	CMPK2
Q9NX76	CMTM6
P21554	CNR1
P26441	CNTF
P26992	CNTFR
Q12860	CNTN1
Q02246	CNTN2
Q9UHC6	CNTNAP2
P13942	COL11A2
P39060	COL18A1
P02461	COL3A1
P02462	COL4A1
Q01955	COL4A3
P20849	COL9A1
Q14055	COL9A2
Q14050	COL9A3
P49747	COMP
Q8NHY2	COP1
Q9Y678	COPG1
Q92905	COPS5
Q14019	COTL1
P00403	MT-CO2
P13073	COX4I1
P20674	COX5A
P10606	COX5B
P14406	COX7A2
P10176	COX8A
P00450	CP
Q9UI42	CPA4
P16870	CPE
Q9BZB8	CPEB1
Q8NE35	CPEB3
Q17RY0	CPEB4
P36551	CPOX
Q9Y646	CPQ
P16220	CREB1
Q96BA8	CREB3L1
P06850	CRH
P34998	CRHR1

P16562	CRISP2
P46108	CRK
Q8IUI8	CRLF3
Q14194	CRMP1
P02741	CRP
Q16526	CRY1
Q49AN0	CRY2
P02511	CRYAB
P09603	CSF1
P07333	CSF1R
P04141	CSF2
P09919	CSF3
P41240	CSK
Q6UVK1	CSPG4
P50461	CSRP3
P01034	CST3
Q15828	CST6
P78358	CTAG1A
Q13363	CTBP1
P56545	CTBP2
Q2NKJ3	CTC1
P16410	CTLA4
P35222	CTNNB1
Q9NSA3	CTNNBIP1
O60716	CTNND1
Q9UQB3	CTNND2
P07858	CTSB
P07339	CTSD
P43235	CTSK
P07711	CTSL
Q14247	CTTN
Q9H467	CUEDC2
Q13616	CUL1
P39880	CUX1
Q13948	CUX1
P49238	CX3CR1
P09341	CXCL1
P02778	CXCL10
O14625	CXCL11
P48061	CXCL12
O95715	CXCL14
Q9H2A7	CXCL16
Q6UXB2	CXCL17

P10145	CXCL8
Q07325	CXCL9
P25025	CXCR2
P49682	CXCR3
P61073	CXCR4
O00574	CXCR6
P08574	CYC1
Q8WWM9	CYGB
Q9NQC7	CYLD
P05093	CYP17A1
P11511	CYP19A1
P04798	CYP1A1
Q16678	CYP1B1
O15528	CYP27B1
P20813	CYP2B6
P11712	CYP2C9
P10635	CYP2D6
P05181	CYP2E1
P51589	CYP2J2
P13584	CYP4B1
Q8N118	CYP4X1
Q86T65	DAAM2
Q9UI36	DACH1
Q5SW24	DACT2
Q14118	DAG1
P0C864	DANCR
P14920	DAO
O95886	DLGAP3
P51398	DAP3
Q9UIK4	DAPK2
Q9UER7	DAXX
Q16643	DBN1
Q10586	DBP
P11182	DBT
Q9Y4B6	DCAF1
P43146	DCC
P27707	DCK
P07585	DCN
P40126	DCT
Q9UJW0	DCTN4
O00399	DCTN6
Q96GG9	DCUN1D1
O43602	DCX

P0DPQ6	DDIT3
P35638	DDIT3
Q9NX09	DDIT4
Q08345	DDR1
Q9BUQ8	DDX23
P17844	DDX5
Q5T1V6	DDX59
P26196	DDX6
Q16698	DECR1
Q9P2X7	DELEC1
O43583	DENR
Q5TB30	DEPDC1
Q9BUN8	DERL1
P17661	DES
Q15392	DHCR24
Q86SQ9	DHDDS
O43143	DHX15
Q9NR28	DIABLO
O60610	DIAPH1
Q9UPY3	DICER1
O95057	DIRAS1
O95661	DIRAS3
Q155Q3	DIXDC1
O60832	DKC1
O94907	DKK1
Q9UBP4	DKK3
O43261	DLEU1
P80370	DLK1
O00548	DLL1
Q9NYJ7	DLL3
Q9NR61	DLL4
Q9UGM3	DMBT1
Q9Y485	DMXL1
Q96EY1	DNAJA3
O14645	DNALI1
Q05193	DNM1
O00429	DNM1L
P50570	DNM2
Q9UQ16	DNM3
P26358	DNMT1
Q9Y6K1	DNMT3A
Q9UBC3	DNMT3B
Q14185	DOCK1

P16444	DPEP1
Q92785	DPF2
P27487	DPP4
P21918	DRD5
Q02487	DSC2
Q14574	DSC3
Q9NZW4	DSPP
P60981	DSTN
Q8TDB6	DTX3L
P28562	DUSP1
Q05923	DUSP2
Q9BV47	DUSP26
Q13115	DUSP4
O14640	DVL1
P54792	DVL1P1
O14641	DVL2
Q7RTS9	DYM
Q8NCM8	DYNC2H1
Q96FJ2	DYNLL2
Q13627	DYRK1A
Q92630	DYRK2
Q01094	E2F1
Q14209	E2F2
O00716	E2F3
Q96AV8	E2F7
O00559	EBAG9
Q14213	EBI3
Q15125	EBP
Q9H1Z8	ECRG4
Q9H8V3	ECT2
P05305	EDN1
P25101	EDNRA
P24530	EDNRB
P26641	EEF1G
O00418	EEF2K
Q12805	EFEMP1
P20827	EFNA1
P52799	EFNB2
Q15768	EFNB3
P01133	EGF
Q9UHF1	EGFL7
P00533	EGFR
Q96KS0	EGLN2

Q9H6Z9	EGLN3
P18146	EGR1
Q9BQI3	EIF2AK1
P56537	EIF6
Q14152	EIF3A
Q99613	EIF3C
O15371	EIF3D
Q13347	EIF3I
Q9UBQ5	EIF3K
P06730	EIF4E
Q13542	EIF4EBP2
Q15717	ELAVL1
Q99607	ELF4
P19419	ELK1
P28324	ELK4
O00472	ELL2
Q92556	ELMO1
Q9NXB9	ELOVL2
Q9NYP7	ELOVL5
Q5UCC4	EMC10
P54849	EMP1
P54851	EMP2
P54852	EMP3
Q04743	EMX2
P19622	EN2
P17813	ENG
P06733	ENO1
Q9UHY7	ENOPH1
Q7L5Y1	ENOSF1
P49961	ENTPD1
Q99814	EPAS1
P21709	EPHA1
P29317	EPHA2
P29320	EPHA3
P54764	EPHA4
P54756	EPHA5
P29322	EPHA8
P29323	EPHB2
P54760	EPHB4
Q9H201	EPN3
P01588	EPO
P11678	EPX
P19235	EPOR

Q12929	EPS8
Q8TE68	EPS8L1
P04626	ERBB2
P21860	ERBB3
P07992	ERCC1
P18074	ERCC2
Q92889	ERCC4
P28715	ERCC5
O14944	EREG
O75460	ERN1
Q69383	ERVK-6
Q69384	ERVK-6
Q7LDI9	ERVK-6
Q9BXR3	ERVK-6
Q9Y6I0	ERVK-6
Q9UQF0	ERVW-1
Q9NQ30	ESM1
P03372	ESR1
Q92731	ESR2
P11474	ESRRA
P13804	ETFA
P14921	ETS1
P43268	ETV4
Q16394	EXT1
O95677	EYA4
Q92800	EZH1
Q15910	EZH2
P15311	EZR
P00742	F10
Q9BQ89	FAM110A
Q9Y624	F11R
P00734	F2
P25116	F2R
P55085	F2RL1
P13726	F3
O15540	FABP7
Q13158	FADD
O95990	FAM107A
Q8N9E0	FAM133A
A1KXE4	FAM168B
Q8NEG4	FAM83F
Q9BXW9	FANCD2
Q12884	FAP

P25445	FAS
P48023	FASLG
P49327	FASN
Q14517	FAT1
Q8WUP2	FBLIM1
P09467	FBP1
Q9HAH7	FBRS
Q96ME1	FBXL18
Q9NRD0	FBXO8
Q969H0	FBXW7
P12319	FCER1A
P12314	FCGR1A
Q92637	FCGR1B
P22570	FDXR
P22830	FECH
P39748	FEN1
A9Z1Z3	FER1L4
Q96AC1	FERMT2
Q86UX7	FERMT3
A0PJY2	FEZF1
P05230	FGF1
Q92913	FGF13
P09038	FGF2
P08620	FGF4
P21781	FGF7
P31371	FGF9
P21802	FGFR2
P22607	FGFR3
Q14314	FGL2
Q14192	FHL2
Q13643	FHL3
Q5TD97	FHL5
Q2V2M9	FHOD3
Q8IVF7	FMNL3
Q9Y3D6	FIS1
Q02790	FKBP4
Q13451	FKBP5
P11362	FGFR1
P20930	FLG
O75369	FLNB
Q14315	FLNC
Q14254	FLOT2
P17948	FLT1

P49771	FLT3LG
P35916	FLT4
Q8TAF5	FLVCR1-DT
Q06828	FMOD
Q8N0W7	FMR1NB
P02751	FN1
Q9Y2H6	FNDC3A
Q5VW36	FOCAD
Q04609	FOLH1
P01100	FOS
P53539	FOSB
P15407	FOSL1
P15408	FOSL2
P55317	FOXA1
Q9Y261	FOXA2
Q12948	FOXC1
Q99958	FOXC2
Q16676	FOXD1
O60548	FOXD2
P55316	FOXG1
P85037	FOXK1
Q01167	FOXK2
Q12952	FOXL1
Q12778	FOXO1
O43524	FOXO3
Q9H334	FOXP1
Q9BZS1	FOXP3
Q9C009	FOXQ1
Q6PJQ5	FOXR2
Q05932	FPGS
P21462	FPR1
P25090	FPR2
Q92837	FRAT1
P42685	FRK
Q9BTV5	FSD1
Q9BXM9	FSD1L
Q12841	FSTL1
P02794	FTH1
Q96AE4	FUBP1
P09958	FURIN
P35637	FUS
P22083	FUT4
Q16595	FXN

Q14332	FZD2
Q9ULV1	FZD4
O75084	FZD7
P27469	G0S2
P11413	G6PD
Q13480	GAB1
Q9UQC2	GAB2
Q8WWW8	GAB3
Q06546	GABPA
Q99259	GAD1
P24522	GADD45A
Q6ZQY3	GADL1
P22466	GAL
Q8N6F7	GCSAM
Q99999	GAL3ST1
Q10471	GALNT2
Q86SF2	GALNT7
P17677	GAP43
P04406	GAPDH
P54826	GAS1
Q14393	GAS6
P01350	GAST
P23769	GATA2
Q92908	GATA6
Q8WUU5	GATAD1
Q8WXI9	GATAD2B
P0DPI2	GATD3A
P32455	GBP1
Q8WXF7	ATL1
Q9H0R5	GBP3
P48506	GCLC
P48507	GCLM
Q9NZC3	GDE1
Q99988	GDF15
P39905	GDNF
Q9H840	GEMIN7
P14136	GFAP
O75223	GGCT
Q9H3C7	GGNBP2
P01241	GH1
Q9Y248	GINS2
O14908	GIPC1
P17302	GJA1

P29033	GJB2
O95452	GJB6
P16278	GLB1
P23378	GLDC
P08151	GLI1
P10070	GLI2
P10071	GLI3
P48060	GLIPR1
Q9H4G4	GLIPR2
Q04760	GLO1
P43220	GLP1R
O94925	GLS
Q9UI32	GLS2
P00367	GLUD1
P49448	GLUD2
P15104	GLUL
P30968	GNRHR
Q08378	GOLGA3
Q7Z5G4	GOLGA7
Q8NBJ4	GOLM1
Q9H4A6	GOLPH3
Q9HD26	GOPC
Q9BQQ3	GORASP1
O95249	GOSR1
P35052	GPC1
P78333	GPC5
P21695	GPD1
P43304	GPD2
P06744	GPI
Q5T848	GPR158
O60883	GPR37L1
O15529	GPR42
Q8IYL9	GPR65
P07203	GPX1
P36969	GPX4
O75791	GRAP2
O60565	GREM1
P42261	GRIA1
Q13224	GRIN2B
P34947	GRK5
P43250	GRK6
Q13255	GRM1
Q14832	GRM3

O00222	GRM8
P56915	GSC
Q9BYG8	GSDMC
P49841	GSK3B
P06396	GSN
Q9Y2Q3	GSTK1
P09488	GSTM1
P28161	GSTM2
P21266	GSTM3
P78417	GSTO1
P09211	GSTP1
P30711	GSTT1
P32780	GTF2H1
Q02108	GUCY1A1
Q02153	GUCY1B1
Q6FI13	H2AC18
Q16777	H2AC20
P16104	H2AX
P84243	H3-3A
P68431	H3C1
P62805	H4C1
P27695	APEX1
P54257	HAP1
Q86UW8	HAPLN4
Q92819	HAS2
Q8TDQ0	HAVCR2
O00165	HAX1
Q99075	HBEGF
O60381	HBP1
Q8IV16	GPIHBP1
Q9BXC0	HCAR1
Q9HCI5	MAGEE1
P14317	HCLS1
Q13547	HDAC1
Q92769	HDAC2
O15379	HDAC3
P56524	HDAC4
Q9UQL6	HDAC5
Q9UBN7	HDAC6
P51858	HDGF
Q9H583	HEATR1
Q7Z4Q2	HEATR3
Q6AI08	HEATR6

Q9NRZ9	HELLS
Q9UII4	HERC5
Q15011	HERPUD1
A0A0B4J2D5	GATD3B
Q14469	HES1
Q5TA89	HES5
Q96HZ4	HES6
P06865	HEXA
P07686	HEXB
Q30201	HFE
P14210	HGF
Q5VTY9	HHAT
Q96QV1	HHIP
Q9UM44	HHLA2
Q14526	HIC1
Q16665	HIF1A
Q9H2X6	HIPK2
Q9H422	HIPK3
P31629	HIVEP2
P19367	HK1
P52789	HK2
P04439	HLA-A
P01889	HLA-B
P10321	HLA-C
P28067	HLA-DMA
P01909	HLA-DQA1
P01911	HLA-DRB1
P13747	HLA-E
P30511	HLA-F
P17693	HLA-G
Q6NT76	HMBOX1
Q9P0W2	HMG20B
P17096	HMGA1
P52926	HMGA2
P09429	HMGB1
P26583	HMGB2
P04035	HMGCR
P82970	HMGN5
O75330	HMMR
P09601	HMOX1
P41235	HNF4A
P09651	HNRNPA1
P07910	HNRNPC

O14979	HNRNPDL
P52597	HNRNPF
P61978	HNRNPK
Q00839	HNRNPU
Q96ED9	HOOK2
P31260	HOXA10
P31270	HOXA11
P31271	HOXA13
O43365	HOXA3
Q00056	HOXA4
P20719	HOXA5
P31268	HOXA7
P31269	HOXA9
P14653	HOXB1
Q92826	HOXB13
P14651	HOXB3
P09629	HOXB7
P17482	HOXB9
Q9NYD6	HOXC10
P09017	HOXC4
P09630	HOXC6
P31273	HOXC8
P28358	HOXD10
P09016	HOXD4
P28356	HOXD9
P00738	HP
O60760	HPGDS
P00739	HPR
Q9Y251	HPSE
P01112	HRAS
P23327	HRC
P04196	HRG
Q6YN16	HSDL2
Q00613	HSF1
P07900	HSP90AA1
Q14568	HSP90AA2P
P14625	HSP90B1
P0DMV8	HSPA1A
P0DMV9	HSPA1B
P34932	HSPA4
P11021	HSPA5
P11142	HSPA8
P04792	HSPB1

Q16082	HSPB2
Q12988	HSPB3
P98160	HSPG2
Q9BUP3	HTATIP2
P08908	HTR1A
P28566	HTR1E
P28335	HTR2C
P47898	HTR5A
P50406	HTR6
Q12794	HYAL1
Q12891	HYAL2
O43820	HYAL3
P05362	ICAM1
O75144	ICOSLG
P41134	ID1
Q02363	ID2
P47928	ID4
O75874	IDH1
P48735	IDH2
P14902	IDO1
P46695	IER3
P40305	IFI27
P13284	IFI30
Q01628	IFITM3
P01562	IFNA1;
P01574	IFNB1
P01579	IFNG
P05019	IGF1
P08069	IGF1R
P01344	IGF2
Q9NZI8	IGF2BP1
Q9Y6M1	IGF2BP2
P08833	IGFBP1
P18065	IGFBP2
P17936	IGFBP3
P22692	IGFBP4
P24592	IGFBP6
Q16270	IGFBP7
Q8N6C5	IGSF1
Q70UQ0	IKBIP
O14920	IKBKB
Q14164	IKBKE
P22301	IL10

P29459	IL12A
P29460	IL12B
P35225	IL13
P78552	IL13RA1
Q14627	IL13RA2
P40933	IL15
Q14005	IL16
Q16552	IL17A
Q8TAD2	IL17D
Q96F46	IL17RA
Q8NFM7	IL17RD
Q14116	IL18
Q13478	IL18R1
P01583	IL1A
P01584	IL1B
P18510	IL1RN
P60568	IL2
Q9HBE4	IL21
Q9GZX6	IL22
Q969J5	IL22RA2
Q9NPF7	IL23A
Q13007	IL24
Q8NEV9	IL27
P01589	IL2RA
P14784	IL2RB
O95760	IL33
P05112	IL4
P24394	IL4R
P05113	IL5
P05231	IL6
P40189	IL6ST
P13232	IL7
Q12905	ILF2
Q13418	ILK
O00425	IGF2BP3
Q8TCT8	SPPL2A
Q9NV31	IMP3
P29218	IMPA1
Q16352	INA
Q9UK53	ING1
Q9H160	ING2
Q9UNL4	ING4
Q8WYH8	ING5

P01308	INS
P06213	INSR
Q9ULD6	INTU
P46940	IQGAP1
Q5JU85	IQSEC2
Q9NWZ3	IRAK4
P10914	IRF1
Q14653	IRF3
Q92985	IRF7
Q00978	IRF9
A1A4Y4	IRGM
P35568	IRS1
P78414	IRX1
Q96AZ6	ISG20
Q9NPH2	ISYNA1
P08514	ITGA2B
P26006	ITGA3
P13612	ITGA4
P08648	ITGA5
P06756	ITGAV
P20702	ITGAX
P05556	ITGB1
P05106	ITGB3
P16144	ITGB4
P18084	ITGB5
P18564	ITGB6
P26012	ITGB8
Q14624	ITIH4
Q08881	ITK
P23677	ITPKA
P28290	ITPRID2
Q15811	ITSN1
P78504	JAG1
P23458	JAK1
O60674	JAK2
Q9BX67	JAM3
Q92833	JARID2
Q8WYK2	JDP2
Q6NYC1	JMJD6
P05412	JUN
Q63ZY3	KANK2
Q92830	KAT2A
Q9H7Z6	KAT8

Q96I82	KAZALD1
Q16322	KCNA10
P22001	KCNA3
P22460	KCNA5
Q14721	KCNB1
O95259	KCNH1
O00180	KCNK1
Q12791	KCNMA1
Q16558	KCNMB1
Q14681	KCTD2
O60341	KDM1A
Q8NHM5	KDM2B
O75164	KDM4A
P29375	KDM5A
Q9UGL1	KDM5B
O15054	KDM6B
P35968	KDR
Q14145	KEAP1
Q07666	KHDRBS1
P50053	KHK
Q92945	KHSRP
Q9HCM3	KIAA1549
Q15058	KIF14
O60333	KIF1B
O95235	KIF20A
Q02241	KIF23
Q2KJY2	KIF26B
O00139	KIF2A
Q99661	KIF2C
P33176	KIF5B
Q8N743	KIR3DL3
P10721	KIT
Q07866	KLC1
Q9Y2Y9	KLF13
Q9BXK1	KLF16
Q9Y5W3	KLF2
O43474	KLF4
Q13887	KLF5
Q99612	KLF6
O75840	KLF7
O95600	KLF8
Q13886	KLF9
P20151	KLK2

Q92876	KLK6
P26718	KLRK1
Q03164	KMT2A
Q9UMN6	KMT2B
Q8NEZ4	KMT2C
O14686	KMT2D
Q9NQR1	KMT5A
P01042	KNG1
P52292	KPNA2
Q14974	KPNB1
P01116	KRAS
O00522	KRIT1
P04264	KRT1
P32004	L1CAM
P83111	LACTB
Q16363	LAMA4
O15230	LAMA5
P07942	LAMB1
P55268	LAMB2
P11047	LAMC1
Q13753	LAMC2
Q9Y2Q5	LAMTOR2
Q86VI4	LAPTM4B
Q92615	LARP4B
Q9BRS8	LARP6
Q14847	LASP1
O43561	LAT
O95835	LATS1
Q9NRM7	LATS2
P18428	LBP
P06239	LCK
P80188	LCN2
P00338	LDHA
P01130	LDLR
P41159	LEP
O15243	LEPROT
P48357	LEPR
Q6P1Q0	LETMD1
P09382	LGALS1
Q8TCE9	LGALS14
P17931	LGALS3
O00182	LGALS9
O95970	LGI1

Q8N145	LGI3
Q8N135	LGI4
Q99538	LGMN
Q9BXB1	LGR4
O75473	LGR5
Q86UP9	LHFPL3
Q9NQ69	LHX9
P15018	LIF
P18858	LIG1
P49917	LIG4
Q8NHL6	LILRB1
P55344	LIM2
P48059	LIMS1
Q9H9Z2	LIN28A
Q5TKA1	LIN9
Q96N35	LINC00052
P59037	LINC00313
A8K010	LINC00473
Q96FQ7	LINC00526
Q99732	LITAF
Q15334	LLGL1
Q96S06	LMF1
P25791	LMO2
Q8TBB1	LNX1
P28300	LOX
Q9Y4K0	LOXL2
Q9HBW0	LPAR2
P06858	LPL
P02750	LRG1
Q96JA1	LRIG1
O94898	LRIG2
Q6UXM1	LRIG3
Q07954	LRP1
O75197	LRP5
Q6UY01	LRRC31
Q9HBW1	LRRC4
Q8IWT6	LRRC8A
P48449	LSS
P51884	LUM
P07948	LYN
P61626	LYZ
Q8N653	LZTR1
Q6ZN28	MACC1

Q9Y6D9	MAD1L1
Q13257	MAD2L1
Q9UI95	MAD2L2
P20916	MAG
P43356	MAGEA2
P43360	MAGEA6
Q9Y5V3	MAGED1
Q96JG8	MAGED4
Q5TCQ9	MAGI3
Q9H0U3	MAGT1
Q9BXY0	MAK16
Q9UDY8	MALT1
Q8IZL2	MAML2
P21397	MAOA
P27338	MAOB
P46821	MAP1B
Q9GZQ8	MAP1LC3B
P11137	MAP2
Q02750	MAP2K1
P36507	MAP2K2
P46734	MAP2K3
P45985	MAP2K4
O14733	MAP2K7
Q13233	MAP3K1
Q99558	MAP3K14
Q5TCX8	MAP3K21
P28482	MAPK1
Q16539	MAPK14
P27361	MAPK3
Q13164	MAPK7
P45983	MAPK8
Q9UQF2	MAPK8IP1
Q9BPZ7	MAPKAP1
Q5T0T0	MARCHF8
P29966	MARCKS
P49006	MARCKSL1
Q96L34	MARK4
P04201	MAS1
Q9UIS9	MBD1
Q9UBB5	MBD2
P11226	MBL2
P02686	MBP
Q14703	MBTPS1

P43121	MCAM
Q07820	MCL1
P49736	MCM2
P25205	MCM3
Q14566	MCM6
P33993	MCM7
Q8IZK6	MCOLN2
Q8NEM0	MCPH1
Q96EZ8	MCRS1
Q9NWR8	MCUB
Q14676	MDC1
P21741	MDK
Q00987	MDM2
O15151	MDM4
P48163	ME1
Q03112	MECOM
P51608	MECP2
O95243	MBD4
Q15648	MED1
Q71SY5	MED25
Q9NWA0	MED9
Q06413	MEF2C
Q14814	MEF2D
O15553	MEFV
Q96KG7	MEGF10
Q14680	MELK
P08582	MELTF
O00255	MEN1
P50222	MEOX2
Q12866	MERTK
P08581	MET
Q9NWH9	SLTM
P50579	METAP2
Q86U44	METTL3
P55081	MFAP1
P26572	MGAT1
Q09328	MGAT5
P16455	MGMT
P08493	MGP
Q16674	MIA
Q86YT6	MIB1
Q96AX9	MIB2
Q29983	MICA

Q5JXC2	MIIP
Q4G0A6	MINDY4
Q75NE6	MIR17HG
O75030	MITF
P46013	MKI67
Q9BUB5	MKNK1
Q9NXB0	MKS1
P40692	MLH1
Q9UHC1	MLH3
Q8NB16	MLKL
P55197	MLLT10
P08473	MME
P03956	MMP1
P39900	MMP12
P45452	MMP13
P50281	MMP14
P51511	MMP15
P51512	MMP16
P08253	MMP2
Q9NRE1	MMP26
P08254	MMP3
P09237	MMP7
P14780	MMP9
Q13201	MMRN1
P22033	MMUT
Q9UQ07	MOK
Q9HCE1	MOV10
Q9Y5U8	MPC1
O95563	MPC2
P29372	MPG
Q99547	MPHOSPH6
P34949	MPI
Q6WCQ1	MPRIP
P25189	MPZ
P22897	MRC1
Q9UBG0	MRC2
P49959	MRE11
Q13084	MRPL28
Q8TCC3	MRPL30
Q9Y6G3	MRPL42
O60682	MSC
P43246	MSH2
P20585	MSH3

P52701	MSH6
O43347	MSI1
P26038	MSN
P21757	MSR1
P26927	MST1
P04731	MT1A
P07438	MT1B
P04732	MT1E
P04733	MT1F
P80294	MT1H
Q93083	MT1L
P13640	MT1G
Q8N339	MT1M
P80297	MT1X
O94776	MTA2
Q9BTC8	MTA3
Q13126	MTAP
Q86UE4	MTDH
P13995	MTHFD2
P42898	MTHFR
Q13615	MTMR3
P48039	MTNR1A
P42345	MTOR
Q9UBK8	MTRR
P15941	MUC1
Q9UIF7	MUTYH
P53602	MVD
Q14764	MVP
P20591	MX1
Q05195	MXD1
P50539	MXI1
P10242	MYB
P10243	MYBL1
P10244	MYBL2
Q13203	MYBPH
P01106	MYC
Q99417	MYCBP
P04198	MYCN
P35580	MYH10
Q9UKX2	MYH2
P10916	MYL2
Q15746	MYLK
Q96A32	MYLPF

O43795	MYO1B
Q9UL68	MYT1L
Q14CX7	NAA25
Q96HR8	NAF1
P43490	NAMPT
Q9H9S0	NANOG
Q9NR45	NANS
P55209	NAP1L1
Q6XQN6	NAPRT
P11245	NAT2
Q6N069	NAA16
Q6ZS30	NBEAL1
O60934	NBN
P19878	NCF2
P19338	NCL
Q15788	NCOA1
O76036	NCR1
Q68D85	NCR3LG1
O14777	NDC80
Q9GZM8	NDEL1
Q92597	NDRG1
Q9UN36	NDRG2
Q9ULP0	NDRG4
Q9P0J0	NDUFA13
P46934	NEDD4
Q96PU5	NEDD4L
Q14511	NEDD9
P07196	NEFL
P51955	NEK2
P18615	NELFE
Q92859	NEO1
P48681	NES
P21359	NF1
P35240	NF2
O94916	NFAT5
O95644	NFATC1
Q13469	NFATC2
Q12968	NFATC3
Q14934	NFATC4
Q16236	NFE2L2
O00712	NFIB
Q16649	NFIL3
Q14938	NFIX

P19838	NFKB1
P25963	NFKBIA
Q9NPG2	NGB
P01138	NGF
P08138	NGFR
Q6T4R5	NHS
Q96TA1	NIBAN2
Q9NZG7	NINJ2
Q8N5F7	NKAP
P43699	NKX2-1
Q9NZ94	NLGN3
Q9UBE8	NLK
Q9NPP4	NLRC4
P59046	NLRP12
Q9NX02	NLRP2
Q96P20	NLRP3
P15531	NME1
Q13287	NMI
Q9ULX3	NOB1
Q9Y3T9	NOC2L
Q9HC29	NOD2
Q13253	NOG
Q15155	NOMO1
P46087	NOP2
Q9NZM5	NOP53
P29475	NOS1
Q8WY41	NANOS1
O75052	NOS1AP
P35228	NOS2
P60321	NANOS2
P46531	NOTCH1
Q04721	NOTCH2
Q9UM47	NOTCH3
P51513	NOVA1
Q9UNW9	NOVA2
Q9Y5S8	NOX1
Q9NPH5	NOX4
P06748	NPM1
P23582	NPPC
Q8WTW4	NPRL2
P01303	NPY
P15559	NQO1
O75469	NR1I2

P49116	NR2C2
Q9Y466	NR2E1
P24468	NR2F2
P04150	NR3C1
P01111	NRAS
Q16612	NREP
Q02297	NRG1
O14786	NRP1
Q92832	NELL1
O60462	NRP2
Q9HDB5	NRXN3
Q9Y4C0	NRXN3
Q96L73	NSD1
Q96P11	NSUN5
Q9H0P0	NT5C3A
Q9H857	NT5DC2
P21589	NT5E
P20783	NTF3
O95631	NTN1
P04629	NTRK1
Q16620	NTRK2
Q16288	NTRK3
P30990	NTS
P30989	NTSR1
P78423	CX3CL1
Q9H093	NUAK2
Q9BZD4	NUF2
Q9Y6R0	NUMBL
P37198	NUP62
P52948	NUP98
Q9BXS6	NUSAP1
Q9UKK6	NXT1
Q16625	OCLN
P11926	ODC1
O43482	OIP5
Q13516	OLIG2
O60313	OPA1
Q14982	OPCML
P41143	OPRD1
P41145	OPRK1
Q96CV9	OPTN
Q9H205	OR2AG1
Q96D31	ORAI1

Q96SN7	ORAI2
Q8N138	ORMDL3
P13725	OSM
Q99650	OSMR
P55809	OXCT1
Q99571	P2RX4
Q99572	P2RX7
P47900	P2RY1
Q9H244	P2RY12
Q15391	P2RY14
P13674	P4HA1
P07237	P4HB
P09466	PAEP
P00439	PAH
Q13153	PAK1
O96013	PAK4
Q9NQU5	PAK6
Q9P286	PAK5
O75592	MYCBP2
P19021	PAM
Q96RD6	PANX2
Q6TCH7	PAQR3
Q86WK9	PAQR7
Q99497	PARK7
P09874	PARP1
Q8IV76	PASD1
Q9HBE1	PATZ1
Q96IZ0	PAWR
P23760	PAX3
P26367	PAX6
Q9BUH6	PAXX
Q96KB5	PBK
Q9GZY1	PBOV1
P40426	PBX3
P61457	PCBD1
Q9H0N5	PCBD2
Q15366	PCBP2
Q9P2E7	PCDH10
O95206	PCDH8
Q9HC56	PCDH9
Q9Y5H2	PCDHGA11
Q96HM7	PCED1B
Q9BSM1	PCGF1

Q15004	PCLAF
P22061	PCMT1
P12004	PCNA
P29120	PCSK1
P29122	PCSK6
Q13442	PDAP1
Q15116	PDCD1
Q9BQ51	PDCD1LG2
Q53EL6	PDCD4
O14737	PDCD5
Q08493	PDE4C
O76074	PDE5A
P04085	PDGFA
P01127	PDGFB
P16234	PDGFRA
P09619	PDGFRB
P30101	PDIA3
Q8N165	PDIK1L
Q15118	PDK1
Q15120	PDK3
Q16654	PDK4
O00151	PDLIM1
Q96HC4	PDLIM5
Q86YL7	PDPN
Q6P996	PDXDC1
Q96GD0	PDXP
Q15121	PEA15
P30086	PEBP1
Q96S96	PEBP4
P16284	PECAM1
Q86TG7	PEG10
Q9GZU2	PEG3
O15534	PER1
O15055	PER2
P56645	PER3
P02776	PF4
Q16877	PFKFB4
P07737	PFN1
P18669	PGAM1
P00558	PGK1
Q9NXJ5	PGPEP1
O00264	PGRMC1
P35232	PHB

P78364	PHC1
P78562	PHEX
O94880	PHF14
Q5T6S3	PHF19
Q9BVI0	PHF20
Q96BD5	PHF21A
O43175	PHGDH
Q8WV24	PHLDA1
Q86UU1	PHLDB1
O60346	PHLPP1
Q6ZVD8	PHLPP2
Q96FC7	PHYHIPL
Q9Y6X2	PIAS3
Q7Z2X4	PID1
Q92508	PIEZO1
Q9H5I5	PIEZO2
O00750	PIK3C2B
P42336	PIK3CA
P42338	PIK3CB
O00329	PIK3CD
P48736	PIK3CG
P27986	PIK3R1
Q92569	PIK3R3
Q13526	PIN1
Q96BK5	PINX1
P78337	PITX1
Q96J94	PIWIL1
Q8TC59	PIWIL2
Q7Z3Z4	PIWIL4
P98161	PKD1
Q13563	PKD2
Q9P0L9	PKD2L1
P14618	PKM
Q99959	PKP2
Q9UP65	PLA2G4C
P39877	PLA2G5
Q9UL19	PLAAT4
P00750	PLAT
P00749	PLAU
Q03405	PLAUR
Q9NQ66	PLCB1
P19174	PLCG1
Q15111	PLCL1

Q13393	PLD1
O14939	PLD2
P08567	PLEK
Q96JA3	PLEKHA8
O95397	PLEKHA8P1
O94827	PLEKHG5
Q53GL0	PLEKHO1
P00747	PLG
P53350	PLK1
O00444	PLK4
O00469	PLOD2
O60568	PLOD3
P60201	PLP1
Q04941	PLP2
P55058	PLTP
Q9ULL4	PLXNB3
Q969W9	PMEPA1
Q01453	PMP22
Q9NR77	PXMP2
P54277	PMS1
P54278	PMS2
P16233	PNLIP
Q9NRX1	PNO1
Q96AD5	PNPLA2
P06746	POLB
P28340	POLD1
Q15054	POLD3
Q9HCU8	POLD4
Q9Y2S7	POLDIP2
Q9NR33	POLE4
Q9UBT6	POLK
Q9UGP5	POLL
Q9NP87	POLM
P24928	POLR2A
Q9NW08	POLR3B
P01189	POMC
P27169	PON1
P16435	POR
Q15063	POSTN
Q9NUX5	POT1
Q6S545	POTEH
Q9BYX7	POTEKP
A6NI47	POTEM

P20264	POU3F3
Q01860	POU5F1
Q07869	PPARA
P37231	PPARG
P62937	PPIA
P30405	PPIF
Q9Y3C6	PPIL1
Q9H2H8	PPIL3
O15297	PPM1D
Q8WUF5	PPP1R13L
O14990	PPP1R2C
Q96SB3	PPP1R9B
P53041	PPP5C
D3DTV9	PRAC2
Q96NZ9	PRAP1
O43663	PRC1
O75626	PRDM1
Q9H4Q3	PRDM13
Q13029	PRDM2
Q06830	PRDX1
P32119	PRDX2
P30044	PRDX5
P48147	PREP
Q5JRX3	PITRM1
Q13131	PRKAA1
P54646	PRKAA2
Q9Y478	PRKAB1
P22612	PRKACG
P05771	PRKCB
Q05655	PRKCD
Q02156	PRKCE
P41743	PRKCI
Q9BZL6	PRKD2
P78527	PRKDC
Q13237	PRKG2
O60260	PRKN
P01236	PRL
P40424	PBX1
Q99873	PRMT1
O14744	PRMT5
F7VJQ1	PRNP
P04156	PRNP
Q9HC23	PROK2

O43490	PROM1
O75360	PROP1
Q92786	PROX1
Q8WWY3	PRPF31
Q96HE9	PRR11
Q7Z6L0	PRRT2
Q9GZN4	PRSS22
Q6UWB4	PRSS55
Q16651	PRSS8
P07602	PSAP
Q8IWL1	SFTPA2
Q8IWL2	SFTPA1
Q9NZJ7	MTCH1
O43653	PSCA
A5PKW4	PSD
Q9NYI0	PSD3
Q8NDX1	PSD4
P49768	PSEN1
P49810	PSEN2
O75475	PSIP1
P28070	PSMB4
P28062	PSMB8
O75832	PSMD10
O00233	PSMD9
O95456	PSMG1
P26599	PTBP1
Q9UKA9	PTBP2
Q13635	PTCH1
Q7RTS3	PTF1A
O14684	PTGES
P23219	PTGS1
P35354	PTGS2
P01270	PTH
P12272	PTHLH
Q05397	PTK2
Q14289	PTK2B
P21246	PTN
O75365	PTP4A3
Q15257	PTPA
P18031	PTPN1
Q06124	PTPN11
P17706	PTPN2
P26045	PTPN3

P43378	PTPN9
P18433	PTPRA
P08575	PTPRC
P10586	PTPRF
Q12913	PTPRJ
O14522	PTPRT
Q92729	PTPRU
P23471	PTPRZ1
O95997	PTTG1
P53801	PTTG1IP
O75364	PITX3
P26022	PTX3
P15151	PVR
P49023	PXN
Q9BRQ0	PYGO2
Q96PU8	QKI
Q16769	QPCT
P61026	RAB10
Q9NP72	RAB18
P62820	RAB1A
Q9UL25	RAB21
Q9ULC3	RAB23
P51159	RAB27A
O00194	RAB27B
P61019	RAB2A
P0DI83	RAB34
Q9BZG1	RAB34
P57729	RAB38
P20336	RAB3A
P20337	RAB3B
Q8N4Z0	RAB42
Q86YS6	RAB43
P20339	RAB5A
P20340	RAB6A
P61006	RAB8A
P63000	RAC1
P15153	RAC2
P60763	RAC3
P63244	RACK1
Q9NS91	RAD18
Q92878	RAD50
Q06609	RAD51
Q96B01	RAD51AP1

O15315	RAD51B
P43351	RAD52
P46100	ATRX
Q92698	RAD54L
P04049	RAF1
Q15311	RALBP1
P49792	RANBP2
O60518	RANBP6
P46060	RANGAP1
P61224	RAP1B
P10114	RAP2A
P61225	RAP2B
Q8WZA2	RAPGEF4
P10826	RARB
P20936	RASA1
Q9Y272	RASD1
Q13972	RASGRF1
Q8IV61	RASGRP3
Q9NS23	RASSF1
A6NK89	RASSF10
Q9H2L5	RASSF4
Q8WWW0	RASSF5
Q9HCJ3	RAVER2
P06400	RB1
Q15291	RBBP5
Q7Z6E9	RBBP6
Q08999	RBL2
Q96PK6	RBM14
Q96I25	RBM17
Q8IUH3	RBM45
Q6XE24	RBMS3
O95153	TSPOAP1
P09455	RBP1
Q06330	RBPJ
Q8IZV5	RDH10
P35241	RDX
O95980	RECK
O94761	RECQL4
P05451	REG1A
Q9BYZ8	REG4
Q04206	RELA
Q01201	RELB
P00797	REN

Q9BWE0	REPIN1
Q13127	REST
O60673	REV3L
Q8N1G1	REXO1
P22670	RFX1
Q9H4X1	RGCC
P49796	RGS3
P49798	RGS4
Q96CC6	RHBDF1
P61586	RHOA
Q9BWJ2	RHPN1-AS1
Q8IUC4	RHPN2
Q6R327	RICTOR
Q8WYP3	RIN2
O14730	RIOK3
Q9H6W3	RIOX1
Q13546	RIPK1
O43353	RIPK2
Q9Y572	RIPK3
Q96DM3	RMC1
Q96DB5	RMDN1
Q96LZ7	RMDN2
Q96TC7	RMDN3
P12724	RNASE3
O75792	RNASEH2A
Q5TBB1	RNASEH2B
P61587	RND3
Q9ULX5	RNF112
Q8WVD3	RNF138
Q9NV58	RNF19A
Q9H4P4	RNF41
Q99942	RNF5
Q9Y6N7	ROBO1
Q8WZ75	ROBO4
Q13464	ROCK1
O75116	ROCK2
P35398	RORA
Q92753	RORB
P08922	ROS1
P35244	RPA3
Q86UA6	RPAIN
P62913	RPL11
P49207	RPL34

Q9Y3U8	RPL36
O95059	RPP14
P62841	RPS15
P62753	RPS6
Q15418	RPS6KA1
P51812	RPS6KA3
P23443	RPS6KB1
P08865	RPSA
Q8N122	RPTOR
P23921	RRM1
P31350	RRM2
Q2MKA7	RSPO1
Q15404	RSU1
Q9NZ71	RTEL1
Q9NQC3	RTN4
Q9BZR6	RTN4R
Q01196	RUNX1
Q13950	RUNX2
Q13761	RUNX3
Q9Y3V2	RWDD3
P34925	RYK
Q15413	RYR3
P23297	S100A1
P31949	S100A11
Q99584	S100A13
Q96FQ6	S100A16
P26447	S100A4
P06702	S100A9
P04271	S100B
O95136	S1PR2
P35542	SAA4
Q9UBE0	SAE1
Q9NXZ1	SAGE1
Q9UJQ4	SALL4
Q5K651	SAMD9
Q9NSI8	SAMSN1
Q9UHR5	SAP30BP
O94885	SASH1
Q01826	SATB1
Q8N9R8	SCAI
O00767	SCD
Q9UQD0	SCN8A
P51172	SCNN1D

O43819	SCO2
Q9NQ36	SCUBE2
Q96KG9	SCYL1
P18827	SDC1
P34741	SDC2
O00560	SDCBP
Q9NX18	SDHAF2
Q99643	SDHC
O14521	SDHD
O94855	SEC24D
Q9UBV2	SEL1L
Q14563	SEMA3A
Q13214	SEMA3B
Q99985	SEMA3C
Q9NS98	SEMA3G
Q9NPR2	SEMA4B
Q13591	SEMA5A
Q9H2E6	SEMA6A
O75326	SEMA7A
Q15019	SEPTIN2
Q16181	SEPTIN7
Q8NC51	SERBP1
P01011	SERPINA3
P30740	SERPINB1
P05120	SERPINB2
P05121	SERPINE1
P36955	SERPINF1
P05155	SERPING1
P50454	SERPINH1
P58004	SESN2
Q9BYW2	SETD2
Q9Y3B4	SF3B6
Q8N474	SFRP1
Q96HF1	SFRP2
Q6FHJ7	SFRP4
O00141	SGK1
Q86VZ5	SGMS1
Q8NHU3	SGMS2
Q96HU1	SGSM3
Q99961	SH3GL1
Q99962	SH3GL2
Q99963	SH3GL3
Q9Y371	SH3GLB1

Q5TCZ1	SH3PXD2A
Q9UPX8	SHANK2
P04278	SHBG
Q92529	SHC3
Q8NEM2	SHCBP1
Q15465	SHH
P34897	SHMT2
O60902	SHOX2
Q149N8	SHPRH
Q8IUQ4	SIAH1
Q9Y286	SIGLEC7
Q9H173	SIL1
Q14190	SIM2
Q96EB6	SIRT1
Q8IXJ6	SIRT2
Q9NTG7	SIRT3
Q8N6T7	SIRT6
Q9NRC8	SIRT7
Q15475	SIX1
O95343	SIX3
Q96BD8	SKA1
Q8WVK7	SKA2
P12755	SKI
Q13309	SKP2
Q13291	SLAMF1
Q9P0V8	SLAMF8
P55011	SLC12A2
Q9BXP2	SLC12A9
O15427	SLC16A3
O15374	SLC16A4
O60669	SLC16A7
O95907	SLC16A8
Q9P2U7	SLC17A7
P43004	SLC1A2
P43003	SLC1A3
Q8WUG5	SLC22A17
Q96BI1	SLC22A18
O75751	SLC22A3
Q6NUK1	SLC25A24
Q00325	SLC25A3
P40879	SLC26A3
O14975	SLC27A2
Q5K4L6	SLC27A3

Q99808	SLC29A1
P11166	SLC2A1
P11169	SLC2A3
Q9NR83	SLC2A4RG
P22732	SLC2A5
Q6PML9	SLC30A9
O95436	SLC34A2
P78382	SLC35A1
Q9Y2D2	SLC35A3
O43826	SLC37A4
Q99624	SLC38A3
Q8N1S5	SLC39A11
Q6P5W5	SLC39A4
Q8WWI5	SLC44A1
Q96NT5	SLC46A1
Q9BRV3	SLC50A1
Q9HAB3	SLC52A2
Q9NQ40	SLC52A3
Q92911	SLC5A5
Q8N695	SLC5A8
Q01959	SLC6A3
Q9UPY5	SLC7A11
Q01650	SLC7A5
Q9UM01	SLC7A7
Q9UPR5	SLC8A2
P19634	SLC9A1
O14745	SLC9A3R1
Q14940	SLC9A5
Q8IVB4	SLC9A9
Q86UG4	SLCO6A1
Q08AF3	SLFN5
Q6IEE8	SLFN12L
O75093	SLIT1
O94813	SLIT2
Q9H2G2	SLK
Q15797	SMAD1
Q15796	SMAD2
P84022	SMAD3
Q13485	SMAD4
O43541	SMAD6
O15105	SMAD7
P28370	SMARCA1
P51531	SMARCA2

P51532	SMARCA4
O60264	SMARCA5
Q12824	SMARCB1
Q14683	SMC1A
O95347	SMC2
Q9NTJ3	SMC4
Q16637	SMN1
Q9H4F8	SMOC1
P17405	SMPD1
Q53HV7	SMUG1
Q9HCE7	SMURF1
Q9H7B4	SMYD3
O95863	SNAI1
O43623	SNAI2
O60641	SNAP91
P37840	SNCA
Q7KZF4	SND1
Q9BXW3	SNHG12
P08621	SNRNP70
P35610	SOAT1
O14543	SOCS3
P00441	SOD1
P04179	SOD2
P08294	SOD3
Q99523	SORT1
Q07889	SOS1
O00570	SOX1
P56693	SOX10
Q9UN79	SOX13
P48431	SOX2
Q9Y651	SOX21
Q06945	SOX4
P35711	SOX5
P35712	SOX6
Q9BT81	SOX7
P48436	SOX9
P08047	SP1
Q8N907	DAND5
Q02447	SP3
A0A1B0GVQ0	SPAAR
P09486	SPARC
Q14515	SPARCL1
Q5MJ70	SPDYA

Q9NYA1	SPHK1
Q9NRA0	SPHK2
Q2M3C7	SPHKAP
P17947	SPI1
O43291	SPINT2
Q6ZMY3	SPOCD1
Q08629	SPOCK1
Q92563	SPOCK2
O43791	SPOP
P10451	SPP1
Q9BX95	SGPP1
O43609	SPRY1
O43597	SPRY2
Q9C004	SPRY4
O15269	SPTLC1
Q9BXG8	SPZ1
Q13501	SQSTM1
P12931	SRC
P18405	SRD5A1
Q12772	SREBF2
P10124	SRGN
P30626	SRI
Q96SB4	SRPK1
Q9Y5M8	SRPRB
Q9UQ35	SRRM2
Q07955	SRSF1
P84103	SRSF3
Q08945	SSRP1
P61278	SST
P30874	SSTR2
P31391	SSTR4
P50502	ST13
Q9Y5Y6	ST14
P15907	ST6GAL1
Q9BVH7	ST6GALNAC5
Q92185	ST8SIA1
Q8N3U4	STAG2
P25092	GUCY2C
P49675	STAR
Q9Y3M8	STARD13
P42224	STAT1
P52630	STAT2
P40763	STAT3

P42229	STAT5A
P51692	STAT5B
P42226	STAT6
P52823	STC1
Q658P3	STEAP3
Q13586	STIM1
P31948	STIP1
Q15831	STK11
Q9UEE5	STK17A
O00506	STK25
Q9Y2H1	STK38L
Q13043	STK4
P16949	STMN1
Q9NZ72	STMN3
Q9H668	STN1
Q9Y6Q2	STON1
Q9UJZ1	STOML2
Q8N801	STPG4
Q6J9G0	STYK1
Q9Y6J8	STYXL1
P53999	SUB1
P53597	SUCLG1
Q8IWU5	SULF2
P50225	SULT1A1
Q06520	SULT2A1
P63165	SUMO1
O43463	SUV39H1
Q9H5I1	SUV39H2
Q15022	SUZ12
Q7L0J3	SV2A
Q8NHG7	SVIP
Q9NX95	SYBU
O95926	SYF2
O15061	SYNM
P08247	SYP
P21579	SYT1
Q8NB59	SYT14
O43581	SYT7
P20366	TAC1
Q86UU9	TAC4
Q9Y6A5	TACC3
P25103	TACR1
P09758	TACSTD2

P37802	TAGLN2
P17542	TAL1
Q9H2K8	TAOK3
Q03518	TAP1
P17735	TAT
Q16635	TAZ
Q6ZT07	TBC1D9
O43435	TBX1
Q99081	TCF12
P15923	TCF3
Q9HCS4	TCF7L1
P15884	TCF4
Q9NQB0	TCF7L2
Q9UL49	TCFL5
Q2MV58	TCTN1
P28347	TEAD1
Q02763	TEK
Q5XG87	TENT4A
Q96IP4	TENT5A
P60484	PTEN
Q99973	TEP1
P54274	TERF1
O14746	TERT
Q8NFU7	TET1
Q6N021	TET2
O43151	TET3
Q8IYF3	TEX11
P02787	TF
Q00059	TFAM
P05549	TFAP2A
P19484	TFEB
Q07654	TFF3
P48307	TFPI2
P02786	TFRC
P01135	TGFA
P01137	TGFB1
P61812	TGFB2
Q15582	TGFBI
P37173	TGFBR2
Q9GZN2	TGIF2
P21980	TGM2
P07996	THBS1
P35442	THBS2

Q5T1C6	THEM4
P04216	THY1
Q86XR7	TICAM2
Q9NQ88	TIGAR
Q9UNS1	TIMELESS
O60220	TIMM8A
P01033	TIMP1
P16035	TIMP2
P35625	TIMP3
P29401	TKT
Q16832	DDR2
P51854	TKTL1
Q9UKI8	TLK1
O60603	TLR2
O00206	TLR4
Q9NYK1	TLR7
Q9NR96	TLR9
O76062	TM7SF2
Q9Y3A6	TMED5
Q9Y3B3	TMED7
Q8TBQ9	TMEM167A
Q96B42	TMEM18
Q9NWC5	TMEM45A
Q9BXS4	TMEM59
Q6P5X7	TMEM71
Q5BJF2	TMEM97
Q9NZQ9	TMOD4
P57727	TMPRSS3
Q9NRS4	TMPRSS4
P62328	TMSB4X
P24821	TNC
P01375	TNF
Q03169	TNFAIP2
Q6P589	TNFAIP8L2
O00220	TNFRSF10A
O14763	TNFRSF10B
O14798	TNFRSF10C
Q9UBN6	TNFRSF10D
Q9Y6Q6	TNFRSF11A
O00300	TNFRSF11B
Q9NP84	TNFRSF12A
O14836	TNFRSF13B
Q9NS68	TNFRSF19

P20333	TNFRSF1B
P43489	TNFRSF4
O95407	TNFRSF6B
Q07011	TNFRSF9
P50591	TNFSF10
O43508	TNFSF12
O95150	TNFSF15
P23510	TNFSF4
P32971	TNFSF8
Q15025	TNIP1
Q07912	TNK2
Q9H2S6	TNMD
Q9Y4F4	TOGARAM1
P11388	TOP2A
O94842	TOX4
P04637	TP53
Q12888	TP53BP1
Q96A56	TP53INP1
O15350	TP73
Q9ULQ1	TPCN1
P55327	TPD52
O43399	TPD52L2
P60174	TPI1
P09493	TPM1
P06753	TPM3
O94811	TPPP
Q9BW30	TPPP3
P13693	TPT1
P0DSE1	TRA
P0DTU3	TRA
Q13595	TRA2A
P62995	TRA2B
A6NFA1	TRABD2B
Q15628	TRADD
Q13077	TRAF1
Q12933	TRAF2
O43734	TRAF3IP2
O00463	TRAF5
Q9Y4K3	TRAF6
Q15629	TRAM1
Q12931	TRAP1
Q58FF3	HSP90B2P
Q6PIZ9	TRAT1

O43280	TREH
Q9NZC2	TREM2
Q9NSU2	TREX1
Q96RU8	TRIB1
Q92519	TRIB2
Q96F44	TRIM11
Q14142	TRIM14
O15164	TRIM24
P14373	TRIM27
Q13263	TRIM28
Q14134	TRIM29
O75382	TRIM3
Q9BZY9	TRIM31
O94972	TRIM37
Q96DX7	TRIM44
Q9H8W5	TRIM45
Q8IWR1	TRIM59
Q9BZR9	TRIM8
Q9H2D6	TRIOBP
P48995	TRPC1
Q9UL62	TRPC5
Q9Y210	TRPC6
Q96QT4	TRPM7
Q7Z2W7	TRPM8
Q8NER1	TRPV1
Q9Y5S1	TRPV2
Q9HBA0	TRPV4
Q96A04	TSACC
P49815	TSC2
Q63HK5	TSHZ3
Q12999	TSPAN31
P19075	TSPAN8
B1AH88	TSPO
P30536	TSPO
Q86VY4	TSPYL5
Q6IQ55	TTBK2
O95801	TTC4
P33981	TTK
P02766	TTR
Q9H313	TTYH1
Q13509	TUBB3
O75896	TUSC2
Q13454	TUSC3

Q15672	TWIST1
P10599	TXN
Q9H3M7	TXNIP
Q16881	TXNRD1
P19971	TYMP
P04818	TYMS
Q01081	U2AF1
Q5T6F2	UBAP2
Q14157	UBAP2L
O15205	UBD
O00762	UBE2C
P63279	UBE2I
P61088	UBE2N
Q16763	UBE2S
Q15386	UBE3C
Q04323	UBXN1
P09936	UCHL1
P55089	UCN
Q969E3	UCN3
Q16851	UGP2
Q16880	UGT8
Q96T88	UHRF1
Q96PU4	UHRF2
O75385	ULK1
Q8IYT8	ULK2
Q6ZN44	UNC5A
Q92900	UPF1
Q96BW1	UPRT
P22695	UQCRC2
P47985	UQCRFS1
Q8TCY9	URGCP
P22415	USF1
O94782	USP1
Q9Y4E8	USP15
Q6R6M4	USP17L2
Q0WX57	USP17L24
Q9UPT9	USP22
Q53GS9	USP39
Q9H0E7	USP44
Q86UV5	USP48
Q93009	USP7
Q93008	USP9X
P46939	UTRN

P51809	VAMP7
Q9BV40	VAMP8
Q9ULK5	VANGL2
Q6EMK4	VASN
P50552	VASP
P54219	SLC18A1
Q99536	VAT1
P19320	VCAM1
P13611	VCAN
P18206	VCL
P45880	VDAC2
P11473	VDR
P15692	VEGFA
P49767	VEGFC
O43915	VEGFD
P40337	VHL
P08670	VIM
P01282	VIP
Q8NEZ2	VPS37A
Q9UID3	VPS51
Q15906	VPS72
Q99986	VRK1
Q9Y279	VSIG4
P62760	VSNL1
Q96AJ9	VTI1A
P04004	VTN
P04275	VWF
Q9Y6W5	WASF2
O00401	WASL
Q969T9	WBP2
O75083	WDR1
Q8WWQ0	PHIP
Q9BZH6	WDR11
P30291	WEE1
Q9Y5W5	WIF1
Q5T9L3	WLS
Q9H4A3	WNK1
Q9Y3S1	WNK2
P04628	WNT1
O96014	WNT11
P09544	WNT2
Q93097	WNT2B
P56704	WNT3A

P41221	WNT5A
O00755	WNT7A
P56706	WNT7B
Q14191	WRN
P19544	WT1
Q15007	WTAP
Q6AWC2	WWC2
Q9NZC7	WWOX
Q9H0M0	WWP1
O00308	WWP2
Q9GZV5	WWTR1
Q6GPH4	XAF1
P17861	XBP1
P47992	XCL1
P98170	XIAP
O14980	XPO1
P18887	XRCC1
O43542	XRCC3
Q13426	XRCC4
P13010	XRCC5
P12956	XRCC6
P46937	YAP1
P67809	YBX1
O95619	YEATS4
P25490	YY1
P43403	ZAP70
Q05516	ZBTB16
Q9Y2Y4	ZBTB32
Q86T24	ZBTB33
Q86UZ6	ZBTB46
Q9NUE0	ZDHHC18
Q8IYP9	ZDHHC23
Q9C0B5	ZDHHC5
P37275	ZEB1
O60315	ZEB2
P16415	ZNF823
P26651	ZFP36
Q07352	ZFP36L1
Q96MM3	ZFP42
Q8WW38	ZFPM2
P17010	ZFX
Q8N5A5	ZGPAT
Q9Y6X8	ZHX2

Q8N9L1	ZIC4
Q96T25	ZIC5
O75800	ZMYND10
P52747	ZNF143
O75362	ZNF217
O43296	ZNF264
Q9Y2X9	ZNF281
Q8WUU4	ZNF296
Q5BKZ1	ZNF326
Q2M1K9	ZNF423
Q9C0F3	ZNF436
Q8IZM8	ZNF654
O60232	ZNRD2
O95218	ZRANB2
Q15942	ZYX
Q8N0V4	LGI2
Q92574	TSC1
Q7Z699	SPRED1
B2CW77	KLLN
P09104	ENO2
P13591	NCAM1
Q9HCJ1	ANKH
Q6ZV60	LINC00173
Q15283	RASA2
C9JLR9	C11orf95
Q8IXJ9	ASXL1
P01920	HLA-DQB1
Q9Y570	PPME1
P12643	BMP2
Q13541	EIF4EBP1
Q5TEC6	H3-2
P06401	PGR
Q9H3R0	KDM4C
Q9BVC4	MLST8
P21912	SDHB
Q07890	SOS2
Q10571	MN1
O43300	LRRTM2
O75325	LRRN2
P53355	DAPK1
P07949	RET
P22309	UGT1A1
P43355	MAGEA1

Q09472	EP300
Q9UMX1	SUFU
Q15382	RHEB
Q8TB45	DEPTOR
P85299	PRR5
O15530	PDPK1
P01730	CD4
Q14678	KANK1
P20248	CCNA2
Q969G3	SMARCE1
O15550	KDM6A
P23515	OMG
O60235	TMPRSS11D
Q71DI3	H3C15
Q92793	CREBBP
P00519	ABL1
P00374	DHFR
P55055	NR1H2
Q9Y2J2	EPB41L3
P01148	GNRH1
O75410	TACC1
O15355	PPM1G
Q9Y258	CCL26
P32297	CHRNA3
Q9UQ13	SHOC2
Q9P0N9	TBC1D7
A8K2U0	A2ML1
Q9Y383	LUC7L2
Q8NFP7	NUDT10
Q9BW91	NUDT9
Q9HBV1	POPDC3
Q9H853	TUBA4B
Q6ZU15	SEPTIN14
A6NCK2	TRIM43B
O75581	LRP6
P01008	SERPINC1
P31946	YWHAB
O15520	FGF10
P62424	RPL7A
P46597	ASMT
P99999	CYCS
Q15329	E2F5
Q4LE39	ARID4B

Q16254	E2F4
P08729	KRT7
Q96SD1	DCLRE1C
O43186	CRX
Q99835	SMO
Q9NWM0	SMOX
P36894	BMPR1A
Q7Z698	SPRED2
Q9UF02	CACNG5
Q2MJR0	SPRED3
Q9NZI7	UBP1
O43610	SPRY3
Q9NZP0	OR6C3
P22004	BMP6
P29590	PML
P53779	MAPK10
Q16611	BAK1
O75888	TNFSF13
P01210	PENK
Q15303	ERBB4
P62993	GRB2
Q9P0J6	MRPL36
P36402	TCF7
P62942	FKBP1A
P10645	CHGA
Q6P2D8	XRRA1
Q8TAK6	OLIG1
Q52LW3	ARHGAP29
O95096	NKX2-2
P20226	TBP
P16389	KCNA2
P10523	SAG
Q9Y6P5	SESN1
P15172	MYOD1
P49137	MAPKAPK2
O15169	AXIN1
Q00604	NDP
P51959	CCNG1
Q9UKX5	ITGA11
Q6U949	IGF2-AS
P29353	SHC1
Q16655	MLANA
Q86UD0	SAPCD2

O00459	PIK3R2
P60059	SEC61G
P98155	VLDLR
P54132	BLM
O00744	WNT10B
Q9H2X9	SLC12A5
P43657	LPAR6
P27694	RPA1
P20908	COL5A1
P21675	TAF1
P10768	ESD
Q9BQB4	SOST
Q99708	RBBP8
Q969F8	KISS1R
Q14186	TFDP1
Q14114	LRP8
Q92499	DDX1
Q09028	RBBP4
Q96MU8	KREMEN1
Q9NPI8	FANCF
Q9UHL9	GTF2IRD1
Q16666	IFI16
P17480	UBTF
Q8TDY2	RB1CC1
O75943	RAD17
O96020	CCNE2
P32519	ELF1
Q9H9Q4	NHEJ1
Q15726	KISS1
P28749	RBL1
Q9UBV4	WNT16
P17544	ATF7
Q8NB91	FANCB
Q00526	CDK3
O75140	DEPDC5
Q5VTD9	GFI1B
P35659	DEK
Q8IUD6	RNF135
O95239	KIF4A
P10745	RBP3
Q15554	TERF2
Q16576	RBBP7
Q99435	NELL2

P47736	RAP1GAP
Q15276	RABEP1
Q14188	TFDP2
Q5T4S7	UBR4
O75143	ATG13
P29374	ARID4A
P07814	EPRS1
O95678	KRT75
P22794	EVI2A
O75884	RBBP9
Q7Z7A3	CTU1
P34910	EVI2B
Q9BPY3	FAM118B
Q14696	MESD
Q9Y236	OSGIN2
A6NHX0	CASTOR2
P30872	SSTR1
P30793	GCH1
P53370	NUDT6
Q9BUL8	PDCD10
Q9Y6Y1	CAMTA1
Q15742	NAB2
P60022	DEFB1
Q12792	TWF1
P41161	ETV5
P45984	MAPK9
Q9GZP0	PDGFD
P23368	ME2
Q14980	NUMA1
O95750	FGF19
Q5T036	FAM120AOS
Q8NDV2	GPR26
P05129	PRKCG
Q96C34	RUNDC1
O95373	IPO7
Q14147	DHX34
Q86U86	PBRM1
P55210	CASP7
P16422	EPCAM
Q8NCR3	MFI
Q92466	DDB2
P09543	CNP
P69892	HBG2

P51668	UBE2D1
Q9H3S1	SEMA4A
P60866	RPS20
Q9Y6X0	SETBP1
Q16658	FSCN1
Q9Y2M0	FAN1
Q8N1L9	BATF2
P49763	PGF
P08195	SLC3A2
Q8WXG9	ADGRV1
O95257	GADD45G
O14964	HGS
Q13283	G3BP1
Q9UQM7	CAMK2A
P21583	KITLG
P11387	TOP1
Q9UHF7	TRPS1
Q9NZR2	LRP1B
Q6V1X1	DPP8
Q6S5L8	SHC4
Q8IX29	FBXO16
Q86V59	PNMA8A
P03971	AMH
P14174	MIF
P08684	CYP3A4
P41222	PTGDS
Q9Y2J4	AMOTL2
P05141	SLC25A5
Q01082	SPTBN1
Q6Q0C0	TRAF7
Q3BBV0	NBPF1
P98077	SHC2
O00212	RHOD
Q7Z2Z2	EFL1
P10644	PRKAR1A
P27448	MARK3
Q99062	CSF3R
Q14896	MYBPC3
P30041	PRDX6
P11171	EPB41
P50440	GATM
P15509	CSF2RA
P32754	HPD

P32745	SSTR3
Q05209	PTPN12
O75496	GMNN
P33260	CYP2C18
Q10567	AP1B1
P56377	AP1S2
O15232	MATN3
Q02928	CYP4A11
Q9H6I2	SOX17
O75928	PIAS2
O95271	TNKS
Q08477	CYP4F3
Q8N157	AHI1
Q9H1V8	SLC6A17
P35346	SSTR5
Q15561	TEAD4
P06126	CD1A
Q9Y3M2	CBY1
Q4VCS5	AMOT
Q96RE7	NACC1
P41238	APOBEC1
Q4AC94	C2CD3
Q5SZK8	FREM2
Q9UI47	CTNNA3
P09067	HOXB5
Q9BYD1	MRPL13
Q68CZ1	RPGRIP1L
Q02543	RPL18A
Q9UJ71	CD207
Q9HB15	KCNK12
Q2M1P5	KIF7
P29016	CD1B
P15812	CD1E
P31267	HOXA6
Q9Y399	MRPS2
Q5TAQ9	DCAF8
O75177	SS18L1
P52815	MRPL12
Q9NSI6	BRWD1
Q6ZRV2	FAM83H
Q8IY63	AMOTL1
Q9NQ79	CRTAC1
Q13938	CAPS

Q9ULU8	CADPS
Q8TD26	CHD6
P82930	MRPS34
O15263	DEFB4A
Q9NRA8	EIF4ENIF1
O75631	UPK3A
Q86YS3	RAB11FIP4
Q9H7D0	DOCK5
Q9NYH9	UTP6
Q9BYB4	GNB1L
Q9UKT5	FBXO4
Q9NPF8	ADAP2
Q5TCH4	CYP4A22
Q16540	MRPL23
Q8TBK2	SETD6
Q9GZN6	SLC6A16
O75564	JRK
Q8IYT4	KATNAL2
Q14135	VGLL4
Q7Z739	YTHDF3
Q13495	MAMLD1
Q8TEB9	RHBDD1
P0DPB3	SCHIP1
P83881	RPL36A
Q8TER0	SNED1
Q2KHM9	KIAA0753
Q8TAV4	STOML3
Q9BQJ4	TMEM47
Q8N3Z3	GTPBP8
Q9C0E4	GRIP2
Q9BV90	SNRNP25
Q9NQV6	PRDM10
O75157	TSC22D2
Q9BT92	TCHP
Q96QE3	ATAD5
Q96QE4	LRRC37B
Q63HQ0	AP1AR
Q8WW12	PCNP
Q96QE5	TEFM
Q99990	VGLL1
Q5TID7	CCDC181
Q9NQ92	COPRS
Q5T5A4	C1orf194

P0CG34	TMSB15A
Q8N268	C20orf197
P60331	KRTAP10-1
Q9Y693	LHFPL6
Q8IYF1	ELOA2
Q8N6C7	MIR7-3HG
Q6ZV56	C22orf34
Q6ZS62	COLCA1
Q17RA5	C21orf62-AS1
Q8NG57	ELOA3P
A6NH13	DNAJC9-AS1
Q3SY89	ELOA3BP
A0A1B0GTZ2	CCDC196
Q9Y6Z5	AFDN-DT
Q6ZST2	COL25A1-DT
O75427	LRCH4
Q6UXK5	LRRN1
A8MW92	PHF20L1
Q15759	MAPK11
Q16566	CAMK4
Q14012	CAMK1
P08700	IL3
Q33E94	RFX4
Q92915	FGF14
P78348	ASIC1
P55789	GFER
P16671	CD36
P13164	IFITM1
Q9NZP8	C1RL
P34972	CNR2
O14638	ENPP3
Q96S42	NODAL
P49765	VEGFB
P12034	FGF5
Q9NRA1	PDGFC
P51828	ADCY7
O43521	BCL2L11
Q8WWG1	NRG4
Q06416	POU5F1B
Q9HCB6	SPON1
P16581	SELE
P09917	ALOX5
Q99727	TIMP4

P43005	SLC1A1
Q15669	RHOH
P16885	PLCG2
Q13557	CAMK2D
P53778	MAPK12
O15264	MAPK13
P55075	FGF8
O60258	FGF17
Q9GZV9	FGF23
O43320	FGF16
Q8IU85	CAMK1D
Q96NX5	CAMK1G
O76093	FGF18
Q9NP95	FGF20
Q9NSA1	FGF21
P10767	FGF6
O75293	GADD45B
Q9HCT0	FGF22
Q9NZT1	CALML5
Q96GE6	CALML4
Q8TD86	CALML6
Q8WXW3	PIBF1
Q8N1I0	DOCK4
Q6NV75	GPR153
Q9UL01	DSE
P43686	PSMC4
Q99466	NOTCH4
Q9Y6Q9	NCOA3
P14138	EDN3
P10600	TGFB3
P40967	PMEL
P19793	RXRA
Q14766	LTBP1
P13385	TDGF1
Q8WTR2	DUSP19
P52333	JAK3
O60234	GMFG
P17252	PRKCA
P17612	PRKACA
O95467	GNAS
P63092	GNAS
P84996	GNAS
Q5JWF2	GNAS

P05107	ITGB2
P62745	RHOB
P40261	NNMT
Q9UIW2	PLXNA1
P78310	CXADR
P28223	HTR2A
O95342	ABCB11
P98177	FOXO4
P55957	BID
P48745	CCN3
Q6AI39	BICRAL
P20800	EDN2
Q99707	MTR
P43119	PTGIR
P01563	IFNA2
P63096	GNAI1
P10301	RRAS
O95399	UTS2
P68363	TUBA1B
Q92887	ABCC2
P46098	HTR3A
P35716	SOX11
O15554	KCNN4
P36952	SERPINB5
O75376	NCOR1
P68400	CSNK2A1
P24666	ACP1
P14416	DRD2
P01374	LTA
Q96KQ7	EHMT2
P23229	ITGA6
Q6ZN18	AEBP2
P49757	NUMB
P11717	IGF2R
P22303	ACHE
P47989	XDH
Q12857	NFIA
P08034	GJB1
P60983	GMFB
P21453	S1PR1
Q06203	PPAT
P10276	RARA
P28827	PTPRM

P13598	ICAM2
Q9NYB0	TERF2IP
Q9H6U6	BCAS3
Q9NP60	IL1RAPL2
P25024	CXCR1
P42262	GRIA2
P16050	ALOX15
P52757	CHN2
Q96H15	TIMD4
Q9UJM3	ERRFI1
P32942	ICAM3
P29474	NOS3
P60323	NANOS3
P00491	PNP
P01298	PPY
P29597	TYK2
Q15077	P2RY6
P08588	ADRB1
Q5VTR2	RNF20
Q9UPP2	IQSEC3
O94864	SUPT7L
Q99542	MMP19
P24593	IGFBP5
O94760	DDAH1
P18847	ATF3
Q9H492	MAP1LC3A
O15031	PLXNB2
P01286	GHRH
P23258	TUBG1
Q13609	DNASE1L3
Q9ULV4	CORO1C
P08519	LPA
P08571	CD14
Q08828	ADCY1
P30550	GRPR
Q96PG8	BBC3
Q9BXH1	BBC3
P10636	MAPT
Q96RQ9	IL4I1
P20941	PDC
Q92851	CASP10
P05109	S100A8
P21815	IBSP

P12524	MYCL
Q92521	PIGB
Q16602	CALCRL
P48740	MASP1
P18054	ALOX12
Q9H1Y0	ATG5
P21926	CD9
Q13573	SNW1
Q9BT40	INPP5K
O94817	ATG12
Q01638	IL1RL1
P20160	AZU1
P56211	ARPP19
Q3V6T2	CCDC88A
Q9UMX3	BOK
P55286	CDH8
Q8IZJ6	TDH
P21580	TNFAIP3
Q03393	PTS
Q01518	CAP1
Q13114	TRAF3
Q9C010	PKIB
Q96ST3	SIN3A
Q29980	MICB
Q9UBL3	ASH2L
O95786	DDX58
O15527	OGG1
P80098	CCL7
P41273	TNFSF9
P43357	MAGEA3
P10075	GLI4
Q13976	PRKG1
Q06710	PAX8
Q13490	BIRC2
P20292	ALOX5AP
Q8WUM4	PDCD6IP
O95983	MBD3
Q96LC9	BMF
O15197	EPHB6
P17275	JUNB
P11498	PC
Q99496	RNF2
Q9P0K8	FOXJ2

P62070	RRAS2
P17301	ITGA2
P34130	NTF4
P56199	ITGA1
P32239	CCKBR
Q04756	HGFAC
P67775	PPP2CA
P0DP91	ERCC6
Q03468	ERCC6
O43543	XRCC2
Q11206	ST3GAL4
P62333	PSMC6
Q9UNE7	STUB1
P07492	GRP
Q3ZCW2	LGALSL
O95819	MAP4K4
P60510	PPP4C
Q01831	XPC
Q8N4N8	KIF2B
O14782	KIF3C
O75037	KIF21B
O00238	BMPR1B
P36776	LONP1
P07093	SERPINE2
Q00975	CACNA1B
Q15147	PLCB4
P08397	HMBS
O15244	SLC22A2
Q9UBY5	LPAR3
Q53FA7	TP53I3
P0C1Z6	TFPT
Q13797	ITGA9
Q05586	GRIN1
O95832	CLDN1
P41594	GRM5
P35462	DRD3
P08651	NFIC
Q8NAT1	POMGNT2
Q9Y618	NCOR2
O60825	PFKFB2
Q13683	ITGA7
P35372	OPRM1
Q16695	H3-4

P19256	CD58
Q92583	CCL17
P51677	CCR3
O60733	PLA2G6
Q9UBK2	PPARGC1A
Q8TDS5	OXER1
Q05513	PRKCZ
P52564	MAP2K6
P06241	FYN
P28838	LAP3
P22102	GART
Q9BUT1	BDH2
O15455	TLR3
P63104	YWHAZ
O14907	TAX1BP3
P31152	MAPK4
P15248	IL9
Q13118	KLF10
P22003	BMP5
P08134	RHOC
P84095	RHOG
P38570	ITGAE
P04899	GNAI2
Q13017	ARHGAP5
P10997	IAPP
Q9H3H5	DPAGT1
Q13614	MTMR2
Q9UMD9	COL17A1
Q9H270	VPS11
Q9BUR4	WRAP53
P13798	APEH
Q15027	ACAP1
P07316	CRYGB
Q9Y296	TRAPPC4
Q70HW3	SLC25A26
Q9H3Y6	SRMS
Q9H892	TTC12
Q9UHA3	RSL24D1
Q9NPC7	MYNN
O43264	ZW10
Q5VZB9	DMRTA1
Q9BY12	SCAPER
Q9NS86	LANCL2

Q5HYJ3	FAM76B
Q9BQA5	HINFP
Q9P225	DNAH2
P11844	CRYGA
Q9NQC8	IFT46
Q9BSH3	NICN1
Q9H400	LIME1
A6NLP5	TTC36
Q8N9A8	CNEP1R1
Q8N4S0	CCDC82
O75964	ATP5MG
Q8NDF8	TENT4B
C9JDP6	CLDN25
Q96EL1	INKA1
Q9BVV2	FNDC11
Q86UT8	CENATAC
P41597	CCR2
P53985	SLC16A1
Q9NZJ5	EIF2AK3
P36956	SREBF1
Q9HC58	SLC24A3
O75604	USP2
P32246	CCR1
P32969	RPL9
P28288	ABCD3
O75381	PEX14
P09683	SCT
O75173	ADAMTS4
P41231	P2RY2
O95747	OXSR1
P78325	ADAM8
Q5EG05	CARD16
P13987	CD59
P12757	SKIL
P51114	FXR1
Q16534	HLF
Q9UH65	SWAP70
P50391	NPY4R
P24723	PRKCH
Q04759	PRKCQ
O95622	ADCY5
Q86X55	CARM1
O60266	ADCY3

O43306	ADCY6
P22694	PRKACB
P28702	RXRB
O00443	PIK3C2A
Q13163	MAP2K5
Q9UBS0	RPS6KB2
O60503	ADCY9
P49789	FHIT
P11487	FGF3
O75747	PIK3C2G
Q8NFM4	ADCY4
Q9Y219	JAG2
P48443	RXRG
Q13323	BIK
Q9HC35	EML4
Q99527	GPER1
A0A087X1C5	CYP2D7
P10809	HSPD1
P10144	GZMB
Q15583	TGIF1
P21728	DRD1
P62877	RBX1
Q9H013	ADAM19
Q9GZT9	EGLN1
O00468	AGRN
P05198	EIF2S1
P07437	TUBB
P35680	HNF1B
P08572	COL4A2
P12109	COL6A1
Q15119	PDK2
O00487	PSMD14
O75094	SLIT3
Q96RU2	USP28
P52756	RBM5
P47710	CSN1S1
Q92922	SMARCC1
P43026	GDF5
Q99570	PIK3R4
P11215	ITGAM
P13497	BMP1
O14807	MRAS
Q8WYR1	PIK3R5

P20701	ITGAL
Q16659	MAPK6
P35070	BTC
P26010	ITGB7
Q6KF10	GDF6
Q9Y625	GPC6
P61328	FGF12
O00292	LEFTY2
P02775	PPBP
Q9UK05	GDF2
P12645	BMP3
P53708	ITGA8
P40225	THPO
Q14767	LTBP2
Q9NS15	LTBP3
P56975	NRG3
O75610	LEFTY1
Q8N2S1	LTBP4
O14511	NRG2
O60383	GDF9
O43903	GAS2
P01242	GH2
Q92914	FGF11
Q8NI99	ANGPTL6
Q9Y240	CLEC11A
O95995	GAS8
P20774	OGN
Q9NTN9	SEMA4G
O75578	ITGA10
O95393	BMP10
P01229	LHB
Q7Z5Y6	BMP8A
P55107	GDF10
P58294	PROK1
P52198	RND2
Q13349	ITGAD
O43555	GNRH2
O14495	PLPP3
Q3YBR2	TBRG1
P05089	ARG1
Q14654	KCNJ11
Q02880	TOP2B
P08637	FCGR3A

Q13574	DGKZ
P48454	PPP3CC
Q16787	LAMA3
Q15262	PTPRK
Q9UEY8	ADD3
Q9Y4R8	TELO2
Q15599	SLC9A3R2
O43312	MTSS1
Q9UPM6	LHX6
Q86YN6	PPARGC1B
Q99836	MYD88
Q08499	PDE4D
P08709	F7
P09525	ANXA4
O15245	SLC22A1
Q9BVP2	GNL3
Q13519	PNOC
Q92737	RASL10A
P29350	PTPN6
P37088	SCNN1A
P55064	AQP5
Q8WUI4	HDAC7
Q9UKV0	HDAC9
P48775	TDO2
Q16875	PFKFB3
Q8WUM9	SLC20A1
Q9BV57	ADI1
Q16690	DUSP5
P04844	RPN2
Q86WJ1	CHD1L
O76061	STC2
Q9NQG5	RPRD1B
O14893	GEMIN2
Q8N387	MUC15
Q96N19	GPR137
Q96AW1	VOPP1
Q92537	SUSD6
P0DMC3	APELA
P00492	HPRT1
P42330	AKR1C3
P19447	ERCC3
O60238	BNIP3L
Q9BRQ8	AIFM2

Q9BZM5	ULBP2
Q9UIK5	TMEFF2
O60241	ADGRB2
P60880	SNAP25
P62826	RAN
P36897	TGFBR1
P54762	EPHB1
Q16623	STX1A
Q9UBX5	FBLN5
Q14031	COL4A6
Q9NSC2	SALL1
O43157	PLXNB1
P53420	COL4A4
P29400	COL4A5
O60285	NUAK1
P06703	S100A6
P10915	HAPLN1
O75078	ADAM11
P10147	CCL3
Q96JB5	CDK5RAP3
Q9H2V7	SPNS1
Q9Y657	SPIN1
Q70Z35	PREX2
Q9P287	BCCIP
Q9GZV7	HAPLN2
Q9ULC4	MCTS1
Q67FW5	B3GNTL1
Q7Z7M8	B3GNT8
Q8N6R0	EEF1AKNMT
Q15714	TSC22D1
Q9NZJ0	DTL
P02790	HPX
Q12809	KCNH2
P61769	B2M
P49840	GSK3A
Q9UHW9	SLC12A6
P04839	CYBB
P19784	CSNK2A2
P10909	CLU
P28065	PSMB9
P60891	PRPS1
P35575	G6PC
P15502	ELN

P11908	PRPS2
P46663	BDKRB1
P51685	CCR8
Q96PY6	NEK1
Q92844	TANK
Q13330	MTA1
P13645	KRT10
P50219	MNX1
P34995	PTGER1
P51965	UBE2E1
Q96PM5	RCHY1
Q16585	SGCB
Q96FW1	OTUB1
Q96D96	HVCN1
Q14802	FXYD3
Q9BZ11	ADAM33
Q9Y547	HSPB11
Q6PJ69	TRIM65
Q8NCD3	HJURP
Q9NYS0	NKIRAS1
Q9NUM3	SLC39A9
Q8IYD2	KLHDC8A
Q9BYJ0	FGFBP2
Q7Z3Z3	PIWIL3
B1AL88	FAM155A
Q6MZN7	HCP5
P98172	EFNB1
P41146	OPRL1
P17535	JUND
Q9BZM6	ULBP1
P08949	NMB
Q14956	GPNMB
P05164	MPO
P35579	MYH9
P62136	PPP1CA
P60033	CD81
O60880	SH2D1A
Q93063	EXT2
P07195	LDHB
Q9NPC2	KCNK9
Q9Y275	TNFSF13B
P56159	GFRA1
P02765	AHSG

P43155	CRAT
Q02223	TNFRSF17
Q9UKE5	TNIK
Q9Y3E5	PTRH2
P27815	PDE4A
O60243	HS6ST1
P30048	PRDX3
Q9BUZ4	TRAF4
Q12923	PTPN13
P35590	TIE1
Q8TAA9	VANGL1
P61158	ACTR3
Q13952	NFYC
Q8TE12	LMX1A
Q01469	FABP5
Q8NFJ5	GPRC5A
Q9UBZ9	REV1
Q12967	RALGDS
P42830	CXCL5
Q96MM7	HS6ST2
Q9H1H9	KIF13A
Q5VV41	ARHGEF16
Q18PE1	DOK7
P47972	NPTX2
Q9NQT8	KIF13B
O60542	PSPN
Q9UKI2	CDC42EP3
Q9Y2G4	ANKRD6
Q99653	CHP1
Q9UHD1	CHORDC1
Q9HCM9	TRIM39
Q9BRK4	LZTS2
Q96T23	RSF1
Q8WW36	ZCCHC13
Q9ULE0	WWC3
A8MXQ7	IQANK1
P09172	DBH
P10827	THRA
P19883	FST
Q92952	KCNN1
O15111	CHUK
P43694	GATA4
Q04912	MST1R

Q15080	NCF4
P08174	CD55
P30519	HMOX2
Q07973	CYP24A1
P43034	PAFAH1B1
P36873	PPP1CC
P11234	RALB
P62495	ETF1
P45844	ABCG1
Q9Y5R2	MMP24
Q9NXR1	NDE1
Q9NPH3	IL1RAP
Q9Y4L1	HYOU1
Q96QK1	VPS35
Q8WU20	FRS2
Q06587	RING1
Q96PD4	IL17F
Q13596	SNX1
Q9Y266	NUDC
P11831	SRF
Q9Y4G8	RAPGEF2
Q96AY2	EME1
P50479	PDLIM4
P49862	KLK7
P22079	LPO
Q13112	CHAF1B
Q9H239	MMP28
Q9NPA2	MMP25
Q5T4F7	SFRP5
O60749	SNX2
P62280	RPS11
O60486	PLXNC1
O75436	VPS26A
Q01523	DEFA5
P50876	RNF144A
P35243	RCVRN
Q9P2N4	ADAMTS9
P62851	RPS25
P30408	TM4SF1
Q8IYD1	GSPT2
Q01664	TFAP4
Q9BX70	BTBD2
Q9UNH7	SNX6

Q9BYH8	NFKBIZ
O60907	TBL1X
Q15013	MAD2L1BP
Q9Y2B0	CNPY2
Q6RUI8	C19orf48
P00395	MT-CO1
Q9HBH7	BEX1
P01854	IGHE
Q495Z4	ASB16-AS1
Q7Z7D3	VTCN1
Q96QU1	PCDH15
Q9Y616	IRAK3
Q9H3D4	TP63
P01024	C3
P13498	CYBA
Q13258	PTGDR
Q15399	TLR1
Q14739	LBR
P09960	LTA4H
P01031	C5
P04183	TK1
P02771	AFP
P12259	F5
P06734	FCER2
P12318	FCGR2A
P09871	C1S
P02745	C1QA
P00751	CFB
Q99574	SERPINI1
P06870	KLK1
Q14765	STAT4
P08686	CYP21A2
P16109	SELP
P49908	SELENOP
P49257	LMAN1
P02746	C1QB
P02747	C1QC
O00187	MASP2
P06681	C2
P02652	APOA2
O75306	NDUFS2
Q14626	IL11RA
Q9Y239	NOD1

P32418	SLC8A1
P17066	HSPA6
Q9H0E2	TOLLIP
Q9Y2C9	TLR6
Q8IUC6	TICAM1
P02748	C9
P30273	FCER1G
P51671	CCL11
Q9UNN5	FAF1
O75342	ALOX12B
P21953	BCKDHB
P09110	ACAA1
P21730	C5AR1
P07358	C8B
Q9NNX6	CD209
P98082	DAB2
O15524	SOCS1
O14508	SOCS2
Q96RU7	TRIB3
P13671	C6
P10643	C7
Q9Y6Y9	LY96
P78357	CNTNAP1
P54821	PRRX1
P29508	SERPINB3
Q9Y5U5	TNFRSF18
Q14242	SELPLG
Q9Y5K2	KLK4
Q9BYJ1	ALOXE3
P98171	ARHGAP4
P36941	LTBR
O15296	ALOX15B
P17213	BPI
P07357	C8A
P07360	C8G
Q9UQC9	CLCA2
P45877	PPIC
O43187	IRAK2
Q9Y5Y4	PTGDR2
Q9H2R5	KLK15
P51460	INSL3
P51686	CCR9
P55899	FCGRT

P30043	BLVRB
Q96CA5	BIRC7
Q9UJU5	FOXD3
P04003	C4BPA
P20851	C4BPB
P40198	CEACAM3
A8K7I4	CLCA1
P25713	MT3
Q9H4E7	DEF6
Q92841	DDX17
P11684	SCGB1A1
Q9H4L4	SENP3
Q9BXR5	TLR10
P60852	ZP1
Q09327	MGAT3
Q9UMF0	ICAM5
Q0D2K0	NIPAL4
Q99467	CD180
Q9BYP7	WNK3
Q8TAP4	LMO3
P22362	CCL1
Q99616	CCL13
O75689	ADAP1
Q8TDM6	DLG5
Q8WVN6	SECTM1
Q9UNG2	TNFSF18
Q969D9	TSLP
P49842	STK19
P01732	CD8A
P21145	MAL
P58753	TIRAP
Q5QGZ9	CLEC12A
Q9BYE0	HES7
Q8TAX7	MUC7
Q01524	DEFA6
Q9BYW1	SLC2A11
O14544	SOCS6
Q8WXH5	SOCS4
Q6IBS0	TWF2
Q6W4X9	MUC6
P36508	ZNF76
O00175	CCL24
Q709F0	ACAD11

Q9UHP7	CLEC2D
Q9NPR9	GPR108
P59666	DEFA3
P12838	DEFA4
Q9P0M4	IL17C
Q14773	ICAM4
Q96P16	RPRD1A
O75717	WDHD1
Q674R7	ATG9B
Q9H6R0	DHX33
Q9UQP3	TNN
Q96AC6	KIFC2
O43281	EFS
O95104	SCAF4
Q5FYA8	ARSH
O95870	ABHD16A
Q8N690	DEFB119
Q969F2	NKD2
O95857	TSPAN13
Q8IY67	RAVER1
O95868	LY6G6D
Q5SQ64	LY6G6F
Q495B1	ANKDD1A
O60502	OGA
Q96DC7	TMCO6
O95388	CCN4
Q96PH6	DEFB118
Q7Z589	EMSY
Q5JRS4	OR10J3
Q9H1M4	DEFB127
Q9BYW3	DEFB126
P68366	TUBA4A
Q13275	SEMA3F
P24347	MMP11
P12814	ACTN1
P01009	SERPINA1
P19438	TNFRSF1A
P35408	PTGER4
P43116	PTGER2
P09668	CTSH
P28799	GRN
P14679	TYR
Q03181	PPARD

P49662	CASP4
P08754	GNAI3
P22392	NME2
O76082	SLC22A5
P17643	TYRP1
P62508	ESRRG
P80404	ABAT
Q02224	CENPE
Q99500	S1PR3
P54727	RAD23B
P56539	CAV3
Q13370	PDE3B
Q9H015	SLC22A4
Q9H7Z7	PTGES2
Q15185	PTGES3
P49454	CENPF
Q96NY9	MUS81
Q9Y620	RAD54B
P32321	DCTD
P30301	MIP
Q8WZ33	MAFIP
Q99797	MIPEP
O14735	CDIPT
Q9Y2J8	PADI2
P24863	CCNC
Q9UKL0	RCOR1
Q8IV63	VRK3
Q17RS7	GEN1
Q03111	MLLT1
Q9HCU0	CD248
Q9BZM4	ULBP3
Q9BPX3	NCAPG
Q8TD07	RAET1E
Q6NS38	ALKBH2
Q8TF62	ATP8B4
O75452	RDH16
Q9GZX5	ZNF350
O60831	PRAF2
Q9BZM3	GSX2
Q8WYN3	CSRNP3
P60896	SEM1
Q6ZVN7	SEM1
Q9H8L6	MMRN2

Q9NT22	EMILIN3
Q9UK76	JPT1
O60224	SSX4
P13639	EEF2
P22455	FGFR4
O95718	ESRRB
Q07864	POLE
P16871	IL7R
P37837	TALDO1
P00736	C1R
P36382	GJA5
Q01432	AMPD3
O14764	GABRD
P35367	HRH1
O43837	IDH3B
Q9HBG4	ATP6V0A4
P31641	SLC6A6
O00161	SNAP23
P30533	LRPAP1
P25105	PTAFR
Q16548	BCL2A1
Q99418	CYTH2
P50749	RASSF2
Q13561	DCTN2
P47872	SCTR
Q02535	ID3
P52888	THOP1
P02818	BGLAP
O15551	CLDN3
Q9Y2K7	KDM2A
Q92187	ST8SIA4
Q9HD36	BCL2L10
Q86W56	PARG
Q8IYM9	TRIM22
P24390	KDELR1
P38567	SPAM1
Q07687	DLX2
Q6NXG1	ESRP1
O95232	LUC7L3
O15218	GPR182
P41567	EIF1
Q15818	NPTX1
Q9BRU2	TCEAL7

P03923	MT-ND6
O95473	SYNGR4
P63302	SELENOW
Q99459	CDC5L
P11274	BCR
P48067	SLC6A9
P38646	HSPA9
P05187	ALPP
P19440	GGT1
P22736	NR4A1
P42684	ABL2
O00273	DFFA
Q9UG56	PISD
P46019	PHKA2
Q13191	CBLB
O15354	GPR37
P43007	SLC1A4
P20062	TCN2
Q05823	RNASEL
P07204	THBD
P32320	CDA
Q9Y2T4	PPP2R2C
Q9GZT4	SRR
Q01581	HMGCS1
Q9UF33	EPHA6
Q8N6G5	CSGALNACT2
Q96P70	IPO9
Q96S59	RANBP9
O60318	MCM3AP
O75508	CLDN11
O60906	SMPD2
O15056	SYNJ2
P18754	RCC1
Q8TDU6	GPBAR1
Q6B8I1	DUSP13
Q9UII6	DUSP13
Q68DV7	RNF43
Q6NUQ1	RINT1
Q9P0U1	TOMM7
Q8N271	PROM2
O96028	NSD2
Q8IZ40	RCOR2
Q96EF6	FBXO17

P79483	HLA-DRB3
P62258	YWHAE
P28329	CHAT
Q15853	USF2
P52803	EFNA5
Q9Y5Y7	LYVE1
Q9Y3D7	PAM16
O75807	PPP1R15A
P28222	HTR1B
P29373	CRABP2
P14543	NID1
P54136	RARS1
Q8TDW4	ST7L
P02452	COL1A1
P49279	SLC11A1
P28221	HTR1D
P05408	SCG5
Q15223	NECTIN1
P43405	SYK
Q9Y6L6	SLCO1B1
O14788	TNFSF11
O94956	SLCO2B1
Q93070	ART4
Q99704	DOK1
Q13308	PTK7
O95613	PCNT
Q96BD0	SLCO4A1
Q9NYB5	SLCO1C1
P46721	SLCO1A2
Q13085	ACACA
P15428	HPGD
P30613	PKLR
Q15758	SLC1A5
Q01995	TAGLN
P15036	ETS2
P51948	MNAT1
P07197	NEFM
P49447	CYB561
Q96B97	SH3KBP1
Q15836	VAMP3
Q9BQG0	MYBBP1A
Q9NZL4	HSPBP1
Q9H1A3	METTL9

P60602	ROMO1
O00634	NTN3
Q6NSW7	NANOGP8
Q07157	TJP1
Q9NS75	CYSLTR2
P0DMS8	ADORA3
P21796	VDAC1
Q13822	ENPP2
Q9Y6W8	ICOS
Q9UPG8	PLAGL2
Q9UBF1	MAGEC2
P25098	GRK2
P30153	PPP2R1A
P42702	LIFR
Q13510	ASAH1
P04080	CSTB
P63151	PPP2R2A
Q14738	PPP2R5D
P63027	VAMP2
Q9NR71	ASAH2
Q99684	GFI1
Q9NVI7	ATAD3A
O60925	PFDN1
Q9Y2H2	INPP5F
Q5T9A4	ATAD3B
Q5TB80	CEP162
P32929	CTH
Q96IQ7	VSIG2
P68133	ACTA1
Q13621	SLC12A1
P11279	LAMP1
P14207	FOLR2
Q9ULW0	TPX2
P22676	CALB2
P40313	CTRL
Q9UL45	BLOC1S6
P31943	HNRNPH1
Q8WVC0	LEO1
Q92576	PHF3
Q9BYN0	SRXN1
Q96DY2	IQCD
P30260	CDC27
P07101	TH

Q12906	ILF3
P23467	PTPRB
Q13740	ALCAM
Q8N6G6	ADAMTSL1
P07099	EPHX1
Q12882	DPYD
Q15139	PRKD1
Q9BY76	ANGPTL4
Q15078	CDK5R1
Q9P0X4	CACNA1I
O14497	ARID1A
Q8NFD5	ARID1B
Q8TAQ2	SMARCC2
Q15427	SF3B4
Q92925	SMARCD2
Q68CP9	ARID2
Q96GM5	SMARCD1
Q6STE5	SMARCD3
P62273	RPS29
Q15532	SS18
P78545	ELF3
Q9NP62	GCM1
P04217	A1BG
Q9H8M2	BRD9
Q4VC05	BCL7A
Q9BQE9	BCL7B
O75764	TCEA3
Q6UWM5	GLIPR1L1
Q8WXS4	TMEM37
Q9NVP4	DZANK1
P12107	COL11A1
P32238	CCKAR
P01160	NPPA
Q15149	PLEC
O60942	RNGTT
P13727	PRG2
Q6T4P5	PLPPR3
Q92626	PXDN
Q9BQ16	SPOCK3
Q07326	PIGF
Q13618	CUL3
Q9UGI8	TES
O00519	FAAH

Q7L5A8	FA2H
P27540	ARNT
P40937	RFC5
P31040	SDHA
P13051	UNG
Q13569	TDG
P08263	GSTA1
Q15654	TRIP6
P01903	HLA-DRA
P26717	KLRC2
Q6UXS9	CASP12
O43252	PAPSS1
P78559	MAP1A
P55072	VCP
O43570	CA12
P09238	MMP10
P23497	SP100
Q06643	LTB
Q9NYB9	ABI2
P53396	ACLY
P62979	RPS27A
P51582	P2RY4
Q86SH2	ZAR1
P06276	BCHE
P00390	GSR
Q9BYX4	IFIH1
Q92993	KAT5
P11229	CHRM1
Q9UBU2	DKK2
O60895	RAMP2
Q96EP1	CHFR
Q13075	NAIP
Q92904	DAZL
Q15020	SART3
O15304	SIVA1
Q8IVL0	NAV3
Q99683	MAP3K5
Q99519	NEU1
P16860	NPPB
P30825	SLC7A1
P07998	RNASE1
Q92896	GLG1
Q14623	IHH

P02795	MT2A
P23141	CES1
Q00973	B4GALNT1
Q92854	SEMA4D
P30939	HTR1F
Q9Y5K6	CD2AP
P63208	SKP1
P08962	CD63
Q9UH73	EBF1
P28676	GCA
O43290	SART1
P43358	MAGEA4
Q96LB2	MRGPRX1
Q8NBP7	PCSK9
Q14643	ITPR1
P10912	GHR
Q13813	SPTAN1
Q9UJU2	LEF1
O43526	KCNQ2
P05156	CFI
P07954	FH
P00488	F13A1
P20823	HNF1A
Q02127	DHODH
P21673	SAT1
Q9H2B4	SLC26A1
Q9H2H9	SLC38A1
O43525	KCNQ3
Q9Y6A1	POMT1
P32241	VIPR1
P23025	XPA
P36543	ATP6V1E1
Q14894	CRYM
O15287	FANCG
Q13162	PRDX4
Q15274	QPRT
Q9NS56	TOPORS
Q12846	STX4
P09210	GSTA2
Q9H4M9	EHD1
P27144	AK4
P46091	GPR1
Q9UQ80	PA2G4

Q9NZN3	EHD3
Q9NZN4	EHD2
Q9UNY4	TTF2
Q9H223	EHD4
O43809	NUDT21
O76050	NEURL1
Q9H299	SH3BGRL3
O60242	ADGRB3
O75506	HSBP1
Q02833	RASSF7
Q86X51	EZHIP
Q03431	PTH1R
P35270	SPR
P51178	PLCD1
Q13507	TRPC3
P33991	MCM4
Q99759	MAP3K3
P29275	ADORA2B
P00568	AK1
P09471	GNAO1
Q13422	IKZF1
Q15858	SCN9A
Q01970	PLCB3
P52732	KIF11
P08913	ADRA2A
P49711	CTCF
P11532	DMD
P11912	CD79A
Q99720	SIGMAR1
Q13651	IL10RA
P62714	PPP2CB
Q14542	SLC29A2
Q8WVQ1	CANT1
P35754	GLRX
P21399	ACO1
Q9C0B1	FTO
O43181	NDUFS4
Q08334	IL10RB
P18077	RPL35A
Q93034	CUL5
P78508	KCNJ10
P19086	GNAZ
Q03933	HSF2

P02549	SPTA1
Q16739	UGCG
P48200	IREB2
Q86XK2	FBXO11
Q9UKB1	FBXW11
Q99700	ATXN2
P14091	CTSE
Q9NR09	BIRC6
P84098	RPL19
Q99608	NDN
P46781	RPS9
Q92752	TNR
Q9Y496	KIF3A
Q9UKP6	UTS2R
Q9UBI6	GNG12
Q96PD2	DCBLD2
O60573	EIF4E2
P61604	HSPE1
Q13888	GTF2H2
Q9UQ49	NEU3
Q16348	SLC15A2
O95707	POP4
P0DMW2	NLRP2B
P63215	GNG3
Q9Y483	MTF2
Q9GZY8	MFF
Q01101	INSM1
Q96QF0	RAB3IP
O43303	CCP110
Q96HE7	ERO1A
Q96LT9	RNPC3
O60356	NUPR1
P56381	ATP5F1E
O60477	BRINP1
P58180	OR4D2
Q9BQY4	RHOXF2
P0CF75	EBLN1
A0A075B6S2	IGKV2D-29
P14778	IL1R1
P12111	COL6A3
O00213	APBB1
P22626	HNRNPA2B1
Q96AP0	ACD

Q15047	SETDB1
P23771	GATA3
P24001	IL32
Q15782	CHI3L2
P19957	PI3
P51170	SCNN1G
O75962	TRIO
P81172	HAMP
Q03519	TAP2
Q9UDX4	SEC14L3
Q9Y5J3	HEY1
O15533	TAPBP
Q9UKW4	VAV3
Q9NQW6	ANLN
Q8IZL9	CDK20
P53609	PGGT1B
Q96JB1	DNAH8
Q9NV64	TMEM39A
P50148	GNAQ
P47712	PLA2G4A
O14649	KCNK3
P31785	IL2RG
P45379	TNNT2
Q71U36	TUBA1A
P56817	BACE1
P08236	GUSB
P22894	MMP8
Q99697	PITX2
P13647	KRT5
P02533	KRT14
Q04917	YWHAH
P27348	YWHAQ
P41181	AQP2
P12277	CKB
P20810	CAST
Q16653	MOG
Q13639	HTR4
P05165	PCCA
Q9NVS9	PNPO
P32243	OTX2
Q14573	ITPR3
Q06278	AOX1
P25685	DNAJB1

Q14833	GRM4
P14859	POU2F1
P16298	PPP3CB
Q9Y6H5	SNCAIP
P62837	UBE2D2
P17600	SYN1
O14818	PSMA7
P04155	TFF1
Q86UL8	MAGI2
P50052	AGTR2
O76090	BEST1
P01225	FSHB
P20265	POU3F2
Q13424	SNTA1
Q16143	SNCB
Q16572	SLC18A3
Q9UMX0	UBQLN1
Q05707	COL14A1
P33316	DUT
O14974	PPP1R12A
P35475	IDUA
Q8WX93	PALLD
P28336	NMBR
O76070	SNCG
P62191	PSMC1
Q9NUD9	PIGV
O00142	TK2
P01266	TG
O75340	PDCD6
Q9H553	ALG2
Q01484	ANK2
P07451	CA3
P12829	MYL4
Q15049	MLC1
Q14872	MTF1
Q9Y4D1	DAAM1
P61916	NPC2
Q9Y5X1	SNX9
Q9Y2I1	NISCH
Q13835	PKP1
Q96QD8	SLC38A2
P62195	PSMC5
P17980	PSMC3

Q93096	PTP4A1
P29074	PTPN4
O14813	PHOX2A
P28072	PSMB6
Q8IWU6	SULF1
Q8WWN8	ARAP3
P56270	MAZ
P49802	RGS7
Q9UHL4	DPP7
Q86TI2	DPP9
P24387	CRHBP
P35998	PSMC2
Q92882	OSTF1
Q8TDQ7	GNPDA2
Q9H3P7	ACBD3
Q70SY1	CREB3L2
Q13505	MTX1
P05543	SERPINA7
Q8NBS9	TXNDC5
Q92777	SYN2
Q9UNK0	STX8
Q9HC07	TMEM165
Q9GZS3	WDR61
Q9Y468	L3MBTL1
Q8IYR6	TMEFF1
P53805	RCAN1
Q08554	DSC1
Q9BS86	ZPBP
Q9UHR4	BAIAP2L1
O94973	AP2A2
Q02410	APBA1
P57771	RGS8
Q9BPU6	DPYSL5
P57073	SOX8
Q96RF0	SNX18
Q9H3S5	PIGM
Q96CJ1	EAF2
Q4G0P3	HYDIN
O95273	CCNDBP1
Q16342	PDCD2
Q9NS69	TOMM22
Q13136	PPFIA1
Q5JY77	GPRASP1

Q8TCU3	SLC7A13
Q9HB20	PLEKHA3
Q86VD9	PIGZ
Q6EBC2	IL31
Q96HL8	SH3YL1
Q8N9U0	TC2N
Q6N075	MFSD5
Q9BRN9	TM2D3
L0R8F8	MIEF1
Q9NQG6	MIEF1
A2RRD8	ZNF320
Q9Y3D0	CIAO2B
A6NEK1	ARRDC5
Q9H714	RUBCNL
Q6NVV9	ADAM5
A4D0T7	SMIM30
Q9H0P7	AGPAT4-IT1
P17812	CTPS1
P23468	PTPRD
P0DJI8	SAA1
P24855	DNASE1
Q01538	MYT1
Q99640	PKMYT1
Q9UHA4	LAMTOR3
Q8IUK5	PLXDC1
P21964	COMT
P61764	STXBP1
Q92831	KAT2B
P35219	CA8
P50993	ATP1A2
P31994	FCGR2B
P22413	ENPP1
P15498	VAV1
Q02962	PAX2
Q9NP59	SLC40A1
A0A140G945	CRYAA2
P02489	CRYAA
Q07075	ENPEP
Q9GZV3	SLC5A7
Q9UP52	TFR2
O43516	WIPF1
P49407	ARRB1
Q12959	DLG1

P04440	HLA-DPB1
Q92956	TNFRSF14
O00629	KPNA4
P62253	UBE2G1
Q03060	CREM
O95665	NTSR2
O60896	RAMP3
P51784	USP11
Q9Y2M5	KLHL20
P35626	GRK3
Q6ZMG9	CERS6
Q08050	FOXM1
Q14168	MPP2
Q29RF7	PDS5A
P78395	PRAME
Q8IWT3	CUL9
Q6ZVN8	HJV
Q9P127	LUZP4
O43318	MAP3K7
Q12879	GRIN2A
P01019	AGT
O60566	BUB1B
P61244	MAX
P08172	CHRM2
P02458	COL2A1
P16471	PRLR
P12235	SLC25A4
Q01726	MC1R
P35221	CTNNA1
O43464	HTRA2
P49281	SLC11A2
P61077	UBE2D3
P32322	PYCR1
P52209	PGD
P20700	LMNB1
Q9UBM7	DHCR7
P08246	ELANE
P05177	CYP1A2
P25774	CTSS
P14222	PRF1
Q16512	PKN1
Q13885	TUBB2A
Q9UDY2	TJP2

P08238	HSP90AB1
P05413	FABP3
P49639	HOXA1
P25021	HRH2
P08697	SERPINF2
O75908	SOAT2
Q14994	NR1I3
P68371	TUBB4B
P23763	VAMP1
Q9P2W7	B3GAT1
P02788	LTF
Q9UQ84	EXO1
P49146	NPY2R
Q9UD71	PPP1R1B
Q92673	SORL1
Q9NYY3	PLK2
P48664	SLC1A6
Q11201	ST3GAL1
P22105	TNXB
P0CG48	UBC
O43593	HR
Q9UKL4	GJD2
Q92765	FRZB
P48382	RFX5
O95479	H6PD
Q8N465	D2HGDH
P32302	CXCR5
P62917	RPL8
O00592	PODXL
O00214	LGALS8
P46063	RECQL
Q9Y6F9	WNT6
P13236	CCL4
Q08211	DHX9
Q92813	DIO2
Q00013	MPP1
P25208	NFYB
Q9H9P8	L2HGDH
O00421	CCRL2
Q5T3J3	LRIF1
Q5UIP0	RIF1
Q9Y581	INSL6
Q969P0	IGSF8

Q9P2E9	RRBP1
Q99567	NUP88
Q9HB63	NTN4
Q92988	DLX4
O95716	RAB3D
Q96JK9	MAML3
E9PAV3	NACA
Q13765	NACA
O95049	TJP3
Q96PC5	MIA2
Q14392	LRRC32
Q9UKJ5	CHIC2
Q9UKY0	PRND
Q9NWB1	RBFOX1
Q96DZ5	CLIP3
O43164	PJA2
P81877	SSBP2
Q9H6H4	REEP4
Q96QS1	TSPAN32
Q15431	SYCP1
P54107	CRISP1
Q9UHD8	SEPTIN9
O75971	SNAPC5
Q8IU54	IFNL1
Q96AQ6	PBXIP1
O75324	SNN
Q9H0V1	TMEM168
Q6ICH7	ASPHD2
Q16517	NNAT
Q8IUR6	CREBRF
Q76B58	BRINP3
P62807	H2BC4
Q7Z5A7	TAFA5
P36888	FLT3
P41180	CASR
Q13285	NR5A1
P23284	PPIB
P07384	CAPN1
P21333	FLNA
O15357	INPPL1
Q9UHD2	TBK1
P00966	ASS1
Q15375	EPHA7

P20309	CHRM3
P43681	CHRNA4
P48058	GRIA4
Q9Y6K9	IKBKG
P07947	YES1
Q9UBS5	GABBR1
P17655	CAPN2
Q02078	MEF2A
O43684	BUB3
P02792	FTL
P20815	CYP3A5
P49427	CDC34
Q13002	GRIK2
P49674	CSNK1E
P35498	SCN1A
P21817	RYR1
P12268	IMPDH2
Q05086	UBE3A
P07225	PROS1
P62834	RAP1A
P05787	KRT8
P02545	LMNA
Q06210	GFPT1
P18846	ATF1
P84077	ARF1
P08243	ASNS
P14867	GABRA1
P18507	GABRG2
P51610	HCFC1
P15882	CHN1
P21917	DRD4
Q9Y463	DYRK1B
P15976	GATA1
P43354	NR4A2
P03952	KLKB1
P07288	KLK3
Q9UGN5	PARP2
P62873	GNB1
O00305	CACNB4
Q92633	LPAR1
P14324	FDPS
P42566	EPS15
O43184	ADAM12

P01023	A2M
P08173	CHRM4
Q16555	DPYSL2
Q05639	EEF1A2
P39086	GRIK1
P50213	IDH3A
Q9HAZ2	PRDM16
Q9H228	S1PR5
P60903	S100A10
O75582	RPS6KA5
P80365	HSD11B2
Q9H0K1	SIK2
P08476	INHBA
P25391	LAMA1
P04350	TUBB4A
P68036	UBE2L3
P19525	EIF2AK2
P29973	CNGA1
Q13148	TARDBP
Q04695	KRT17
P08727	KRT19
Q9Y2U5	MAP3K2
Q12955	ANK3
P43251	BTD
P34059	GALNS
P25440	BRD2
Q12802	AKAP13
P00387	CYB5R3
Q7Z449	CYP2U1
P08311	CTSG
P17927	CR1
P01040	CSTA
P52790	HK3
P17787	CHRNB2
P60842	EIF4A1
Q13562	NEUROD1
P42701	IL12RB1
P14678	SNRPB
Q07699	SCN1B
Q9P212	PLCE1
P43088	PTGFR
Q7Z6Z7	HUWE1
Q9Y4H2	IRS2

Q92995	USP13
Q96FE5	LINGO1
Q9UBU3	GHRL
O00757	FBP2
Q63HM2	PCNX4
P54803	GALC
Q96SW2	CRBN
O76039	CDKL5
Q14541	HNF4G
P50747	HLCS
O43665	RGS10
P01215	CGA
P58340	MLF1
Q9NRI5	DISC1
Q15075	EEA1
P63211	GNGT1
Q16099	GRIK4
Q16478	GRIK5
P12110	COL6A2
O15075	DCLK1
P23975	SLC6A2
Q7Z628	NET1
Q9Y345	SLC6A5
P41220	RGS2
Q15466	NR0B2
P48552	NRIP1
Q13228	SELENBP1
Q08752	PPID
Q92743	HTRA1
O43612	HCRT
P50336	PPOX
Q96RD7	PANX1
Q9HAU4	SMURF2
Q9H4B4	PLK3
P05111	INHA
Q9NZV8	KCND2
P01213	PDYN
P11161	EGR2
P61296	HAND2
P34931	HSPA1L
Q9Y263	PLAA
Q9UM63	PLAGL1
Q92794	KAT6A

Q15208	STK38
P19532	TFE3
Q9H0D6	XRN2
Q96QS3	ARX
O00358	FOXE1
P78347	GTF2I
Q16774	GUK1
Q92820	GGH
Q9P0U3	SENP1
Q13625	TP53BP2
P41227	NAA10
Q13617	CUL2
O75603	GCM2
Q6IB77	GLYAT
P33241	LSP1
O14867	BACH1
Q9BX63	BRIP1
Q9Y3I1	FBXO7
P09972	ALDOC
O75955	FLOT1
P41440	SLC19A1
Q9BZC7	ABCA2
Q11130	FUT7
O43927	CXCL13
Q9UBD6	RHCG
Q9NP71	MLXIPL
Q03591	CFHR1
Q9UJW3	DNMT3L
Q13368	MPP3
Q92796	DLG3
Q16531	DDB1
Q9UBF6	RNF7
Q15784	NEUROD2
Q13261	IL15RA
P55345	PRMT2
Q8N4C6	NIN
P62875	POLR2L
Q9BQE3	TUBA1C
Q9C035	TRIM5
P05162	LGALS2
Q92806	KCNJ9
P52306	RAP1GDS1
Q8NF91	SYNE1

P16519	PCSK2
Q8TDD2	SP7
O96004	HAND1
P04259	KRT6B
O95405	ZFYVE9
P35900	KRT20
Q92585	MAML1
O75891	ALDH1L1
O00219	HAS3
Q13421	MSLN
P55073	DIO3
Q02083	NAAA
O75461	E2F6
Q9H8S9	MOB1A
O15372	EIF3H
O15492	RGS16
Q15406	NR6A1
Q8N0W4	NLGN4X
Q96DU3	SLAMF6
Q9Y6M0	PRSS21
Q9H0F6	SHARPIN
Q92886	NEUROG1
Q12800	TFCP2
Q01085	TIAL1
P24588	AKAP5
Q9UJ14	GGT7
Q9Y6D6	ARFGEF1
Q76L83	ASXL2
Q6PL18	ATAD2
Q9H2G9	BLZF1
O94911	ABCA8
Q9UJA2	CRLS1
P13378	HOXD8
P52951	GBX2
Q13319	CDK5R2
Q07092	COL16A1
P14866	HNRNPL
Q92839	HAS1
Q01658	DR1
P26378	ELAVL4
O14931	NCR3
Q96EB1	ELP4
O76081	RGS20

O75398	DEAF1
P16150	SPN
Q9NPD7	NRN1
P05000	IFNW1
Q9UG01	IFT172
Q9BST9	RTKN
Q9NYR9	NKIRAS2
Q99969	RARRES2
Q86U70	LDB1
Q9BXJ9	NAA15
Q8IXF0	NPAS3
P49685	GPR15
Q9NR30	DDX21
Q9NY93	DDX56
P55884	EIF3B
P60228	EIF3E
Q7Z6J0	SH3RF1
P56470	LGALS4
Q9Y4X4	KLF12
Q8NHU6	TDRD7
O60675	MAFK
O43513	MED7
Q16570	ACKR1
O00590	ACKR2
Q9BPY8	HOPX
Q9BZE4	GTPBP4
Q01850	CDR2
P15822	HIVEP1
Q9Y4C5	CHST2
Q9C005	DPY30
O15541	RNF113A
Q96JQ0	DCHS1
Q9H159	CDH19
P53618	COPB1
P01569	IFNA5
Q86UN2	RTN4RL1
Q9BV35	SLC25A23
Q96CW5	TUBGCP3
Q96Q89	KIF20B
Q99805	TM9SF2
O43692	PI15
Q9C075	KRT23
Q147X3	NAA30

O15457	MSH4
Q86UP3	ZFHX4
Q96KR1	ZFR
Q13503	MED21
Q8TF66	LRRC15
Q9H0B8	CRISPLD2
O43248	HOXC11
Q9Y222	DMTF1
Q8WXI7	MUC16
Q9Y3A3	MOB4
Q8TAT5	NEIL3
Q8TEC5	SH3RF2
P05013	IFNA6
Q86YD1	PTOV1
P50458	LHX2
Q9NYW7	TAS2R1
Q6UXB8	PI16
Q8IZJ0	IFNL2
Q9UF56	FBXL17
Q9C0F0	ASXL3
Q13686	ALKBH1
Q9NPB9	ACKR4
Q9Y5Q8	GTF3C5
Q58WW2	DCAF6
Q6ZMN7	PDZRN4
P01568	IFNA21
Q9HBI0	PARVG
Q86UN3	RTN4RL2
Q8IZD6	SLC22A15
Q07444	KLRC3
Q9BXU1	STK31
P17152	TMEM11
Q8IZI9	IFNL3
Q8IU57	IFNLR1
Q8WTV1	THAP3
Q9BRH9	ZNF251
Q96DT7	ZBTB10
Q7L311	ARMCX2
Q9H336	CRISPLD1
Q969V6	MRTFA
O60519	CREBL2
Q5M775	SPECC1
P05014	IFNA4

P32881	IFNA8
P01566	IFNA10
Q96JJ6	JPH4
Q05215	EGR4
O95389	CCN6
Q9Y692	GMEB1
Q9UH62	ARMCX3
P0C7T5	ATXN1L
Q96LK0	CEP19
Q8WUJ3	CEMIP
Q9Y5L2	HILPDA
Q15369	ELOC
P01567	IFNA7
Q0D2I5	IFFO1
Q96FV3	TSPAN17
Q693B1	KCTD11
P01571	IFNA17
Q96GA3	LTV1
Q8N1E6	FBXL14
Q8N865	C7orf31
Q15370	ELOB
P01570	IFNA14
P05015	IFNA16
Q96FX7	TRMT61A
Q9UKQ9	KLK9
P16619	CCL3L1
O76076	CCN5
Q4G1C9	GLIPR1L2
Q9P291	ARMCX1
Q8NDZ0	BEND2
Q9BY89	KIAA1671
Q08117	TLE5
P13762	HLA-DRB4
Q92599	SEPTIN8
O15446	POLR1G
Q8TF63	DCANP1
A6NI86	GOLGA6L10
Q13072	BAGE

Table S3. Signaling pathways in KEGG enrichment analysis of glioma-related targets

ID	Description	pvalue

hsa04151	PI3K-Akt signaling pathway	9.99E-61
hsa04015	Rap1 signaling pathway	1.02E-39
hsa04010	MAPK signaling pathway	1.84E-36
hsa04933	AGE-RAGE signaling pathway in diabetic complications	3.44E-33
hsa04014	Ras signaling pathway	1.67E-30
hsa04620	Toll-like receptor signaling pathway	1.23E-29
hsa04012	ErbB signaling pathway	1.71E-28
hsa04722	Neurotrophin signaling pathway	1.09E-27
hsa04630	JAK-STAT signaling pathway	1.62E-27
hsa04062	Chemokine signaling pathway	2.76E-27
hsa04668	TNF signaling pathway	3.88E-25
hsa04625	C-type lectin receptor signaling pathway	3.25E-23
hsa04068	FoxO signaling pathway	7.31E-23
hsa04912	GnRH signaling pathway	3.58E-22
hsa04066	HIF-1 signaling pathway	2.64E-21
hsa04660	T cell receptor signaling pathway	1.87E-20
hsa05235	PD-L1 expression and PD-1 checkpoint pathway in cancer	9.66E-20
hsa04024	cAMP signaling pathway	1.29E-19
hsa04926	Relaxin signaling pathway	4.04E-19
hsa04917	Prolactin signaling pathway	7.38E-19
hsa04657	IL-17 signaling pathway	9.33E-19
hsa04020	Calcium signaling pathway	2.30E-18
hsa04915	Estrogen signaling pathway	2.32E-18
hsa04071	Sphingolipid signaling pathway	3.58E-18
hsa04621	NOD-like receptor signaling pathway	3.87E-18
hsa04211	Longevity regulating pathway	4.85E-17
hsa04213	Longevity regulating pathway - multiple species	6.25E-17
hsa04664	Fc epsilon RI signaling pathway	4.85E-16
hsa04064	NF-kappa B signaling pathway	1.19E-15
hsa04390	Hippo signaling pathway	1.20E-15
hsa04072	Phospholipase D signaling pathway	1.60E-15
hsa04371	Apelin signaling pathway	1.83E-15
hsa04350	TGF-beta signaling pathway	1.83E-15
hsa04115	p53 signaling pathway	5.07E-15
hsa04550	Signaling pathways regulating pluripotency of stem cells	9.84E-15
hsa04310	Wnt signaling pathway	1.08E-14
hsa04910	Insulin signaling pathway	1.11E-13
hsa04370	VEGF signaling pathway	7.82E-13
hsa04919	Thyroid hormone signaling pathway	8.41E-13
hsa04022	cGMP-PKG signaling pathway	6.71E-12
hsa04622	RIG-I-like receptor signaling pathway	1.44E-11
hsa04921	Oxytocin signaling pathway	1.01E-10

hsa04150	mTOR signaling pathway	1.33E-09
hsa05022	Pathways of neurodegeneration - multiple diseases	6.67E-09
hsa04920	Adipocytokine signaling pathway	1.57E-08
hsa04662	B cell receptor signaling pathway	5.09E-08
hsa04152	AMPK signaling pathway	6.17E-08
hsa05130	Pathogenic Escherichia coli infection	9.98E-07
hsa05414	Dilated cardiomyopathy	1.57E-06
hsa04623	Cytosolic DNA-sensing pathway	8.21E-06
hsa05410	Hypertrophic cardiomyopathy	0.000168
hsa05412	Arrhythmogenic right ventricular cardiomyopathy	0.000359
hsa05415	Diabetic cardiomyopathy	0.000384
hsa04330	Notch signaling pathway	0.000546
hsa04340	Hedgehog signaling pathway	0.000919
hsa04922	Glucagon signaling pathway	0.002313

Table S4. Result of GO enrichment analysis

Biological process

ID	Description	LogP
GO:0010035	response to inorganic substance	-51.4694
GO:0006979	response to oxidative stress	-41.7269
GO:0097190	apoptotic signaling pathway	-40.6086
GO:0009636	response to toxic substance	-40.318
GO:0034599	cellular response to oxidative stress	-38.5367
GO:0000302	response to reactive oxygen species	-36.1625
GO:0010942	positive regulation of cell death	-34.9653
GO:0071407	cellular response to organic cyclic compound	-34.4405
GO:0010038	response to metal ion	-34.3255
GO:1901699	cellular response to nitrogen compound	-34.0321
GO:1901652	response to peptide	-33.4979

GO:0051347	positive regulation of transferase activity	-33.1327
GO:0071396	cellular response to lipid	-32.9785
GO:0034614	cellular response to reactive oxygen species	-32.0839
GO:0009611	response to wounding	-31.6759
GO:0072593	reactive oxygen species metabolic process	-31.5893
GO:0071241	cellular response to inorganic substance	-31.351
GO:0033674	positive regulation of kinase activity	-31.0399
GO:0070997	neuron death	-30.7141
GO:0071417	cellular response to organonitrogen compound	-30.2748
GO:0043065	positive regulation of apoptotic process	-30.0645
GO:0043068	positive regulation of programmed cell death	-29.8179
GO:0046677	response to antibiotic	-29.6909
GO:0032496	response to lipopolysaccharide	-29.3228
GO:0030335	positive regulation of cell migration	-29.1178
GO:0071900	regulation of protein serine/threonine kinase activity	-29.0829
GO:0071248	cellular response to metal ion	-28.8839
GO:0035690	cellular response to drug	-28.7306
GO:2001233	regulation of apoptotic signaling pathway	-28.5335
GO:0002237	response to molecule of bacterial origin	-28.3591
GO:2000147	positive regulation of cell motility	-28.3352
GO:0018209	peptidyl-serine modification	-28.0928
GO:0018105	peptidyl-serine phosphorylation	-27.854

GO:0051272	positive regulation of cellular component movement	-27.8157
GO:0040017	positive regulation of locomotion	-27.5346
GO:0048732	gland development	-27.3986
GO:0045860	positive regulation of protein kinase activity	-26.5444
GO:0071902	positive regulation of protein serine/threonine kinase activity	-26.21
GO:0007568	aging	-26.1821
GO:0043410	positive regulation of MAPK cascade	-26.1028
GO:1901214	regulation of neuron death	-26.096
GO:0071216	cellular response to biotic stimulus	-25.7531
GO:0030155	regulation of cell adhesion	-25.5956
GO:0048608	reproductive structure development	-25.0777
GO:0042060	wound healing	-25.0686
GO:0071363	cellular response to growth factor stimulus	-25.0242
GO:0061458	reproductive system development	-24.9463
GO:0070848	response to growth factor	-24.333
GO:0001568	blood vessel development	-24.3322
GO:0097237	cellular response to toxic substance	-24.2388
GO:0009617	response to bacterium	-24.2209
GO:2000377	regulation of reactive oxygen species metabolic process	-24.2169
GO:0009991	response to extracellular stimulus	-24.1556
GO:2001234	negative regulation of apoptotic signaling pathway	-23.777
GO:0080135	regulation of cellular response to stress	-23.6944

GO:0046686	response to cadmium ion	-23.3374
GO:0002521	leukocyte differentiation	-23.3315
GO:0050673	epithelial cell proliferation	-23.2873
GO:0033002	muscle cell proliferation	-23.2338
GO:0051402	neuron apoptotic process	-23.1858
GO:0043408	regulation of MAPK cascade	-23.1449
GO:0032870	cellular response to hormone stimulus	-22.901
GO:0018108	peptidyl-tyrosine phosphorylation	-22.8069
GO:0018212	peptidyl-tyrosine modification	-22.7031
GO:0001101	response to acid chemical	-22.6362
GO:0097193	intrinsic apoptotic signaling pathway	-22.4439
GO:0071222	cellular response to lipopolysaccharide	-22.4267
GO:0007169	transmembrane receptor protein tyrosine kinase signaling pathway	-22.2768
GO:0043406	positive regulation of MAP kinase activity	-22.1815
GO:0031667	response to nutrient levels	-21.8571
GO:0050678	regulation of epithelial cell proliferation	-21.8083
GO:0022407	regulation of cell-cell adhesion	-21.8053
GO:0071219	cellular response to molecule of bacterial origin	-21.7146
GO:0043434	response to peptide hormone	-21.5675
GO:0048545	response to steroid hormone	-21.4317
GO:0097191	extrinsic apoptotic signaling pathway	-21.3299
GO:0071496	cellular response to external stimulus	-21.2578

GO:0001819	positive regulation of cytokine production	-21.1626
GO:2000379	positive regulation of reactive oxygen species metabolic process	-20.5312
GO:0009314	response to radiation	-20.5137
GO:0043491	protein kinase B signaling	-20.4953
GO:0003006	developmental process involved in reproduction	-20.4133
GO:0048514	blood vessel morphogenesis	-20.3509
GO:0043405	regulation of MAP kinase activity	-20.3159
GO:0048660	regulation of smooth muscle cell proliferation	-20.3123
GO:0009410	response to xenobiotic stimulus	-20.2201
GO:0048659	smooth muscle cell proliferation	-20.2008
GO:0001666	response to hypoxia	-19.9181
GO:0070482	response to oxygen levels	-19.868
GO:0010638	positive regulation of organelle organization	-19.8457
GO:0032147	activation of protein kinase activity	-19.7168
GO:0050900	leukocyte migration	-19.6967
GO:0051098	regulation of binding	-19.6294
GO:0036293	response to decreased oxygen levels	-19.5349
GO:0032103	positive regulation of response to external stimulus	-19.4469
GO:0048511	rhythmic process	-19.438
GO:0042063	gliogenesis	-19.4023
GO:0051090	regulation of DNA-binding transcription factor activity	-19.2575
GO:0050727	regulation of inflammatory response	-19.1593

GO:0045785	positive regulation of cell adhesion	-18.9401
GO:1901654	response to ketone	-18.9156
GO:1903409	reactive oxygen species biosynthetic process	-18.9006
GO:1904645	response to amyloid-beta	-18.7771
GO:0008285	negative regulation of cell proliferation	-18.7755
GO:0050865	regulation of cell activation	-18.6105
GO:0062012	regulation of small molecule metabolic process	-18.5795
GO:0002573	myeloid leukocyte differentiation	-18.4061
GO:0062013	positive regulation of small molecule metabolic process	-18.3457
GO:0043523	regulation of neuron apoptotic process	-18.2722
GO:0071276	cellular response to cadmium ion	-18.2623
GO:1901216	positive regulation of neuron death	-18.2383
GO:1902895	positive regulation of pri-miRNA transcription by RNA polymerase II	-17.9211
GO:0070201	regulation of establishment of protein localization	-17.7775
GO:0002064	epithelial cell development	-17.7572
GO:0030099	myeloid cell differentiation	-17.756
GO:0051046	regulation of secretion	-17.6083
GO:0001525	angiogenesis	-17.5829
GO:1901653	cellular response to peptide	-17.5441
GO:1904951	positive regulation of establishment of protein localization	-17.5167
GO:2001237	negative regulation of extrinsic apoptotic signaling pathway	-17.4777
GO:0032787	monocarboxylic acid metabolic process	-17.4545

GO:0097305	response to alcohol	-17.3906
GO:0046777	protein autophosphorylation	-17.3112
GO:0045596	negative regulation of cell differentiation	-17.2708
GO:0048871	multicellular organismal homeostasis	-17.2534
GO:0009896	positive regulation of catabolic process	-17.2433
GO:0000187	activation of MAPK activity	-17.1779
GO:0090087	regulation of peptide transport	-17.1662
GO:1903827	regulation of cellular protein localization	-17.0097
GO:0031668	cellular response to extracellular stimulus	-16.8279
GO:0032355	response to estradiol	-16.7191
GO:0051896	regulation of protein kinase B signaling	-16.7024
GO:1904019	epithelial cell apoptotic process	-16.6008
GO:0007159	leukocyte cell-cell adhesion	-16.4877
GO:0046649	lymphocyte activation	-16.4781
GO:1903037	regulation of leukocyte cell-cell adhesion	-16.4334
GO:0010631	epithelial cell migration	-16.4316
GO:0010001	glial cell differentiation	-16.3926
GO:0090132	epithelium migration	-16.3481
GO:0048661	positive regulation of smooth muscle cell proliferation	-16.3138
GO:1902893	regulation of pri-miRNA transcription by RNA polymerase II	-16.232
GO:0010634	positive regulation of epithelial cell migration	-16.1859
GO:0090130	tissue migration	-16.1834

GO:1903829	positive regulation of cellular protein localization	-16.1665
GO:1903706	regulation of hemopoiesis	-16.134
GO:0045786	negative regulation of cell cycle	-16.1204
GO:0061614	pri-miRNA transcription by RNA polymerase II	-16.1126
GO:0001935	endothelial cell proliferation	-16.1117
GO:0050679	positive regulation of epithelial cell proliferation	-15.9484
GO:0010506	regulation of autophagy	-15.8791
GO:0022409	positive regulation of cell-cell adhesion	-15.8
GO:0070661	leukocyte proliferation	-15.7998
GO:0031331	positive regulation of cellular catabolic process	-15.7844
GO:0050878	regulation of body fluid levels	-15.7836
GO:0046394	carboxylic acid biosynthetic process	-15.7013
GO:0016053	organic acid biosynthetic process	-15.6787
GO:0031960	response to corticosteroid	-15.5298
GO:0018107	peptidyl-threonine phosphorylation	-15.4001
GO:0050731	positive regulation of peptidyl-tyrosine phosphorylation	-15.3949
GO:1903034	regulation of response to wounding	-15.3121
GO:0046425	regulation of JAK-STAT cascade	-15.2833
GO:1904035	regulation of epithelial cell apoptotic process	-15.2244
GO:0050730	regulation of peptidyl-tyrosine phosphorylation	-15.2078
GO:1903530	regulation of secretion by cell	-15.1454
GO:0042542	response to hydrogen peroxide	-15.1291

GO:0045862	positive regulation of proteolysis	-15.0448
GO:0030522	intracellular receptor signaling pathway	-15.043
GO:0051223	regulation of protein transport	-15.0388
GO:1904018	positive regulation of vasculature development	-15.0364
GO:0071236	cellular response to antibiotic	-14.9785
GO:0051897	positive regulation of protein kinase B signaling	-14.8939
GO:0018210	peptidyl-threonine modification	-14.8753
GO:0051249	regulation of lymphocyte activation	-14.8583
GO:2000351	regulation of endothelial cell apoptotic process	-14.8555
GO:0009612	response to mechanical stimulus	-14.8343
GO:1904892	regulation of STAT cascade	-14.8312
GO:0060326	cell chemotaxis	-14.7824
GO:0002791	regulation of peptide secretion	-14.7082
GO:0072330	monocarboxylic acid biosynthetic process	-14.6966
GO:0007346	regulation of mitotic cell cycle	-14.6358
GO:0031349	positive regulation of defense response	-14.5936
GO:0001667	ameboidal-type cell migration	-14.5867
GO:0060249	anatomical structure homeostasis	-14.5712
GO:0009416	response to light stimulus	-14.554
GO:0043281	regulation of cysteine-type endopeptidase activity involved in apoptotic process	-14.4957
GO:0031669	cellular response to nutrient levels	-14.4847
GO:1901342	regulation of vasculature development	-14.4364

GO:0072577	endothelial cell apoptotic process	-14.4084	
GO:0002683	negative regulation of immune system process	-14.3971	
GO:0051222	positive regulation of protein transport	-14.3848	
GO:2001236	regulation of extrinsic apoptotic signaling pathway	-14.3626	
GO:0001936	regulation of endothelial cell proliferation	-14.3517	
GO:0040008	regulation of growth	-14.3317	
GO:0031334	positive regulation of protein complex assembly	-14.327	
GO:1902105	regulation of leukocyte differentiation	-14.2974	
GO:0061041	regulation of wound healing	-14.2284	
GO:0009411	response to UV	-14.0876	
GO:0051186	cofactor metabolic process	-14.0763	
GO:0006935	chemotaxis	-14.0258	
GO:0043269	regulation of ion transport	-14.0207	
GO:0051100	negative regulation of binding	-14.0105	
GO:0010632	regulation of epithelial cell migration	-14.0069	
GO:0003013	circulatory system process	-13.9921	
GO:0042330	taxis		-13.992
GO:0002694	regulation of leukocyte activation	-13.9871	
GO:0007050	cell cycle arrest	-13.8969	
GO:0051091	positive regulation of DNA-binding transcription factor activity	-13.8789	
GO:0031663	lipopolysaccharide-mediated signaling pathway	-13.8466	
GO:0006690	icosanoid metabolic process	-13.7635	

GO:1903708	positive regulation of hemopoiesis	-13.7385
GO:0070663	regulation of leukocyte proliferation	-13.7301
GO:0071383	cellular response to steroid hormone stimulus	-13.702
GO:0034504	protein localization to nucleus	-13.6678
GO:2000116	regulation of cysteine-type endopeptidase activity	-13.6644
GO:0043536	positive regulation of blood vessel endothelial cell migration	-13.614
GO:1900182	positive regulation of protein localization to nucleus	-13.614
GO:0070555	response to interleukin-1	-13.5935
GO:0000079	regulation of cyclin-dependent protein serine/threonine kinase activity	-13.5602
GO:0045639	positive regulation of myeloid cell differentiation	-13.5602
GO:1902882	regulation of response to oxidative stress	-13.5602
GO:1901215	negative regulation of neuron death	-13.5578
GO:0071466	cellular response to xenobiotic stimulus	-13.5556
GO:0034612	response to tumor necrosis factor	-13.5107
GO:1900180	regulation of protein localization to nucleus	-13.4997
GO:0006914	autophagy	-13.4688
GO:0061919	process utilizing autophagic mechanism	-13.4688
GO:0032868	response to insulin	-13.4338
GO:0050708	regulation of protein secretion	-13.4247
GO:0043254	regulation of protein complex assembly	-13.3688
GO:0001890	placenta development	-13.3445
GO:1904029	regulation of cyclin-dependent protein kinase activity	-13.3214

GO:0008015	blood circulation	-13.3018
GO:0010817	regulation of hormone levels	-13.2652
GO:0042136	neurotransmitter biosynthetic process	-13.2058
GO:0008202	steroid metabolic process	-13.1972
GO:0070371	ERK1 and ERK2 cascade	-13.1972
GO:0051047	positive regulation of secretion	-13.1651
GO:0042035	regulation of cytokine biosynthetic process	-13.1398
GO:0007259	JAK-STAT cascade	-13.1296
GO:0007565	female pregnancy	-12.9828
GO:0042108	positive regulation of cytokine biosynthetic process	-12.9818
GO:0045637	regulation of myeloid cell differentiation	-12.9803
GO:2001242	regulation of intrinsic apoptotic signaling pathway	-12.9629
GO:0043200	response to amino acid	-12.8727
GO:0046427	positive regulation of JAK-STAT cascade	-12.8604
GO:0019216	regulation of lipid metabolic process	-12.8575
GO:0031099	regeneration	-12.8027
GO:1905114	cell surface receptor signaling pathway involved in cell-cell signaling	-12.8
GO:1900407	regulation of cellular response to oxidative stress	-12.797
GO:1903039	positive regulation of leukocyte cell-cell adhesion	-12.7838
GO:0030879	mammary gland development	-12.7732
GO:0042089	cytokine biosynthetic process	-12.7451
GO:0097696	STAT cascade	-12.7212

GO:1902532	negative regulation of intracellular signal transduction	-12.7162
GO:0042110	T cell activation	-12.7115
GO:1904894	positive regulation of STAT cascade	-12.6725
GO:0042107	cytokine metabolic process	-12.6699
GO:0045787	positive regulation of cell cycle	-12.664
GO:0046660	female sex differentiation	-12.6098
GO:0003018	vascular process in circulatory system	-12.6038
GO:1901568	fatty acid derivative metabolic process	-12.6038
GO:0016572	histone phosphorylation	-12.6038
GO:0006801	superoxide metabolic process	-12.5959
GO:0098771	inorganic ion homeostasis	-12.5727
GO:0045766	positive regulation of angiogenesis	-12.5589
GO:0051384	response to glucocorticoid	-12.5499
GO:0051054	positive regulation of DNA metabolic process	-12.5345
GO:0007548	sex differentiation	-12.4945
GO:0071229	cellular response to acid chemical	-12.4909
GO:0042445	hormone metabolic process	-12.4736
GO:0043393	regulation of protein binding	-12.4237
GO:0014074	response to purine-containing compound	-12.42
GO:0046651	lymphocyte proliferation	-12.4123
GO:0002761	regulation of myeloid leukocyte differentiation	-12.4082
GO:0001505	regulation of neurotransmitter levels	-12.3979

GO:0060627	regulation of vesicle-mediated transport	-12.3871
GO:0002763	positive regulation of myeloid leukocyte differentiation	-12.3789
GO:0019369	arachidonic acid metabolic process	-12.3789
GO:0048771	tissue remodeling	-12.3385
GO:0032943	mononuclear cell proliferation	-12.331
GO:0043542	endothelial cell migration	-12.331
GO:0008637	apoptotic mitochondrial changes	-12.3102
GO:0045834	positive regulation of lipid metabolic process	-12.293
GO:0045981	positive regulation of nucleotide metabolic process	-12.292
GO:1900544	positive regulation of purine nucleotide metabolic process	-12.292
GO:0002790	peptide secretion	-12.2831
GO:1901615	organic hydroxy compound metabolic process	-12.2831
GO:0014065	phosphatidylinositol 3-kinase signaling	-12.2512
GO:0006809	nitric oxide biosynthetic process	-12.2417
GO:0014066	regulation of phosphatidylinositol 3-kinase signaling	-12.2138
GO:0071375	cellular response to peptide hormone stimulus	-12.1948
GO:0007584	response to nutrient	-12.1939
GO:0071214	cellular response to abiotic stimulus	-12.1706
GO:0104004	cellular response to environmental stimulus	-12.1706
GO:0042133	neurotransmitter metabolic process	-12.1687
GO:0072594	establishment of protein localization to organelle	-12.1633
GO:0048872	homeostasis of number of cells	-12.1197

GO:0045080	positive regulation of chemokine biosynthetic process	-12.1066
GO:0043534	blood vessel endothelial cell migration	-12.0491
GO:0030168	platelet activation	-12.047
GO:0008610	lipid biosynthetic process	-12.0045
GO:0044706	multi-multicellular organism process	-11.9722
GO:1902107	positive regulation of leukocyte differentiation	-11.9674
GO:0031098	stress-activated protein kinase signaling cascade	-11.9387
GO:0010595	positive regulation of endothelial cell migration	-11.9347
GO:0098754	detoxification	-11.9347
GO:0046209	nitric oxide metabolic process	-11.9118
GO:0007596	blood coagulation	-11.9104
GO:1905475	regulation of protein localization to membrane	-11.875
GO:0046683	response to organophosphorus	-11.8448
GO:0007599	hemostasis	-11.7953
GO:0050817	coagulation	-11.7725
GO:0009615	response to virus	-11.7498
GO:1904646	cellular response to amyloid-beta	-11.7385
GO:0009743	response to carbohydrate	-11.7282
GO:2001057	reactive nitrogen species metabolic process	-11.7243
GO:0070372	regulation of ERK1 and ERK2 cascade	-11.6893
GO:0032102	negative regulation of response to external stimulus	-11.6779
GO:0001938	positive regulation of endothelial cell proliferation	-11.6199

GO:0008625	extrinsic apoptotic signaling pathway via death domain receptors	-11.6033
GO:1990748	cellular detoxification	-11.5701
GO:0045765	regulation of angiogenesis	-11.5545
GO:0042594	response to starvation	-11.5092
GO:0048146	positive regulation of fibroblast proliferation	-11.4559
GO:0071456	cellular response to hypoxia	-11.445
GO:0043270	positive regulation of ion transport	-11.3879
GO:0033559	unsaturated fatty acid metabolic process	-11.3756
GO:0038127	ERBB signaling pathway	-11.3755
GO:0001503	ossification	-11.3733
GO:0007005	mitochondrion organization	-11.3647
GO:0048589	developmental growth	-11.3162
GO:0002685	regulation of leukocyte migration	-11.2876
GO:0051403	stress-activated MAPK cascade	-11.2862
GO:0044089	positive regulation of cellular component biogenesis	-11.2838
GO:0009306	protein secretion	-11.2323
GO:0050863	regulation of T cell activation	-11.217
GO:0052547	regulation of peptidase activity	-11.2074
GO:0045073	regulation of chemokine biosynthetic process	-11.2048
GO:0051924	regulation of calcium ion transport	-11.1846
GO:0001959	regulation of cytokine-mediated signaling pathway	-11.158
GO:2001235	positive regulation of apoptotic signaling pathway	-11.158

GO:1901655	cellular response to ketone	-11.1479
GO:0045471	response to ethanol	-11.1427
GO:0055080	cation homeostasis	-11.1394
GO:0036294	cellular response to decreased oxygen levels	-11.1344
GO:0070374	positive regulation of ERK1 and ERK2 cascade	-11.1042
GO:0036473	cell death in response to oxidative stress	-11.094
GO:2001243	negative regulation of intrinsic apoptotic signaling pathway	-11.094
GO:0031100	animal organ regeneration	-11.0932
GO:0038034	signal transduction in absence of ligand	-11.0932
GO:0097192	extrinsic apoptotic signaling pathway in absence of ligand	-11.0932
GO:0071356	cellular response to tumor necrosis factor	-11.0633
GO:0022612	gland morphogenesis	-11.0524
GO:0045930	negative regulation of mitotic cell cycle	-11.0151
GO:0072511	divalent inorganic cation transport	-10.9935
GO:0008585	female gonad development	-10.9879
GO:0008406	gonad development	-10.9851
GO:0007162	negative regulation of cell adhesion	-10.9667
GO:0150076	neuroinflammatory response	-10.9639
GO:1903201	regulation of oxidative stress-induced cell death	-10.9639
GO:0042033	chemokine biosynthetic process	-10.9577
GO:0050755	chemokine metabolic process	-10.9577
GO:0097529	myeloid leukocyte migration	-10.9557

GO:0090068	positive regulation of cell cycle process	-10.9428
GO:0052548	regulation of endopeptidase activity	-10.9117
GO:0048015	phosphatidylinositol-mediated signaling	-10.8579
GO:0002793	positive regulation of peptide secretion	-10.8395
GO:0008630	intrinsic apoptotic signaling pathway in response to DNA damage	-10.8331
GO:0098869	cellular oxidant detoxification	-10.8331
GO:0051092	positive regulation of NF-kappaB transcription factor activity	-10.8325
GO:0045137	development of primary sexual characteristics	-10.8108
GO:0010959	regulation of metal ion transport	-10.7987
GO:0072331	signal transduction by p53 class mediator	-10.7873
GO:0043525	positive regulation of neuron apoptotic process	-10.7863
GO:0042176	regulation of protein catabolic process	-10.779
GO:0046545	development of primary female sexual characteristics	-10.7327
GO:0048017	inositol lipid-mediated signaling	-10.7296
GO:0060759	regulation of response to cytokine stimulus	-10.7296
GO:0009267	cellular response to starvation	-10.723
GO:0030595	leukocyte chemotaxis	-10.6975
GO:0045936	negative regulation of phosphate metabolic process	-10.6809
GO:0009266	response to temperature stimulus	-10.6695
GO:0010563	negative regulation of phosphorus metabolic process	-10.6661
GO:0043535	regulation of blood vessel endothelial cell migration	-10.6513
GO:1901361	organic cyclic compound catabolic process	-10.6446

GO:0071453	cellular response to oxygen levels	-10.6417
GO:0032722	positive regulation of chemokine production	-10.6348
GO:1903426	regulation of reactive oxygen species biosynthetic process	-10.6344
GO:0050804	modulation of chemical synaptic transmission	-10.6025
GO:0048145	regulation of fibroblast proliferation	-10.5974
GO:0048708	astrocyte differentiation	-10.5974
GO:0071887	leukocyte apoptotic process	-10.586
GO:0099177	regulation of trans-synaptic signaling	-10.5847
GO:0051345	positive regulation of hydrolase activity	-10.5692
GO:0045931	positive regulation of mitotic cell cycle	-10.5456
GO:0048144	fibroblast proliferation	-10.5392
GO:0010594	regulation of endothelial cell migration	-10.5316
GO:0010564	regulation of cell cycle process	-10.4698
GO:0030098	lymphocyte differentiation	-10.4444
GO:0006873	cellular ion homeostasis	-10.433
GO:0003158	endothelium development	-10.4232
GO:0050714	positive regulation of protein secretion	-10.3914
GO:0001889	liver development	-10.384
GO:0045055	regulated exocytosis	-10.3839
GO:1904705	regulation of vascular smooth muscle cell proliferation	-10.3692
GO:1990874	vascular smooth muscle cell proliferation	-10.3692
GO:0034284	response to monosaccharide	-10.3617

GO:0001676	long-chain fatty acid metabolic process	-10.3514
GO:0042113	B cell activation	-10.3511
GO:0007249	I-kappaB kinase/NF-kappaB signaling	-10.3429
GO:0002697	regulation of immune effector process	-10.3237
GO:0014068	positive regulation of phosphatidylinositol 3-kinase signaling	-10.314
GO:0032642	regulation of chemokine production	-10.314
GO:0045737	positive regulation of cyclin-dependent protein serine/threonine kinase activity	-10.3078
GO:0035296	regulation of tube diameter	-10.3064
GO:0050880	regulation of blood vessel size	-10.3064
GO:0097746	regulation of blood vessel diameter	-10.3064
GO:0006109	regulation of carbohydrate metabolic process	-10.3027
GO:0034764	positive regulation of transmembrane transport	-10.3027
GO:0035150	regulation of tube size	-10.2681
GO:0061008	hepaticobiliary system development	-10.2681
GO:0043620	regulation of DNA-templated transcription in response to stress	-10.2608
GO:0002764	immune response-regulating signaling pathway	-10.2584
GO:0070838	divalent metal ion transport	-10.2389
GO:0002700	regulation of production of molecular mediator of immune response	-10.2301
GO:0030810	positive regulation of nucleotide biosynthetic process	-10.1771
GO:1900373	positive regulation of purine nucleotide biosynthetic process	-10.1771
GO:1903580	positive regulation of ATP metabolic process	-10.1771
GO:0034976	response to endoplasmic reticulum stress	-10.1555

GO:1900542	regulation of purine nucleotide metabolic process	-10.1178
GO:0043524	negative regulation of neuron apoptotic process	-10.0809
GO:0007623	circadian rhythm	-10.073
GO:1903532	positive regulation of secretion by cell	-10.0546
GO:0071347	cellular response to interleukin-1	-10.0469
GO:0050670	regulation of lymphocyte proliferation	-10.045
GO:0042698	ovulation cycle	-10.0163
GO:1904036	negative regulation of epithelial cell apoptotic process	-10.0082
GO:0032944	regulation of mononuclear cell proliferation	-9.98935
GO:0006140	regulation of nucleotide metabolic process	-9.97194
GO:0042759	long-chain fatty acid biosynthetic process	-9.95392
GO:0032602	chemokine production	-9.94602
GO:0051129	negative regulation of cellular component organization	-9.92985
GO:0006984	ER-nucleus signaling pathway	-9.92664
GO:0044772	mitotic cell cycle phase transition	-9.91465
GO:0006631	fatty acid metabolic process	-9.90209
GO:0006721	terpenoid metabolic process	-9.87372
GO:0051101	regulation of DNA binding	-9.87372
GO:0042100	B cell proliferation	-9.84643
GO:0002263	cell activation involved in immune response	-9.84526
GO:1904031	positive regulation of cyclin-dependent protein kinase activity	-9.84394
GO:0030003	cellular cation homeostasis	-9.84001

GO:0044843	cell cycle G1/S phase transition	-9.79793
GO:0030316	osteoclast differentiation	-9.79748
GO:0051052	regulation of DNA metabolic process	-9.79481
GO:0048729	tissue morphogenesis	-9.78729
GO:0002009	morphogenesis of an epithelium	-9.77194
GO:0051881	regulation of mitochondrial membrane potential	-9.7694
GO:0034405	response to fluid shear stress	-9.73751
GO:2000352	negative regulation of endothelial cell apoptotic process	-9.73751
GO:0014013	regulation of gliogenesis	-9.71165
GO:0098542	defense response to other organism	-9.70553
GO:0070301	cellular response to hydrogen peroxide	-9.70125
GO:0010821	regulation of mitochondrion organization	-9.68013
GO:0043062	extracellular structure organization	-9.67751
GO:0070423	nucleotide-binding oligomerization domain containing signaling pathway	-9.63443
GO:0043467	regulation of generation of precursor metabolites and energy	-9.62543
GO:0099536	synaptic signaling	-9.61058
GO:0043112	receptor metabolic process	-9.53477
GO:0035872	nucleotide-binding domain, leucine rich repeat containing receptor signaling pathway	-9.53449
GO:0022411	cellular component disassembly	-9.52608
GO:0016055	Wnt signaling pathway	-9.52053
GO:0043279	response to alkaloid	-9.51508
GO:0006091	generation of precursor metabolites and energy	-9.49035

GO:0198738	cell-cell signaling by wnt	-9.49035
GO:0034754	cellular hormone metabolic process	-9.47888
GO:0009408	response to heat	-9.46109
GO:0050867	positive regulation of cell activation	-9.45468
GO:0009746	response to hexose	-9.44952
GO:0019233	sensory perception of pain	-9.42496
GO:0006816	calcium ion transport	-9.36897
GO:0001655	urogenital system development	-9.35976
GO:0000082	G1/S transition of mitotic cell cycle	-9.35515
GO:0050866	negative regulation of cell activation	-9.33805
GO:0030100	regulation of endocytosis	-9.3331
GO:0046456	icosanoid biosynthetic process	-9.3326
GO:0044770	cell cycle phase transition	-9.3054
GO:0043618	regulation of transcription from RNA polymerase II promoter in response to stress	-9.29329
GO:0006633	fatty acid biosynthetic process	-9.27105
GO:0097755	positive regulation of blood vessel diameter	-9.26476
GO:1903428	positive regulation of reactive oxygen species biosynthetic process	-9.26476
GO:0042326	negative regulation of phosphorylation	-9.25986
GO:0038083	peptidyl-tyrosine autophosphorylation	-9.25177
GO:1902042	negative regulation of extrinsic apoptotic signaling pathway via death domain receptors	-9.25177
GO:0001659	temperature homeostasis	-9.2401
GO:0045913	positive regulation of carbohydrate metabolic process	-9.21225

GO:0055065	metal ion homeostasis	-9.17708
GO:0043154	negative regulation of cysteine-type endopeptidase activity involved in apoptotic process	-9.16065
GO:0002366	leukocyte activation involved in immune response	-9.15952
GO:0007254	JNK cascade	-9.14875
GO:0071384	cellular response to corticosteroid stimulus	-9.1328
GO:0051301	cell division	-9.12652
GO:0006720	isoprenoid metabolic process	-9.11577
GO:0050921	positive regulation of chemotaxis	-9.11577
GO:0001776	leukocyte homeostasis	-9.10972
GO:0042446	hormone biosynthetic process	-9.10972
GO:1902175	regulation of oxidative stress-induced intrinsic apoptotic signaling pathway	-9.09729
GO:0007268	chemical synaptic transmission	-9.08786
GO:0098916	anterograde trans-synaptic signaling	-9.08786
GO:0008631	intrinsic apoptotic signaling pathway in response to oxidative stress	-9.07605
GO:0032386	regulation of intracellular transport	-9.07006
GO:0044270	cellular nitrogen compound catabolic process	-9.0405
GO:0030198	extracellular matrix organization	-9.01353
GO:0051493	regulation of cytoskeleton organization	-9.01178
GO:0060135	maternal process involved in female pregnancy	-9.00552
GO:0099537	trans-synaptic signaling	-8.99347
GO:0001892	embryonic placenta development	-8.91241
GO:20000	regulation of G1/S transition of mitotic cell cycle	-8.9124

45		
GO:0070665	positive regulation of leukocyte proliferation	-8.91125
GO:0045429	positive regulation of nitric oxide biosynthetic process	-8.90933
GO:0002253	activation of immune response	-8.90036
GO:0072521	purine-containing compound metabolic process	-8.87733
GO:0051251	positive regulation of lymphocyte activation	-8.86021
GO:0002696	positive regulation of leukocyte activation	-8.85943
GO:0038061	NIK/NF-kappaB signaling	-8.85519
GO:0061061	muscle structure development	-8.85451
GO:0016999	antibiotic metabolic process	-8.84512
GO:1900371	regulation of purine nucleotide biosynthetic process	-8.84299
GO:0045732	positive regulation of protein catabolic process	-8.84013
GO:0045088	regulation of innate immune response	-8.83183
GO:1904407	positive regulation of nitric oxide metabolic process	-8.82908
GO:0044057	regulation of system process	-8.82607
GO:0030808	regulation of nucleotide biosynthetic process	-8.80436
GO:0032869	cellular response to insulin stimulus	-8.79052
GO:2000117	negative regulation of cysteine-type endopeptidase activity	-8.77074
GO:0021782	glial cell development	-8.76609
GO:0045446	endothelial cell differentiation	-8.76609
GO:0050920	regulation of chemotaxis	-8.7659
GO:0045428	regulation of nitric oxide biosynthetic process	-8.76378

GO:1904375	regulation of protein localization to cell periphery	-8.72817
GO:0001894	tissue homeostasis	-8.71702
GO:0034762	regulation of transmembrane transport	-8.71162
GO:0090066	regulation of anatomical structure size	-8.70997
GO:0001885	endothelial cell development	-8.70583
GO:0002753	cytoplasmic pattern recognition receptor signaling pathway	-8.70583
GO:2000573	positive regulation of DNA biosynthetic process	-8.70583
GO:0090407	organophosphate biosynthetic process	-8.69892
GO:0007173	epidermal growth factor receptor signaling pathway	-8.69059
GO:0051591	response to cAMP	-8.6791
GO:0030968	endoplasmic reticulum unfolded protein response	-8.65335
GO:1903578	regulation of ATP metabolic process	-8.65335
GO:0031400	negative regulation of protein modification process	-8.63739
GO:0001818	negative regulation of cytokine production	-8.62623
GO:0061180	mammary gland epithelium development	-8.59267
GO:1905477	positive regulation of protein localization to membrane	-8.57986
GO:0009749	response to glucose	-8.55248
GO:0048010	vascular endothelial growth factor receptor signaling pathway	-8.54559
GO:0008210	estrogen metabolic process	-8.53209
GO:0038128	ERBB2 signaling pathway	-8.53209
GO:0071715	icosanoid transport	-8.52661
GO:1901571	fatty acid derivative transport	-8.52661

GO:1904707	positive regulation of vascular smooth muscle cell proliferation	-8.52661
GO:0060359	response to ammonium ion	-8.50766
GO:0032872	regulation of stress-activated MAPK cascade	-8.50296
GO:0097327	response to antineoplastic agent	-8.5021
GO:0071897	DNA biosynthetic process	-8.4995
GO:0072657	protein localization to membrane	-8.47826
GO:0006066	alcohol metabolic process	-8.46988
GO:0009117	nucleotide metabolic process	-8.45515
GO:0002757	immune response-activating signal transduction	-8.4421
GO:0070302	regulation of stress-activated protein kinase signaling cascade	-8.43364
GO:0006909	phagocytosis	-8.41873
GO:0032637	interleukin-8 production	-8.41657
GO:0035924	cellular response to vascular endothelial growth factor stimulus	-8.37666
GO:0006753	nucleoside phosphate metabolic process	-8.37589
GO:1903076	regulation of protein localization to plasma membrane	-8.37451
GO:0001701	in utero embryonic development	-8.36807
GO:0042770	signal transduction in response to DNA damage	-8.36695
GO:1902806	regulation of cell cycle G1/S phase transition	-8.34412
GO:0042593	glucose homeostasis	-8.34275
GO:0046700	heterocycle catabolic process	-8.34221
GO:0009165	nucleotide biosynthetic process	-8.33455
GO:1901570	fatty acid derivative biosynthetic process	-8.33291

GO:0033500	carbohydrate homeostasis	-8.32029
GO:0046890	regulation of lipid biosynthetic process	-8.31873
GO:0045089	positive regulation of innate immune response	-8.31787
GO:0061756	leukocyte adhesion to vascular endothelial cell	-8.31706
GO:0007610	behavior	-8.29932
GO:0010042	response to manganese ion	-8.29246
GO:0032635	interleukin-6 production	-8.29075
GO:1901293	nucleoside phosphate biosynthetic process	-8.28466
GO:0044070	regulation of anion transport	-8.25105
GO:0001933	negative regulation of protein phosphorylation	-8.25102
GO:0032757	positive regulation of interleukin-8 production	-8.25014
GO:0051194	positive regulation of cofactor metabolic process	-8.2396
GO:0030856	regulation of epithelial cell differentiation	-8.23328
GO:0050806	positive regulation of synaptic transmission	-8.23328
GO:0043122	regulation of I-kappaB kinase/NF-kappaB signaling	-8.23149
GO:0071156	regulation of cell cycle arrest	-8.21077
GO:0019439	aromatic compound catabolic process	-8.19831
GO:0009145	purine nucleoside triphosphate biosynthetic process	-8.17657
GO:0051099	positive regulation of binding	-8.17657
GO:0032091	negative regulation of protein binding	-8.17091
GO:0036499	PERK-mediated unfolded protein response	-8.14898
GO:00105	regulation of platelet activation	-8.14838

43		
GO:0010332	response to gamma radiation	-8.12037
GO:0033138	positive regulation of peptidyl-serine phosphorylation	-8.09243
GO:0061045	negative regulation of wound healing	-8.07562
GO:0019932	second-messenger-mediated signaling	-8.0718
GO:0002687	positive regulation of leukocyte migration	-8.06702
GO:0048638	regulation of developmental growth	-8.06507
GO:0006164	purine nucleotide biosynthetic process	-8.065
GO:0090218	positive regulation of lipid kinase activity	-8.06
GO:1904994	regulation of leukocyte adhesion to vascular endothelial cell	-8.06
GO:2001171	positive regulation of ATP biosynthetic process	-8.06
GO:0051607	defense response to virus	-8.05868
GO:0042743	hydrogen peroxide metabolic process	-8.05741
GO:0010720	positive regulation of cell development	-8.05095
GO:0046886	positive regulation of hormone biosynthetic process	-8.04866
GO:0005975	carbohydrate metabolic process	-8.03118
GO:0030193	regulation of blood coagulation	-8.02787
GO:0002440	production of molecular mediator of immune response	-8.00806
GO:1901617	organic hydroxy compound biosynthetic process	-7.99547
GO:0071277	cellular response to calcium ion	-7.98076
GO:1900046	regulation of hemostasis	-7.98076
GO:0032885	regulation of polysaccharide biosynthetic process	-7.97427

GO:0010212	response to ionizing radiation	-7.97177
GO:0033135	regulation of peptidyl-serine phosphorylation	-7.97177
GO:0071346	cellular response to interferon-gamma	-7.9569
GO:0034620	cellular response to unfolded protein	-7.94051
GO:0071385	cellular response to glucocorticoid stimulus	-7.93511
GO:1904996	positive regulation of leukocyte adhesion to vascular endothelial cell	-7.8847
GO:0051592	response to calcium ion	-7.87872
GO:0009168	purine ribonucleoside monophosphate biosynthetic process	-7.87734
GO:0001836	release of cytochrome c from mitochondria	-7.87568
GO:0061900	glial cell activation	-7.87568
GO:0031348	negative regulation of defense response	-7.87151
GO:0072522	purine-containing compound biosynthetic process	-7.85937
GO:0006875	cellular metal ion homeostasis	-7.85077
GO:0006970	response to osmotic stress	-7.84315
GO:0006163	purine nucleotide metabolic process	-7.82623
GO:0009127	purine nucleoside monophosphate biosynthetic process	-7.82511
GO:0006606	protein import into nucleus	-7.81786
GO:0033619	membrane protein proteolysis	-7.81734
GO:1902041	regulation of extrinsic apoptotic signaling pathway via death domain receptors	-7.81734
GO:0007077	mitotic nuclear envelope disassembly	-7.81717
GO:0071803	positive regulation of podosome assembly	-7.81717
GO:0043123	positive regulation of I-kappaB kinase/NF-kappaB signaling	-7.79923

GO:0051341	regulation of oxidoreductase activity	-7.79387
GO:0097530	granulocyte migration	-7.78778
GO:0051188	cofactor biosynthetic process	-7.76868
GO:0002719	negative regulation of cytokine production involved in immune response	-7.76242
GO:0045821	positive regulation of glycolytic process	-7.76242
GO:1902993	positive regulation of amyloid precursor protein catabolic process	-7.76242
GO:0002260	lymphocyte homeostasis	-7.76007
GO:0030888	regulation of B cell proliferation	-7.76007
GO:0032755	positive regulation of interleukin-6 production	-7.75435
GO:0050818	regulation of coagulation	-7.75435
GO:0090559	regulation of membrane permeability	-7.75435
GO:0009142	nucleoside triphosphate biosynthetic process	-7.74795
GO:0022408	negative regulation of cell-cell adhesion	-7.72254
GO:0045807	positive regulation of endocytosis	-7.69885
GO:0009895	negative regulation of catabolic process	-7.69731
GO:0010822	positive regulation of mitochondrion organization	-7.68776
GO:0030111	regulation of Wnt signaling pathway	-7.68106
GO:0043900	regulation of multi-organism process	-7.67514
GO:0070542	response to fatty acid	-7.66777
GO:0051193	regulation of cofactor metabolic process	-7.65305
GO:0070920	regulation of production of small RNA involved in gene silencing by RNA	-7.64588
GO:0050864	regulation of B cell activation	-7.6224

GO:0032612	interleukin-1 production	-7.61866
GO:0051928	positive regulation of calcium ion transport	-7.61866
GO:0050728	negative regulation of inflammatory response	-7.59773
GO:0032768	regulation of monooxygenase activity	-7.59421
GO:0034103	regulation of tissue remodeling	-7.58331
GO:1905897	regulation of response to endoplasmic reticulum stress	-7.58331
GO:0046688	response to copper ion	-7.58061
GO:0051603	proteolysis involved in cellular protein catabolic process	-7.55875
GO:0032675	regulation of interleukin-6 production	-7.55492
GO:0050777	negative regulation of immune response	-7.55492
GO:0006694	steroid biosynthetic process	-7.54882
GO:0032677	regulation of interleukin-8 production	-7.54184
GO:0046824	positive regulation of nucleocytoplasmic transport	-7.54081
GO:0019430	removal of superoxide radicals	-7.53459
GO:0030813	positive regulation of nucleotide catabolic process	-7.53459
GO:0051197	positive regulation of coenzyme metabolic process	-7.53459
GO:0009156	ribonucleoside monophosphate biosynthetic process	-7.52458
GO:0032881	regulation of polysaccharide metabolic process	-7.50804
GO:0032890	regulation of organic acid transport	-7.48831
GO:0043550	regulation of lipid kinase activity	-7.48831
GO:0045670	regulation of osteoclast differentiation	-7.48831
GO:2000134	negative regulation of G1/S transition of mitotic cell cycle	-7.4842

GO:0042391	regulation of membrane potential	-7.47247
GO:0006754	ATP biosynthetic process	-7.471
GO:0034341	response to interferon-gamma	-7.45268
GO:0071621	granulocyte chemotaxis	-7.45134
GO:0014002	astrocyte development	-7.43731
GO:0030900	forebrain development	-7.43013
GO:0045672	positive regulation of osteoclast differentiation	-7.42809
GO:0046885	regulation of hormone biosynthetic process	-7.42809
GO:0048143	astrocyte activation	-7.42809
GO:1903035	negative regulation of response to wounding	-7.42037
GO:0010508	positive regulation of autophagy	-7.41876
GO:0010952	positive regulation of peptidase activity	-7.40543
GO:1901990	regulation of mitotic cell cycle phase transition	-7.40197
GO:0010721	negative regulation of cell development	-7.38858
GO:0002576	platelet degranulation	-7.38647
GO:0002637	regulation of immunoglobulin production	-7.38587
GO:0048662	negative regulation of smooth muscle cell proliferation	-7.38587
GO:0000904	cell morphogenesis involved in differentiation	-7.38444
GO:0016098	monoterpenoid metabolic process	-7.38285
GO:0002221	pattern recognition receptor signaling pathway	-7.382
GO:0015711	organic anion transport	-7.36957
GO:0050768	negative regulation of neurogenesis	-7.36935

GO:0007093	mitotic cell cycle checkpoint	-7.36181
GO:0035967	cellular response to topologically incorrect protein	-7.36181
GO:2001169	regulation of ATP biosynthetic process	-7.34172
GO:0071695	anatomical structure maturation	-7.33498
GO:0051348	negative regulation of transferase activity	-7.33289
GO:0032770	positive regulation of monooxygenase activity	-7.32601
GO:0071450	cellular response to oxygen radical	-7.32601
GO:0071451	cellular response to superoxide	-7.32601
GO:0019218	regulation of steroid metabolic process	-7.32273
GO:0051271	negative regulation of cellular component movement	-7.30925
GO:0002819	regulation of adaptive immune response	-7.30834
GO:0009167	purine ribonucleoside monophosphate metabolic process	-7.30622
GO:0043255	regulation of carbohydrate biosynthetic process	-7.30306
GO:0050810	regulation of steroid biosynthetic process	-7.30306
GO:0022602	ovulation cycle process	-7.30095
GO:0030225	macrophage differentiation	-7.30095
GO:0042572	retinol metabolic process	-7.30095
GO:1902807	negative regulation of cell cycle G1/S phase transition	-7.29126
GO:0009124	nucleoside monophosphate biosynthetic process	-7.28959
GO:0042982	amyloid precursor protein metabolic process	-7.28668
GO:0030336	negative regulation of cell migration	-7.27368
GO:0009126	purine nucleoside monophosphate metabolic process	-7.25749

GO:0016125	sterol metabolic process	-7.25558
GO:0034349	glial cell apoptotic process	-7.24611
GO:0042531	positive regulation of tyrosine phosphorylation of STAT protein	-7.23825
GO:0034248	regulation of cellular amide metabolic process	-7.22947
GO:0051170	import into nucleus	-7.22947
GO:0001782	B cell homeostasis	-7.22798
GO:0042116	macrophage activation	-7.22705
GO:0071560	cellular response to transforming growth factor beta stimulus	-7.22265
GO:0006511	ubiquitin-dependent protein catabolic process	-7.22263
GO:0051966	regulation of synaptic transmission, glutamatergic	-7.19055
GO:0009206	purine ribonucleoside triphosphate biosynthetic process	-7.17776
GO:0032680	regulation of tumor necrosis factor production	-7.17776
GO:0050870	positive regulation of T cell activation	-7.1769
GO:0002758	innate immune response-activating signal transduction	-7.17234
GO:0030850	prostate gland development	-7.17093
GO:0042149	cellular response to glucose starvation	-7.17093
GO:0019941	modification-dependent protein catabolic process	-7.15699
GO:0000303	response to superoxide	-7.13372
GO:0035666	TRIF-dependent toll-like receptor signaling pathway	-7.13372
GO:0060055	angiogenesis involved in wound healing	-7.13372
GO:0040013	negative regulation of locomotion	-7.1333
GO:0031589	cell-substrate adhesion	-7.11422

GO:0001961	positive regulation of cytokine-mediated signaling pathway	-7.10812
GO:0042987	amyloid precursor protein catabolic process	-7.10812
GO:0071559	response to transforming growth factor beta	-7.10536
GO:0016241	regulation of macroautophagy	-7.10146
GO:1903555	regulation of tumor necrosis factor superfamily cytokine production	-7.10146
GO:0046323	glucose import	-7.09731
GO:0019372	lipoxygenase pathway	-7.08597
GO:0030397	membrane disassembly	-7.08597
GO:0032352	positive regulation of hormone metabolic process	-7.08597
GO:0051081	nuclear envelope disassembly	-7.08597
GO:0071801	regulation of podosome assembly	-7.08597
GO:0009161	ribonucleoside monophosphate metabolic process	-7.08297
GO:0002367	cytokine production involved in immune response	-7.07994
GO:0009135	purine nucleoside diphosphate metabolic process	-7.07829
GO:0009179	purine ribonucleoside diphosphate metabolic process	-7.07829
GO:0007178	transmembrane receptor protein serine/threonine kinase signaling pathway	-7.05192
GO:0010469	regulation of signaling receptor activity	-7.05141
GO:0032640	tumor necrosis factor production	-7.05141
GO:0050435	amyloid-beta metabolic process	-7.04669
GO:0000305	response to oxygen radical	-7.04295
GO:0043632	modification-dependent macromolecule catabolic process	-7.03865
GO:2000146	negative regulation of cell motility	-7.03648

GO:0009201	ribonucleoside triphosphate biosynthetic process	-7.02663
GO:0023061	signal release	-7.02422
GO:0030330	DNA damage response, signal transduction by p53 class mediator	-7.00872
GO:0032611	interleukin-1 beta production	-7.00872
GO:0014015	positive regulation of gliogenesis	-7.0068
GO:0046902	regulation of mitochondrial membrane permeability	-7.0068
GO:0009185	ribonucleoside diphosphate metabolic process	-6.9907
GO:0050999	regulation of nitric-oxide synthase activity	-6.98658
GO:0002699	positive regulation of immune effector process	-6.98153
GO:0044257	cellular protein catabolic process	-6.97486
GO:0051961	negative regulation of nervous system development	-6.96676
GO:0002274	myeloid leukocyte activation	-6.96466
GO:0051385	response to mineralocorticoid	-6.95542
GO:0009792	embryo development ending in birth or egg hatching	-6.95417
GO:0015732	prostaglandin transport	-6.93729
GO:0030540	female genitalia development	-6.93729
GO:1904355	positive regulation of telomere capping	-6.93729
GO:0042737	drug catabolic process	-6.93346
GO:0006986	response to unfolded protein	-6.92906
GO:0071706	tumor necrosis factor superfamily cytokine production	-6.92906
GO:0032731	positive regulation of interleukin-1 beta production	-6.92775
GO:0043392	negative regulation of DNA binding	-6.92775

GO:0097366	response to bronchodilator	-6.92775
GO:0071260	cellular response to mechanical stimulus	-6.91887
GO:0050769	positive regulation of neurogenesis	-6.90858
GO:0008286	insulin receptor signaling pathway	-6.90518
GO:0032388	positive regulation of intracellular transport	-6.8976
GO:1901987	regulation of cell cycle phase transition	-6.89589
GO:0046328	regulation of JNK cascade	-6.88119
GO:0006110	regulation of glycolytic process	-6.87583
GO:0043552	positive regulation of phosphatidylinositol 3-kinase activity	-6.87092
GO:1902003	regulation of amyloid-beta formation	-6.87092
GO:1904659	glucose transmembrane transport	-6.87066
GO:0030857	negative regulation of epithelial cell differentiation	-6.87015
GO:0031050	dsRNA processing	-6.87015
GO:0070918	production of small RNA involved in gene silencing by RNA	-6.87015
GO:0034655	nucleobase-containing compound catabolic process	-6.86068
GO:0009166	nucleotide catabolic process	-6.85748
GO:0034404	nucleobase-containing small molecule biosynthetic process	-6.85628
GO:0002831	regulation of response to biotic stimulus	-6.84927
GO:0019359	nicotinamide nucleotide biosynthetic process	-6.84927
GO:0019363	pyridine nucleotide biosynthetic process	-6.84927
GO:0032652	regulation of interleukin-1 production	-6.83702
GO:0046822	regulation of nucleocytoplasmic transport	-6.83702

GO:0008217	regulation of blood pressure	-6.83391
GO:0061138	morphogenesis of a branching epithelium	-6.83391
GO:0010833	telomere maintenance via telomere lengthening	-6.83338
GO:0071158	positive regulation of cell cycle arrest	-6.83338
GO:0051962	positive regulation of nervous system development	-6.81885
GO:0051353	positive regulation of oxidoreductase activity	-6.81371
GO:0002285	lymphocyte activation involved in immune response	-6.81048
GO:0016049	cell growth	-6.808
GO:0032963	collagen metabolic process	-6.80371
GO:2000278	regulation of DNA biosynthetic process	-6.80371
GO:0002756	MyD88-independent toll-like receptor signaling pathway	-6.78925
GO:0042311	vasodilation	-6.78925
GO:0071478	cellular response to radiation	-6.7872
GO:0002218	activation of innate immune response	-6.78627
GO:0009123	nucleoside monophosphate metabolic process	-6.78144
GO:0008645	hexose transmembrane transport	-6.77073
GO:0016101	diterpenoid metabolic process	-6.77073
GO:0072525	pyridine-containing compound biosynthetic process	-6.767
GO:0009152	purine ribonucleotide biosynthetic process	-6.75377
GO:0002718	regulation of cytokine production involved in immune response	-6.75021
GO:0030811	regulation of nucleotide catabolic process	-6.75021
GO:0035265	organ growth	-6.74106

GO:0006096	glycolytic process	-6.73807
GO:0009914	hormone transport	-6.72241
GO:1901292	nucleoside phosphate catabolic process	-6.7182
GO:1901030	positive regulation of mitochondrial outer membrane permeabilization involved in apoptotic signaling pathway	-6.71022
GO:0006757	ATP generation from ADP	-6.70573
GO:0015749	monosaccharide transmembrane transport	-6.70573
GO:0001541	ovarian follicle development	-6.70419
GO:0060760	positive regulation of response to cytokine stimulus	-6.70419
GO:0019362	pyridine nucleotide metabolic process	-6.69548
GO:0046496	nicotinamide nucleotide metabolic process	-6.69548
GO:0002822	regulation of adaptive immune response based on somatic recombination of immune receptors built from immunoglobulin superfamily domains	-6.68658
GO:0043500	muscle adaptation	-6.67371
GO:0060749	mammary gland alveolus development	-6.66852
GO:0061377	mammary gland lobule development	-6.66852
GO:1902176	negative regulation of oxidative stress-induced intrinsic apoptotic signaling pathway	-6.66852
GO:0043551	regulation of phosphatidylinositol 3-kinase activity	-6.65102
GO:1900408	negative regulation of cellular response to oxidative stress	-6.65102
GO:1903202	negative regulation of oxidative stress-induced cell death	-6.65102
GO:0007219	Notch signaling pathway	-6.65043
GO:0021700	developmental maturation	-6.64853
GO:0034219	carbohydrate transmembrane transport	-6.64199

GO:0006090	pyruvate metabolic process	-6.63396
GO:0002701	negative regulation of production of molecular mediator of immune response	-6.63369
GO:0042554	superoxide anion generation	-6.63369
GO:1901099	negative regulation of signal transduction in absence of ligand	-6.63369
GO:2001240	negative regulation of extrinsic apoptotic signaling pathway in absence of ligand	-6.63369
GO:0038093	Fc receptor signaling pathway	-6.61688
GO:0042866	pyruvate biosynthetic process	-6.61057
GO:0006636	unsaturated fatty acid biosynthetic process	-6.59886
GO:0045454	cell redox homeostasis	-6.59886
GO:0042509	regulation of tyrosine phosphorylation of STAT protein	-6.59039
GO:0072507	divalent inorganic cation homeostasis	-6.57705
GO:0048568	embryonic organ development	-6.57629
GO:0090322	regulation of superoxide metabolic process	-6.5595
GO:0009132	nucleoside diphosphate metabolic process	-6.55646
GO:0050729	positive regulation of inflammatory response	-6.55646
GO:0002065	columnar/cuboidal epithelial cell differentiation	-6.54861
GO:0032732	positive regulation of interleukin-1 production	-6.54767
GO:1902883	negative regulation of response to oxidative stress	-6.54767
GO:0071800	podosome assembly	-6.54617
GO:1902004	positive regulation of amyloid-beta formation	-6.54617
GO:0072524	pyridine-containing compound metabolic process	-6.54011
GO:0090316	positive regulation of intracellular protein transport	-6.531

GO:0009260	ribonucleotide biosynthetic process	-6.529
GO:0006469	negative regulation of protein kinase activity	-6.52121
GO:0001763	morphogenesis of a branching structure	-6.51843
GO:1901222	regulation of NIK/NF-kappaB signaling	-6.51807
GO:0009144	purine nucleoside triphosphate metabolic process	-6.50597
GO:1904356	regulation of telomere maintenance via telomere lengthening	-6.49742
GO:0005996	monosaccharide metabolic process	-6.49547
GO:0071392	cellular response to estradiol stimulus	-6.48751
GO:1905898	positive regulation of response to endoplasmic reticulum stress	-6.48751
GO:0007260	tyrosine phosphorylation of STAT protein	-6.4758
GO:0043470	regulation of carbohydrate catabolic process	-6.4758
GO:0043009	chordate embryonic development	-6.47247
GO:0046849	bone remodeling	-6.43856
GO:0051899	membrane depolarization	-6.43856
GO:0035966	response to topologically incorrect protein	-6.43293
GO:0061042	vascular wound healing	-6.43066
GO:1901991	negative regulation of mitotic cell cycle phase transition	-6.42778
GO:0014037	Schwann cell differentiation	-6.41761
GO:0034205	amyloid-beta formation	-6.41761
GO:0060557	positive regulation of vitamin D biosynthetic process	-6.41227
GO:0060559	positive regulation of calcidiol 1-monooxygenase activity	-6.41227
GO:0007179	transforming growth factor beta receptor signaling pathway	-6.41186

GO:0046661	male sex differentiation	-6.40638
GO:0006913	nucleocytoplasmic transport	-6.40166
GO:0031571	mitotic G1 DNA damage checkpoint	-6.39961
GO:0006805	xenobiotic metabolic process	-6.39865
GO:0046390	ribose phosphate biosynthetic process	-6.39647
GO:0007507	heart development	-6.39606
GO:0030217	T cell differentiation	-6.39103
GO:0017038	protein import	-6.3909
GO:0007088	regulation of mitotic nuclear division	-6.38198
GO:0006820	anion transport	-6.38186
GO:0000086	G2/M transition of mitotic cell cycle	-6.37278
GO:0046031	ADP metabolic process	-6.36946
GO:0051169	nuclear transport	-6.35771
GO:0044783	G1 DNA damage checkpoint	-6.35199
GO:0044819	mitotic G1/S transition checkpoint	-6.35199
GO:0045453	bone resorption	-6.35199
GO:0032350	regulation of hormone metabolic process	-6.34967
GO:0042307	positive regulation of protein import into nucleus	-6.34967
GO:1902991	regulation of amyloid precursor protein catabolic process	-6.34967
GO:0010948	negative regulation of cell cycle process	-6.32867
GO:0051900	regulation of mitochondrial depolarization	-6.32127
GO:0046717	acid secretion	-6.31186

GO:0031341	regulation of cell killing	-6.29401
GO:0097194	execution phase of apoptosis	-6.29401
GO:0030278	regulation of ossification	-6.28786
GO:0038095	Fc-epsilon receptor signaling pathway	-6.28603
GO:0042533	tumor necrosis factor biosynthetic process	-6.2836
GO:0042534	regulation of tumor necrosis factor biosynthetic process	-6.2836
GO:0071548	response to dexamethasone	-6.2836
GO:0006733	oxidoreduction coenzyme metabolic process	-6.2676
GO:0035249	synaptic transmission, glutamatergic	-6.25893
GO:0051196	regulation of coenzyme metabolic process	-6.25893
GO:0046034	ATP metabolic process	-6.25229
GO:0002676	regulation of chronic inflammatory response	-6.24672
GO:0051302	regulation of cell division	-6.23903
GO:0009141	nucleoside triphosphate metabolic process	-6.22853
GO:2001023	regulation of response to drug	-6.22426
GO:0001558	regulation of cell growth	-6.21934
GO:1904591	positive regulation of protein import	-6.2193
GO:0051043	regulation of membrane protein ectodomain proteolysis	-6.2174
GO:0060149	negative regulation of posttranscriptional gene silencing	-6.2174
GO:0060967	negative regulation of gene silencing by RNA	-6.2174
GO:1901522	positive regulation of transcription from RNA polymerase II promoter involved in cellular response to chemical stimulus	-6.2174
GO:19037	regulation of production of miRNAs involved in	-6.2174

98	gene silencing by miRNA		
GO:0010498	proteasomal protein catabolic process	-6.20677	
GO:0006165	nucleoside diphosphate phosphorylation	-6.19962	
GO:0043161	proteasome-mediated ubiquitin-dependent protein catabolic process	-6.19365	
GO:0033209	tumor necrosis factor-mediated signaling pathway	-6.19266	
GO:0032651	regulation of interleukin-1 beta production	-6.18999	
GO:0045833	negative regulation of lipid metabolic process	-6.18999	
GO:0071674	mononuclear cell migration	-6.18999	
GO:0016236	macroautophagy	-6.18981	
GO:0010810	regulation of cell-substrate adhesion	-6.18762	
GO:0045123	cellular extravasation	-6.16946	
GO:0045124	regulation of bone resorption	-6.15668	
GO:0000075	cell cycle checkpoint	-6.14827	
GO:0046939	nucleotide phosphorylation	-6.14493	
GO:0002460	adaptive immune response based on somatic recombination of immune receptors built from immunoglobulin superfamily domains	-6.14453	
GO:1902903	regulation of supramolecular fiber organization	-6.14453	
GO:0033673	negative regulation of kinase activity	-6.12583	
GO:0007004	telomere maintenance via telomerase	-6.12568	
GO:0019748	secondary metabolic process	-6.12568	
GO:0071230	cellular response to amino acid stimulus	-6.12568	
GO:0001906	cell killing		-6.12424
GO:00215	pallium development	-6.12424	

43		
GO:0036003	positive regulation of transcription from RNA polymerase II promoter in response to stress	-6.11853
GO:0051882	mitochondrial depolarization	-6.11853
GO:0050671	positive regulation of lymphocyte proliferation	-6.11793
GO:0045861	negative regulation of proteolysis	-6.10315
GO:0050680	negative regulation of epithelial cell proliferation	-6.10173
GO:1901031	regulation of response to reactive oxygen species	-6.09565
GO:1901988	negative regulation of cell cycle phase transition	-6.0918
GO:0030183	B cell differentiation	-6.09116
GO:0032946	positive regulation of mononuclear cell proliferation	-6.09116
GO:0043280	positive regulation of cysteine-type endopeptidase activity involved in apoptotic process	-6.09116
GO:0019722	calcium-mediated signaling	-6.09002
GO:0072503	cellular divalent inorganic cation homeostasis	-6.08993
GO:0015908	fatty acid transport	-6.08949
GO:0050715	positive regulation of cytokine secretion	-6.08261
GO:0090303	positive regulation of wound healing	-6.08261
GO:0043619	regulation of transcription from RNA polymerase II promoter in response to oxidative stress	-6.05293
GO:0051712	positive regulation of killing of cells of other organism	-6.05293
GO:0051235	maintenance of location	-6.05264
GO:0044839	cell cycle G2/M phase transition	-6.04131
GO:0006509	membrane protein ectodomain proteolysis	-6.03615
GO:00341	positive regulation of tissue remodeling	-6.03615

05		
GO:0035094	response to nicotine	-6.03615
GO:1901028	regulation of mitochondrial outer membrane permeabilization involved in apoptotic signaling pathway	-6.03615
GO:1901796	regulation of signal transduction by p53 class mediator	-6.03507
GO:0046883	regulation of hormone secretion	-6.02463
GO:0070498	interleukin-1-mediated signaling pathway	-6.02433
GO:0042535	positive regulation of tumor necrosis factor biosynthetic process	-6.02421
GO:0045619	regulation of lymphocyte differentiation	-6.01314
GO:0008584	male gonad development	-5.98623
GO:0043401	steroid hormone mediated signaling pathway	-5.98623
GO:0032309	icosanoid secretion	-5.9781
GO:0032570	response to progesterone	-5.9781
GO:0046546	development of primary male sexual characteristics	-5.96052
GO:0070227	lymphocyte apoptotic process	-5.95739
GO:0072401	signal transduction involved in DNA integrity checkpoint	-5.95739
GO:0072422	signal transduction involved in DNA damage checkpoint	-5.95739
GO:0051051	negative regulation of transport	-5.9536
GO:0060562	epithelial tube morphogenesis	-5.9491
GO:0002695	negative regulation of leukocyte activation	-5.94817
GO:0010950	positive regulation of endopeptidase activity	-5.94817
GO:0001783	B cell apoptotic process	-5.93404
GO:0002092	positive regulation of receptor internalization	-5.93404

GO:0070633	transepithelial transport	-5.93404
GO:1904353	regulation of telomere capping	-5.93404
GO:0006953	acute-phase response	-5.92143
GO:0014075	response to amine	-5.92143
GO:0048806	genitalia development	-5.92143
GO:0072395	signal transduction involved in cell cycle checkpoint	-5.91692
GO:0032990	cell part morphogenesis	-5.91553
GO:1990778	protein localization to cell periphery	-5.90553
GO:0001649	osteoblast differentiation	-5.90234
GO:0042368	vitamin D biosynthetic process	-5.87933
GO:0072659	protein localization to plasma membrane	-5.87768
GO:0043627	response to estrogen	-5.87705
GO:0001523	retinoid metabolic process	-5.86746
GO:0008593	regulation of Notch signaling pathway	-5.86746
GO:2001239	regulation of extrinsic apoptotic signaling pathway in absence of ligand	-5.86608
GO:0071322	cellular response to carbohydrate stimulus	-5.85973
GO:0006278	RNA-dependent DNA biosynthetic process	-5.83777
GO:0045444	fat cell differentiation	-5.82991
GO:0051783	regulation of nuclear division	-5.82182
GO:0009205	purine ribonucleoside triphosphate metabolic process	-5.8198
GO:0014038	regulation of Schwann cell differentiation	-5.81257
GO:0002686	negative regulation of leukocyte migration	-5.812

GO:0035196	production of miRNAs involved in gene silencing by miRNA	-5.812
GO:0090199	regulation of release of cytochrome c from mitochondria	-5.812
GO:1990090	cellular response to nerve growth factor stimulus	-5.812
GO:0006874	cellular calcium ion homeostasis	-5.80195
GO:0033157	regulation of intracellular protein transport	-5.77653
GO:0003012	muscle system process	-5.767
GO:0010575	positive regulation of vascular endothelial growth factor production	-5.76484
GO:0033598	mammary gland epithelial cell proliferation	-5.76484
GO:1900101	regulation of endoplasmic reticulum unfolded protein response	-5.76484
GO:1990776	response to angiotensin	-5.76484
GO:2000191	regulation of fatty acid transport	-5.76484
GO:0045580	regulation of T cell differentiation	-5.76204
GO:0046850	regulation of bone remodeling	-5.75912
GO:1903727	positive regulation of phospholipid metabolic process	-5.75912
GO:0099565	chemical synaptic transmission, postsynaptic	-5.74774
GO:0008643	carbohydrate transport	-5.73809
GO:0009199	ribonucleoside triphosphate metabolic process	-5.7359
GO:0000271	polysaccharide biosynthetic process	-5.72329
GO:0010827	regulation of glucose transmembrane transport	-5.72329
GO:0030656	regulation of vitamin metabolic process	-5.72213
GO:0061043	regulation of vascular wound healing	-5.72213
GO:0071287	cellular response to manganese ion	-5.72213

GO:1900103	positive regulation of endoplasmic reticulum unfolded protein response	-5.72213
GO:0035272	exocrine system development	-5.70741
GO:0038084	vascular endothelial growth factor signaling pathway	-5.70741
GO:0051972	regulation of telomerase activity	-5.70741
GO:0061028	establishment of endothelial barrier	-5.70741
GO:0042098	T cell proliferation	-5.70002
GO:0098693	regulation of synaptic vesicle cycle	-5.68967
GO:0032204	regulation of telomere maintenance	-5.68621
GO:0048678	response to axon injury	-5.68621
GO:1901224	positive regulation of NIK/NF-kappaB signaling	-5.68621
GO:0032800	receptor biosynthetic process	-5.68524
GO:0061564	axon development	-5.68237
GO:0001678	cellular glucose homeostasis	-5.66729
GO:0008203	cholesterol metabolic process	-5.66729
GO:0051250	negative regulation of lymphocyte activation	-5.66729
GO:2001056	positive regulation of cysteine-type endopeptidase activity	-5.66729
GO:0055074	calcium ion homeostasis	-5.66383
GO:0000186	activation of MAPKK activity	-5.65681
GO:0051205	protein insertion into membrane	-5.65681
GO:1990089	response to nerve growth factor	-5.65681
GO:0002224	toll-like receptor signaling pathway	-5.64405
GO:0002262	myeloid cell homeostasis	-5.64405

GO:0046942	carboxylic acid transport	-5.64023	
GO:0031623	receptor internalization	-5.63275	
GO:0015849	organic acid transport	-5.62675	
GO:0034644	cellular response to UV	-5.61357	
GO:0001844	protein insertion into mitochondrial membrane involved in apoptotic signaling pathway	-5.60864	
GO:0010800	positive regulation of peptidyl-threonine phosphorylation	-5.60864	
GO:0043457	regulation of cellular respiration	-5.60864	
GO:0032210	regulation of telomere maintenance via telomerase	-5.60727	
GO:0006352	DNA-templated transcription, initiation	-5.60416	
GO:1902652	secondary alcohol metabolic process	-5.59806	
GO:0050890	cognition		-5.58503
GO:0030213	hyaluronan biosynthetic process	-5.5785	
GO:0032310	prostaglandin secretion	-5.5785	
GO:0051709	regulation of killing of cells of other organism	-5.5785	
GO:1903799	negative regulation of production of miRNAs involved in gene silencing by miRNA	-5.5785	
GO:0022617	extracellular matrix disassembly	-5.57799	
GO:0031145	anaphase-promoting complex-dependent catabolic process	-5.57799	
GO:1902930	regulation of alcohol biosynthetic process	-5.57799	
GO:1903036	positive regulation of response to wounding	-5.57799	
GO:0007569	cell aging		-5.57692
GO:0045664	regulation of neuron differentiation	-5.5657	

GO:0010639	negative regulation of organelle organization	-5.55138
GO:0071549	cellular response to dexamethasone stimulus	-5.53484
GO:0010876	lipid localization	-5.52744
GO:0019318	hexose metabolic process	-5.52102
GO:0045926	negative regulation of growth	-5.52102
GO:0032655	regulation of interleukin-12 production	-5.51124
GO:0097345	mitochondrial outer membrane permeabilization	-5.51124
GO:0034767	positive regulation of ion transmembrane transport	-5.50808
GO:0009150	purine ribonucleotide metabolic process	-5.50712
GO:2000106	regulation of leukocyte apoptotic process	-5.47405
GO:0006977	DNA damage response, signal transduction by p53 class mediator resulting in cell cycle arrest	-5.46466
GO:0010951	negative regulation of endopeptidase activity	-5.4559
GO:0090287	regulation of cellular response to growth factor stimulus	-5.45406
GO:0042362	fat-soluble vitamin biosynthetic process	-5.4463
GO:0051044	positive regulation of membrane protein ectodomain proteolysis	-5.4463
GO:0090594	inflammatory response to wounding	-5.4463
GO:2001028	positive regulation of endothelial cell chemotaxis	-5.4463
GO:0001764	neuron migration	-5.44227
GO:0046165	alcohol biosynthetic process	-5.44227
GO:0009108	coenzyme biosynthetic process	-5.43981
GO:0007420	brain development	-5.43254
GO:0002703	regulation of leukocyte mediated immunity	-5.43197

GO:0030520	intracellular estrogen receptor signaling pathway	-5.419
GO:0032615	interleukin-12 production	-5.419
GO:0071398	cellular response to fatty acid	-5.419
GO:0072431	signal transduction involved in mitotic G1 DNA damage checkpoint	-5.419
GO:1902400	intracellular signal transduction involved in G1 DNA damage checkpoint	-5.419
GO:1905517	macrophage migration	-5.419
GO:0002677	negative regulation of chronic inflammatory response	-5.41698
GO:0014805	smooth muscle adaptation	-5.41698
GO:0032747	positive regulation of interleukin-23 production	-5.41698
GO:0046136	positive regulation of vitamin metabolic process	-5.41698
GO:1990962	drug transport across blood-brain barrier	-5.41698
GO:0002688	regulation of leukocyte chemotaxis	-5.41566
GO:0046889	positive regulation of lipid biosynthetic process	-5.40698
GO:0045736	negative regulation of cyclin-dependent protein serine/threonine kinase activity	-5.39487
GO:0046434	organophosphate catabolic process	-5.39198
GO:0001952	regulation of cell-matrix adhesion	-5.38964
GO:0006869	lipid transport	-5.37924
GO:0042129	regulation of T cell proliferation	-5.37784
GO:0021537	telencephalon development	-5.37618
GO:0002090	regulation of receptor internalization	-5.37421
GO:0007566	embryo implantation	-5.37421
GO:0035306	positive regulation of dephosphorylation	-5.37421

GO:0034605	cellular response to heat	-5.36386
GO:0016051	carbohydrate biosynthetic process	-5.35921
GO:0009259	ribonucleotide metabolic process	-5.35823
GO:0034250	positive regulation of cellular amide metabolic process	-5.35665
GO:0042058	regulation of epidermal growth factor receptor signaling pathway	-5.3416
GO:0042306	regulation of protein import into nucleus	-5.33028
GO:0072413	signal transduction involved in mitotic cell cycle checkpoint	-5.33028
GO:1902402	signal transduction involved in mitotic DNA damage checkpoint	-5.33028
GO:1902403	signal transduction involved in mitotic DNA integrity checkpoint	-5.33028
GO:0032212	positive regulation of telomere maintenance via telomerase	-5.32837
GO:0051204	protein insertion into mitochondrial membrane	-5.32837
GO:1904030	negative regulation of cyclin-dependent protein kinase activity	-5.32837
GO:0002070	epithelial cell maturation	-5.32385
GO:0034116	positive regulation of heterotypic cell-cell adhesion	-5.32385
GO:0051770	positive regulation of nitric-oxide synthase biosynthetic process	-5.32385
GO:1901550	regulation of endothelial cell development	-5.32385
GO:1903140	regulation of establishment of endothelial barrier	-5.32385
GO:0051701	interaction with host	-5.32343
GO:0002250	adaptive immune response	-5.32183
GO:0032874	positive regulation of stress-activated MAPK cascade	-5.31472
GO:1905952	regulation of lipid localization	-5.31472
GO:0046879	hormone secretion	-5.31383

GO:0033273	response to vitamin	-5.30952
GO:0060333	interferon-gamma-mediated signaling pathway	-5.30952
GO:0061098	positive regulation of protein tyrosine kinase activity	-5.28716
GO:0032760	positive regulation of tumor necrosis factor production	-5.27783
GO:0106027	neuron projection organization	-5.27783
GO:0070304	positive regulation of stress-activated protein kinase signaling cascade	-5.27336
GO:0006691	leukotriene metabolic process	-5.264
GO:0032735	positive regulation of interleukin-12 production	-5.264
GO:0060251	regulation of glial cell proliferation	-5.264
GO:0071312	cellular response to alkaloid	-5.264
GO:0015718	monocarboxylic acid transport	-5.25289
GO:0032088	negative regulation of NF-kappaB transcription factor activity	-5.24653
GO:0061097	regulation of protein tyrosine kinase activity	-5.24653
GO:0046324	regulation of glucose import	-5.24483
GO:1902110	positive regulation of mitochondrial membrane permeability involved in apoptotic process	-5.24483
GO:0010466	negative regulation of peptidase activity	-5.23716
GO:0034308	primary alcohol metabolic process	-5.2156
GO:1901992	positive regulation of mitotic cell cycle phase transition	-5.2156
GO:1903557	positive regulation of tumor necrosis factor superfamily cytokine production	-5.2156
GO:0010803	regulation of tumor necrosis factor-mediated signaling pathway	-5.20327
GO:1904589	regulation of protein import	-5.20327
GO:0001893	maternal placenta development	-5.20165

GO:0045742	positive regulation of epidermal growth factor receptor signaling pathway	-5.20165
GO:0051973	positive regulation of telomerase activity	-5.20165
GO:0097435	supramolecular fiber organization	-5.18825
GO:1903725	regulation of phospholipid metabolic process	-5.18504
GO:0002768	immune response-regulating cell surface receptor signaling pathway	-5.18329
GO:0030324	lung development	-5.17235
GO:0019693	ribose phosphate metabolic process	-5.16759
GO:0032368	regulation of lipid transport	-5.16567
GO:0120035	regulation of plasma membrane bounded cell projection organization	-5.16365
GO:1902686	mitochondrial outer membrane permeabilization involved in programmed cell death	-5.16244
GO:0002532	production of molecular mediator involved in inflammatory response	-5.15484
GO:0010975	regulation of neuron projection development	-5.15319
GO:0015980	energy derivation by oxidation of organic compounds	-5.14751
GO:0042692	muscle cell differentiation	-5.14687
GO:0071333	cellular response to glucose stimulus	-5.14184
GO:0030212	hyaluronan metabolic process	-5.14118
GO:1904358	positive regulation of telomere maintenance via telomere lengthening	-5.14118
GO:1901184	regulation of ERBB signaling pathway	-5.12499
GO:0002066	columnar/cuboidal epithelial cell development	-5.12233
GO:0070141	response to UV-A	-5.11831
GO:0140014	mitotic nuclear division	-5.10356
GO:00359	response to muscle stretch	-5.1032

94		
GO:0050764	regulation of phagocytosis	-5.09548
GO:0071331	cellular response to hexose stimulus	-5.0948
GO:1903038	negative regulation of leukocyte cell-cell adhesion	-5.0948
GO:0030323	respiratory tube development	-5.0939
GO:0010573	vascular endothelial growth factor production	-5.08291
GO:0035794	positive regulation of mitochondrial membrane permeability	-5.08291
GO:0031344	regulation of cell projection organization	-5.08265
GO:0030224	monocyte differentiation	-5.08249
GO:0032733	positive regulation of interleukin-10 production	-5.08249
GO:1901186	positive regulation of ERBB signaling pathway	-5.08249
GO:1903131	mononuclear cell differentiation	-5.08249
GO:2001252	positive regulation of chromosome organization	-5.0746
GO:0046330	positive regulation of JNK cascade	-5.07157
GO:0071326	cellular response to monosaccharide stimulus	-5.07157
GO:0007160	cell-matrix adhesion	-5.06697
GO:0016579	protein deubiquitination	-5.06017
GO:0030177	positive regulation of Wnt signaling pathway	-5.05543
GO:0043433	negative regulation of DNA-binding transcription factor activity	-5.05543
GO:1902108	regulation of mitochondrial membrane permeability involved in apoptotic process	-5.04416
GO:0051640	organelle localization	-5.03477
GO:0048812	neuron projection morphogenesis	-5.02804

GO:0072655	establishment of protein localization to mitochondrion	-5.02569
GO:0007431	salivary gland development	-5.02548
GO:0033146	regulation of intracellular estrogen receptor signaling pathway	-5.02548
GO:0046326	positive regulation of glucose import	-5.02548
GO:0044773	mitotic DNA damage checkpoint	-5.00896
GO:1900015	regulation of cytokine production involved in inflammatory response	-5.00605
GO:1905710	positive regulation of membrane permeability	-5.00605
GO:0010544	negative regulation of platelet activation	-5.00303
GO:0032930	positive regulation of superoxide anion generation	-5.00303
GO:0045780	positive regulation of bone resorption	-5.00303
GO:0046852	positive regulation of bone remodeling	-5.00303
GO:0051023	regulation of immunoglobulin secretion	-5.00303
GO:0070989	oxidative demethylation	-5.00303
GO:0043502	regulation of muscle adaptation	-4.98076
GO:0007006	mitochondrial membrane organization	-4.98055
GO:0045740	positive regulation of DNA replication	-4.97007
GO:0050691	regulation of defense response to virus by host	-4.97007
GO:2000273	positive regulation of signaling receptor activity	-4.97007
GO:0010812	negative regulation of cell-substrate adhesion	-4.96857
GO:0016239	positive regulation of macroautophagy	-4.96857
GO:0006732	coenzyme metabolic process	-4.96186
GO:0045598	regulation of fat cell differentiation	-4.95825

GO:0030593	neutrophil chemotaxis	-4.95287
GO:0060079	excitatory postsynaptic potential	-4.95287
GO:0006367	transcription initiation from RNA polymerase II promoter	-4.9429
GO:0060078	regulation of postsynaptic membrane potential	-4.93614
GO:0070585	protein localization to mitochondrion	-4.93614
GO:0032649	regulation of interferon-gamma production	-4.92529
GO:2000060	positive regulation of ubiquitin-dependent protein catabolic process	-4.92529
GO:0051346	negative regulation of hydrolase activity	-4.9207
GO:0120039	plasma membrane bounded cell projection morphogenesis	-4.91175
GO:0002544	chronic inflammatory response	-4.90861
GO:0002902	regulation of B cell apoptotic process	-4.90861
GO:0019373	epoxygenase P450 pathway	-4.90861
GO:0032303	regulation of icosanoid secretion	-4.90861
GO:0051412	response to corticosterone	-4.90861
GO:0051767	nitric-oxide synthase biosynthetic process	-4.90861
GO:0051769	regulation of nitric-oxide synthase biosynthetic process	-4.90861
GO:0060965	negative regulation of gene silencing by miRNA	-4.90861
GO:1900409	positive regulation of cellular response to oxidative stress	-4.90861
GO:0003015	heart process	-4.90571
GO:0060828	regulation of canonical Wnt signaling pathway	-4.90571
GO:0045621	positive regulation of lymphocyte differentiation	-4.898
GO:0000226	microtubule cytoskeleton organization	-4.89725

GO:0002534	cytokine production involved in inflammatory response	-4.89542
GO:0071242	cellular response to ammonium ion	-4.89542
GO:0072347	response to anesthetic	-4.89542
GO:1903320	regulation of protein modification by small protein conjugation or removal	-4.89115
GO:0043902	positive regulation of multi-organism process	-4.88819
GO:0048858	cell projection morphogenesis	-4.87912
GO:0051146	striated muscle cell differentiation	-4.87839
GO:0002933	lipid hydroxylation	-4.87762
GO:0009820	alkaloid metabolic process	-4.87762
GO:0031394	positive regulation of prostaglandin biosynthetic process	-4.87762
GO:0031622	positive regulation of fever generation	-4.87762
GO:0032627	interleukin-23 production	-4.87762
GO:0032667	regulation of interleukin-23 production	-4.87762
GO:0060558	regulation of calcidiol 1-monooxygenase activity	-4.87762
GO:0120041	positive regulation of macrophage proliferation	-4.87762
GO:2001300	lipoxin metabolic process	-4.87762
GO:2001301	lipoxin biosynthetic process	-4.87762
GO:0055123	digestive system development	-4.87083
GO:1904064	positive regulation of cation transmembrane transport	-4.87083
GO:0048246	macrophage chemotaxis	-4.86371
GO:0060969	negative regulation of gene silencing	-4.86371
GO:0090279	regulation of calcium ion import	-4.86371

GO:1904037	positive regulation of epithelial cell apoptotic process	-4.86371
GO:0010675	regulation of cellular carbohydrate metabolic process	-4.8494
GO:0030260	entry into host cell	-4.8494
GO:0044409	entry into host	-4.8494
GO:0051806	entry into cell of other organism involved in symbiotic interaction	-4.8494
GO:0051828	entry into other organism involved in symbiotic interaction	-4.82814
GO:0070646	protein modification by small protein removal	-4.8244
GO:0009110	vitamin biosynthetic process	-4.81934
GO:0010893	positive regulation of steroid biosynthetic process	-4.81934
GO:0060716	labyrinthine layer blood vessel development	-4.81934
GO:0071498	cellular response to fluid shear stress	-4.81934
GO:0090280	positive regulation of calcium ion import	-4.81934
GO:0090330	regulation of platelet aggregation	-4.81934
GO:0044774	mitotic DNA integrity checkpoint	-4.81788
GO:0033044	regulation of chromosome organization	-4.81414
GO:0150077	regulation of neuroinflammatory response	-4.8126
GO:1903364	positive regulation of cellular protein catabolic process	-4.80704
GO:0002286	T cell activation involved in immune response	-4.79173
GO:0021761	limbic system development	-4.79173
GO:0000077	DNA damage checkpoint	-4.78611
GO:0034765	regulation of ion transmembrane transport	-4.78049
GO:1901989	positive regulation of cell cycle phase transition	-4.76585

GO:0002040	sprouting angiogenesis	-4.76436
GO:1905521	regulation of macrophage migration	-4.76279
GO:0031670	cellular response to nutrient	-4.75586
GO:0016311	dephosphorylation	-4.75112
GO:0016052	carbohydrate catabolic process	-4.74709
GO:0048259	regulation of receptor-mediated endocytosis	-4.74024
GO:0032928	regulation of superoxide anion generation	-4.73468
GO:0042359	vitamin D metabolic process	-4.73468
GO:0048305	immunoglobulin secretion	-4.73468
GO:0051000	positive regulation of nitric-oxide synthase activity	-4.73468
GO:1902884	positive regulation of response to oxidative stress	-4.73468
GO:1901137	carbohydrate derivative biosynthetic process	-4.72868
GO:0045685	regulation of glial cell differentiation	-4.72229
GO:0010828	positive regulation of glucose transmembrane transport	-4.71422
GO:0060443	mammary gland morphogenesis	-4.71422
GO:0009755	hormone-mediated signaling pathway	-4.71285
GO:0048754	branching morphogenesis of an epithelial tube	-4.70394
GO:0050808	synapse organization	-4.69043
GO:0005976	polysaccharide metabolic process	-4.68979
GO:0062014	negative regulation of small molecule metabolic process	-4.68979
GO:0022604	regulation of cell morphogenesis	-4.68349
GO:0090257	regulation of muscle system process	-4.68076

GO:0060541	respiratory system development	-4.67901
GO:0001955	blood vessel maturation	-4.67586
GO:0002246	wound healing involved in inflammatory response	-4.67586
GO:0031620	regulation of fever generation	-4.67586
GO:0033129	positive regulation of histone phosphorylation	-4.67586
GO:0035655	interleukin-18-mediated signaling pathway	-4.67586
GO:0071351	cellular response to interleukin-18	-4.67586
GO:0120040	regulation of macrophage proliferation	-4.67586
GO:1900125	regulation of hyaluronan biosynthetic process	-4.67586
GO:0043330	response to exogenous dsRNA	-4.66682
GO:0048538	thymus development	-4.66682
GO:0007422	peripheral nervous system development	-4.65661
GO:0061418	regulation of transcription from RNA polymerase II promoter in response to hypoxia	-4.65661
GO:0010288	response to lead ion	-4.6542
GO:0035743	CD4-positive, alpha-beta T cell cytokine production	-4.6542
GO:0032609	interferon-gamma production	-4.64034
GO:0050688	regulation of defense response to virus	-4.62449
GO:0007611	learning or memory	-4.62318
GO:0042180	cellular ketone metabolic process	-4.62318
GO:1903362	regulation of cellular protein catabolic process	-4.62318
GO:0030574	collagen catabolic process	-4.62055
GO:0035722	interleukin-12-mediated signaling pathway	-4.62055

GO:0046620	regulation of organ growth	-4.61599
GO:1902905	positive regulation of supramolecular fiber organization	-4.6125
GO:0035303	regulation of dephosphorylation	-4.59611
GO:0031016	pancreas development	-4.59282
GO:0099601	regulation of neurotransmitter receptor activity	-4.59282
GO:0050707	regulation of cytokine secretion	-4.59187
GO:0000723	telomere maintenance	-4.58519
GO:0031570	DNA integrity checkpoint	-4.58519
GO:2000058	regulation of ubiquitin-dependent protein catabolic process	-4.58519
GO:0042738	exogenous drug catabolic process	-4.57751
GO:0050995	negative regulation of lipid catabolic process	-4.57751
GO:0010799	regulation of peptidyl-threonine phosphorylation	-4.57536
GO:0030518	intracellular steroid hormone receptor signaling pathway	-4.56799
GO:0035821	modification of morphology or physiology of other organism	-4.5659
GO:0002377	immunoglobulin production	-4.5636
GO:0048863	stem cell differentiation	-4.55271
GO:0090276	regulation of peptide hormone secretion	-4.53146
GO:0002067	glandular epithelial cell differentiation	-4.53119
GO:0003254	regulation of membrane depolarization	-4.53119
GO:0045601	regulation of endothelial cell differentiation	-4.53119
GO:0060711	labyrinthine layer development	-4.53119
GO:0071349	cellular response to interleukin-12	-4.53119

GO:2000107	negative regulation of leukocyte apoptotic process	-4.53119
GO:0021766	hippocampus development	-4.5308
GO:0006006	glucose metabolic process	-4.51553
GO:1903305	regulation of regulated secretory pathway	-4.50885
GO:0032647	regulation of interferon-alpha production	-4.50428
GO:0046697	decidualization	-4.50428
GO:0060571	morphogenesis of an epithelial fold	-4.50428
GO:1904385	cellular response to angiotensin	-4.50428
GO:2001026	regulation of endothelial cell chemotaxis	-4.50428
GO:0051121	hepoxilin metabolic process	-4.50212
GO:0051122	hepoxilin biosynthetic process	-4.50212
GO:0060556	regulation of vitamin D biosynthetic process	-4.50212
GO:0097011	cellular response to granulocyte macrophage colony-stimulating factor stimulus	-4.50212
GO:0097012	response to granulocyte macrophage colony-stimulating factor	-4.50212
GO:1901857	positive regulation of cellular respiration	-4.50212
GO:2001280	positive regulation of unsaturated fatty acid biosynthetic process	-4.50212
GO:0010906	regulation of glucose metabolic process	-4.4977
GO:0021987	cerebral cortex development	-4.4977
GO:0001774	microglial cell activation	-4.48801
GO:0002269	leukocyte activation involved in inflammatory response	-4.48801
GO:0002833	positive regulation of response to biotic stimulus	-4.48801
GO:0014009	glial cell proliferation	-4.48801

GO:0030195	negative regulation of blood coagulation	-4.48801
GO:0070671	response to interleukin-12	-4.48801
GO:0042752	regulation of circadian rhythm	-4.47471
GO:1990266	neutrophil migration	-4.45193
GO:0031113	regulation of microtubule polymerization	-4.44578
GO:0032206	positive regulation of telomere maintenance	-4.44578
GO:0071354	cellular response to interleukin-6	-4.44578
GO:0071675	regulation of mononuclear cell migration	-4.44578
GO:1900047	negative regulation of hemostasis	-4.44578
GO:0034502	protein localization to chromosome	-4.44093
GO:0060740	prostate gland epithelium morphogenesis	-4.43422
GO:1900739	regulation of protein insertion into mitochondrial membrane involved in apoptotic signaling pathway	-4.43422
GO:1900740	positive regulation of protein insertion into mitochondrial membrane involved in apoptotic signaling pathway	-4.43422
GO:1905523	positive regulation of macrophage migration	-4.43422
GO:0045927	positive regulation of growth	-4.42984
GO:0032092	positive regulation of protein binding	-4.41178
GO:0070509	calcium ion import	-4.41178
GO:0032479	regulation of type I interferon production	-4.40702
GO:0006998	nuclear envelope organization	-4.40445
GO:0006898	receptor-mediated endocytosis	-4.39848
GO:0048667	cell morphogenesis involved in neuron differentiation	-4.36864

GO:0001963	synaptic transmission, dopaminergic	-4.36707
GO:0010971	positive regulation of G2/M transition of mitotic cell cycle	-4.36707
GO:0032607	interferon-alpha production	-4.36707
GO:0034114	regulation of heterotypic cell-cell adhesion	-4.36707
GO:0034698	response to gonadotropin	-4.36707
GO:0042501	serine phosphorylation of STAT protein	-4.36707
GO:2000353	positive regulation of endothelial cell apoptotic process	-4.36707
GO:0043331	response to dsRNA	-4.36399
GO:0048260	positive regulation of receptor-mediated endocytosis	-4.36399
GO:0032606	type I interferon production	-4.36292
GO:0050954	sensory perception of mechanical stimulus	-4.36246
GO:0010507	negative regulation of autophagy	-4.3546
GO:0034637	cellular carbohydrate biosynthetic process	-4.3546
GO:0048013	ephrin receptor signaling pathway	-4.3546
GO:0051781	positive regulation of cell division	-4.3546
GO:0060070	canonical Wnt signaling pathway	-4.35192
GO:0001660	fever generation	-4.34957
GO:0032308	positive regulation of prostaglandin secretion	-4.34957
GO:0034350	regulation of glial cell apoptotic process	-4.34957
GO:0036500	ATF6-mediated unfolded protein response	-4.34957
GO:0045348	positive regulation of MHC class II biosynthetic process	-4.34957
GO:0051541	elastin metabolic process	-4.34957

GO:0051901	positive regulation of mitochondrial depolarization	-4.34957
GO:0060068	vagina development	-4.34957
GO:0070424	regulation of nucleotide-binding oligomerization domain containing signaling pathway	-4.34957
GO:0097267	omega-hydroxylase P450 pathway	-4.34957
GO:0032200	telomere organization	-4.34472
GO:1903052	positive regulation of proteolysis involved in cellular protein catabolic process	-4.34116
GO:0017157	regulation of exocytosis	-4.33097
GO:1903050	regulation of proteolysis involved in cellular protein catabolic process	-4.33097
GO:0006749	glutathione metabolic process	-4.32437
GO:0010524	positive regulation of calcium ion transport into cytosol	-4.32437
GO:0019058	viral life cycle	-4.31745
GO:0032970	regulation of actin filament-based process	-4.30526
GO:0030194	positive regulation of blood coagulation	-4.30262
GO:0060512	prostate gland morphogenesis	-4.30262
GO:0071280	cellular response to copper ion	-4.30262
GO:1900048	positive regulation of hemostasis	-4.30262
GO:1902932	positive regulation of alcohol biosynthetic process	-4.30262
GO:0001822	kidney development	-4.29898
GO:0007411	axon guidance	-4.29898
GO:0034101	erythrocyte homeostasis	-4.29824
GO:0071482	cellular response to light stimulus	-4.29824
GO:0090150	establishment of protein localization to membrane	-4.29468

GO:0097485	neuron projection guidance	-4.2862
GO:0016233	telomere capping	-4.28555
GO:0050819	negative regulation of coagulation	-4.28555
GO:0070741	response to interleukin-6	-4.28555
GO:0006919	activation of cysteine-type endopeptidase activity involved in apoptotic process	-4.27155
GO:0048469	cell maturation	-4.25778
GO:0015893	drug transport	-4.25751
GO:0050663	cytokine secretion	-4.25607
GO:0070228	regulation of lymphocyte apoptotic process	-4.24751
GO:0050820	positive regulation of coagulation	-4.24065
GO:0050901	leukocyte tethering or rolling	-4.24065
GO:1904062	regulation of cation transmembrane transport	-4.23848
GO:0050796	regulation of insulin secretion	-4.22381
GO:0045582	positive regulation of T cell differentiation	-4.2179
GO:0002275	myeloid cell activation involved in immune response	-4.21734
GO:0006527	arginine catabolic process	-4.21362
GO:0031652	positive regulation of heat generation	-4.21362
GO:0032306	regulation of prostaglandin secretion	-4.21362
GO:0033197	response to vitamin E	-4.21362
GO:0060525	prostate glandular acinus development	-4.21362
GO:0070673	response to interleukin-18	-4.21362
GO:0007409	axonogenesis	-4.21266

GO:0045840	positive regulation of mitotic nuclear division	-4.21021
GO:0002705	positive regulation of leukocyte mediated immunity	-4.19418
GO:0072001	renal system development	-4.18596
GO:0001516	prostaglandin biosynthetic process	-4.18099
GO:0005979	regulation of glycogen biosynthetic process	-4.18099
GO:0007176	regulation of epidermal growth factor-activated receptor activity	-4.18099
GO:0010758	regulation of macrophage chemotaxis	-4.18099
GO:0010962	regulation of glucan biosynthetic process	-4.18099
GO:0034110	regulation of homotypic cell-cell adhesion	-4.18099
GO:0046457	prostanoid biosynthetic process	-4.18099
GO:0071480	cellular response to gamma radiation	-4.18099
GO:1902751	positive regulation of cell cycle G2/M phase transition	-4.18099
GO:1903205	regulation of hydrogen peroxide-induced cell death	-4.18099
GO:2001025	positive regulation of response to drug	-4.18099
GO:0006766	vitamin metabolic process	-4.1739
GO:0055076	transition metal ion homeostasis	-4.1739
GO:0022900	electron transport chain	-4.17367
GO:1903793	positive regulation of anion transport	-4.17363
GO:0002444	myeloid leukocyte mediated immunity	-4.16733
GO:0044262	cellular carbohydrate metabolic process	-4.16143
GO:1901888	regulation of cell junction assembly	-4.13985
GO:0051480	regulation of cytosolic calcium ion concentration	-4.13984

GO:0017001	antibiotic catabolic process	-4.13775
GO:0014044	Schwann cell development	-4.12348
GO:0045940	positive regulation of steroid metabolic process	-4.12348
GO:0002690	positive regulation of leukocyte chemotaxis	-4.11445
GO:0010660	regulation of muscle cell apoptotic process	-4.11445
GO:0002433	immune response-regulating cell surface receptor signaling pathway involved in phagocytosis	-4.11409
GO:0035304	regulation of protein dephosphorylation	-4.11409
GO:0038096	Fc-gamma receptor signaling pathway involved in phagocytosis	-4.11409
GO:0032956	regulation of actin cytoskeleton organization	-4.10765
GO:0032653	regulation of interleukin-10 production	-4.10253
GO:0043388	positive regulation of DNA binding	-4.10253
GO:0006983	ER overload response	-4.09103
GO:0031392	regulation of prostaglandin biosynthetic process	-4.09103
GO:0043696	dedifferentiation	-4.09103
GO:0043697	cell dedifferentiation	-4.09103
GO:0060736	prostate gland growth	-4.09103
GO:0060742	epithelial cell differentiation involved in prostate gland development	-4.09103
GO:0061517	macrophage proliferation	-4.09103
GO:0072584	caveolin-mediated endocytosis	-4.09103
GO:1904181	positive regulation of membrane depolarization	-4.09103
GO:0030308	negative regulation of cell growth	-4.07623
GO:00705	regulation of microtubule cytoskeleton	-4.07623

07	organization	
GO:0035767	endothelial cell chemotaxis	-4.06797
GO:0050869	negative regulation of B cell activation	-4.06797
GO:0051968	positive regulation of synaptic transmission, glutamatergic	-4.06797
GO:0071353	cellular response to interleukin-4	-4.06797
GO:0097421	liver regeneration	-4.06797
GO:0010574	regulation of vascular endothelial growth factor production	-4.06796
GO:0031343	positive regulation of cell killing	-4.06796
GO:1903078	positive regulation of protein localization to plasma membrane	-4.06796
GO:0048167	regulation of synaptic plasticity	-4.06035
GO:0038094	Fc-gamma receptor signaling pathway	-4.05575
GO:0002449	lymphocyte mediated immunity	-4.04429
GO:0010657	muscle cell apoptotic process	-4.01578
GO:0050848	regulation of calcium-mediated signaling	-4.01578
GO:0001975	response to amphetamine	-4.01434
GO:0002861	regulation of inflammatory response to antigenic stimulus	-4.01434
GO:0010039	response to iron ion	-4.01434
GO:0042573	retinoic acid metabolic process	-4.01434
GO:2001038	regulation of cellular response to drug	-4.01434
GO:0021885	forebrain cell migration	-4.00068
GO:0032613	interleukin-10 production	-4.00068
GO:0034113	heterotypic cell-cell adhesion	-4.00068

GO:0070059	intrinsic apoptotic signaling pathway in response to endoplasmic reticulum stress	-4.00068	
GO:0002431	Fc receptor mediated stimulatory signaling pathway	-3.99883	
GO:0007612	learning		-3.99883
GO:0007423	sensory organ development	-3.99789	
GO:0002702	positive regulation of production of molecular mediator of immune response	-3.99181	
GO:0031650	regulation of heat generation	-3.97944	
GO:0032725	positive regulation of granulocyte macrophage colony-stimulating factor production	-3.97944	
GO:0033127	regulation of histone phosphorylation	-3.97944	
GO:0045741	positive regulation of epidermal growth factor-activated receptor activity	-3.97944	
GO:0051988	regulation of attachment of spindle microtubules to kinetochore	-3.97944	
GO:0060340	positive regulation of type I interferon-mediated signaling pathway	-3.97944	
GO:0072683	T cell extravasation	-3.97944	
GO:0002437	inflammatory response to antigenic stimulus	-3.96792	
GO:0030431	sleep		-3.96247
GO:0032148	activation of protein kinase B activity	-3.96247	
GO:0033198	response to ATP	-3.96247	
GO:0036474	cell death in response to hydrogen peroxide	-3.96247	
GO:0043276	anoikis		-3.96247
GO:0045907	positive regulation of vasoconstriction	-3.96247	
GO:0071901	negative regulation of protein serine/threonine kinase activity	-3.94326	
GO:00075	excretion		-

88			3.93573
GO:0031640	killing of cells of other organism	-3.93573	
GO:0044364	disruption of cells of other organism	-3.93573	
GO:0010389	regulation of G2/M transition of mitotic cell cycle	-3.92171	
GO:0007435	salivary gland morphogenesis	-3.91225	
GO:0060674	placenta blood vessel development	-3.91225	
GO:0070670	response to interleukin-4	-3.91225	
GO:1903670	regulation of sprouting angiogenesis	-3.90694	
GO:0099504	synaptic vesicle cycle	-3.90677	
GO:0030072	peptide hormone secretion	-3.90433	
GO:0034330	cell junction organization	-3.89341	
GO:0050871	positive regulation of B cell activation	-3.889	
GO:0014854	response to inactivity	-3.87705	
GO:0070431	nucleotide-binding oligomerization domain containing 2 signaling pathway	-3.87705	
GO:2001279	regulation of unsaturated fatty acid biosynthetic process	-3.87705	
GO:0006417	regulation of translation	-3.8744	
GO:0050918	positive chemotaxis	-3.87297	
GO:0072678	T cell migration	-3.87297	
GO:1903672	positive regulation of sprouting angiogenesis	-3.87297	
GO:0033762	response to glucagon	-3.86359	
GO:0070873	regulation of glycogen metabolic process	-3.86359	
GO:2000310	regulation of NMDA receptor activity	-3.86359	

GO:0051983	regulation of chromosome segregation	-3.85345
GO:0007204	positive regulation of cytosolic calcium ion concentration	-3.84928
GO:0050766	positive regulation of phagocytosis	-3.84238
GO:1904377	positive regulation of protein localization to cell periphery	-3.84238
GO:0002706	regulation of lymphocyte mediated immunity	-3.83598
GO:0030038	contractile actin filament bundle assembly	-3.83126
GO:0043149	stress fiber assembly	-3.83126
GO:0061448	connective tissue development	-3.81667
GO:0097242	amyloid-beta clearance	-3.81638
GO:0032370	positive regulation of lipid transport	-3.81228
GO:0001909	leukocyte mediated cytotoxicity	-3.80929
GO:0051048	negative regulation of secretion	-3.79036
GO:0051187	cofactor catabolic process	-3.78266
GO:0006000	fructose metabolic process	-3.78249
GO:0010763	positive regulation of fibroblast migration	-3.78249
GO:0032645	regulation of granulocyte macrophage colony-stimulating factor production	-3.78249
GO:0045346	regulation of MHC class II biosynthetic process	-3.78249
GO:0048308	organelle inheritance	-3.78249
GO:0048313	Golgi inheritance	-3.78249
GO:0090557	establishment of endothelial intestinal barrier	-3.78249
GO:0010742	macrophage derived foam cell differentiation	-3.77056
GO:0043029	T cell homeostasis	-3.77056

GO:0048009	insulin-like growth factor receptor signaling pathway	-3.77056	
GO:0090077	foam cell differentiation	-3.77056	
GO:0006839	mitochondrial transport	-3.76786	
GO:0043271	negative regulation of ion transport	-3.76715	
GO:0060968	regulation of gene silencing	-3.76715	
GO:0042303	molting cycle	-3.76605	
GO:0042633	hair cycle		-3.76605
GO:0031396	regulation of protein ubiquitination	-3.76209	
GO:0043043	peptide biosynthetic process	-3.7609	
GO:0014823	response to activity	-3.75351	
GO:0051785	positive regulation of nuclear division	-3.75351	
GO:0035051	cardiocyte differentiation	-3.75026	
GO:0006275	regulation of DNA replication	-3.74476	
GO:0030073	insulin secretion	-3.73415	
GO:0030258	lipid modification	-3.73184	
GO:0043299	leukocyte degranulation	-3.73165	
GO:0032660	regulation of interleukin-17 production	-3.72605	
GO:0042092	type 2 immune response	-3.72605	
GO:0043114	regulation of vascular permeability	-3.72605	
GO:0060416	response to growth hormone	-3.72605	
GO:1904706	negative regulation of vascular smooth muscle cell proliferation	-3.72605	
GO:19044	positive regulation of calcium ion	-3.72483	

27	transmembrane transport	
GO:0051656	establishment of organelle localization	-3.71603
GO:0016485	protein processing	-3.71052
GO:0046916	cellular transition metal ion homeostasis	-3.70284
GO:0016570	histone modification	-3.70064
GO:0048285	organelle fission	-3.70064
GO:1903747	regulation of establishment of protein localization to mitochondrion	-3.69658
GO:0032604	granulocyte macrophage colony-stimulating factor production	-3.69466
GO:0045342	MHC class II biosynthetic process	-3.69466
GO:0045725	positive regulation of glycogen biosynthetic process	-3.69466
GO:0071732	cellular response to nitric oxide	-3.69466
GO:0031214	biomineral tissue development	-3.68394
GO:0006730	one-carbon metabolic process	-3.68278
GO:0007257	activation of JUN kinase activity	-3.68278
GO:0032892	positive regulation of organic acid transport	-3.68278
GO:0045746	negative regulation of Notch signaling pathway	-3.68278
GO:0051281	positive regulation of release of sequestered calcium ion into cytosol	-3.68278
GO:1902749	regulation of cell cycle G2/M phase transition	-3.67921
GO:0032481	positive regulation of type I interferon production	-3.66876
GO:0043507	positive regulation of JUN kinase activity	-3.66876
GO:1902117	positive regulation of organelle assembly	-3.66876
GO:1903828	negative regulation of cellular protein localization	-3.66175

GO:0007517	muscle organ development	-3.65343
GO:0030282	bone mineralization	-3.64151
GO:0072676	lymphocyte migration	-3.64151
GO:0030890	positive regulation of B cell proliferation	-3.64068
GO:0016042	lipid catabolic process	-3.6285
GO:0031330	negative regulation of cellular catabolic process	-3.62668
GO:0050868	negative regulation of T cell activation	-3.62147
GO:0002429	immune response-activating cell surface receptor signaling pathway	-3.61741
GO:0009064	glutamine family amino acid metabolic process	-3.61438
GO:1900076	regulation of cellular response to insulin stimulus	-3.61438
GO:0030889	negative regulation of B cell proliferation	-3.61268
GO:0030949	positive regulation of vascular endothelial growth factor receptor signaling pathway	-3.61268
GO:0031649	heat generation	-3.61268
GO:0032305	positive regulation of icosanoid secretion	-3.61268
GO:0033599	regulation of mammary gland epithelial cell proliferation	-3.61268
GO:0035729	cellular response to hepatocyte growth factor stimulus	-3.61268
GO:0045986	negative regulation of smooth muscle contraction	-3.61268
GO:0050665	hydrogen peroxide biosynthetic process	-3.61268
GO:0070875	positive regulation of glycogen metabolic process	-3.61268
GO:1903209	positive regulation of oxidative stress-induced cell death	-3.61268
GO:0006836	neurotransmitter transport	-3.60389
GO:0097479	synaptic vesicle localization	-3.6037

GO:0010907	positive regulation of glucose metabolic process	-3.59969
GO:0033048	negative regulation of mitotic sister chromatid segregation	-3.59969
GO:0032886	regulation of microtubule-based process	-3.59906
GO:0016569	covalent chromatin modification	-3.5929
GO:0002526	acute inflammatory response	-3.58596
GO:0060147	regulation of posttranscriptional gene silencing	-3.58197
GO:0060966	regulation of gene silencing by RNA	-3.58197
GO:0045665	negative regulation of neuron differentiation	-3.57293
GO:0060047	heart contraction	-3.57007
GO:0007052	mitotic spindle organization	-3.56251
GO:0033143	regulation of intracellular steroid hormone receptor signaling pathway	-3.56158
GO:0046785	microtubule polymerization	-3.56158
GO:0072332	intrinsic apoptotic signaling pathway by p53 class mediator	-3.56158
GO:0001953	negative regulation of cell-matrix adhesion	-3.55977
GO:0002369	T cell cytokine production	-3.55977
GO:0015701	bicarbonate transport	-3.55977
GO:0018904	ether metabolic process	-3.53585
GO:0033194	response to hydroperoxide	-3.53585
GO:0045187	regulation of circadian sleep/wake cycle, sleep	-3.53585
GO:0061298	retina vasculature development in camera-type eye	-3.53585
GO:0071318	cellular response to ATP	-3.53585
GO:2000641	regulation of early endosome to late endosome transport	-3.53585

GO:2001170	negative regulation of ATP biosynthetic process	-3.53585
GO:0042246	tissue regeneration	-3.53575
GO:0060395	SMAD protein signal transduction	-3.53575
GO:1900034	regulation of cellular response to heat	-3.53575
GO:0099173	postsynapse organization	-3.52624
GO:0050807	regulation of synapse organization	-3.52151
GO:0032620	interleukin-17 production	-3.52086
GO:0033046	negative regulation of sister chromatid segregation	-3.52086
GO:0035307	positive regulation of protein dephosphorylation	-3.52086
GO:0045687	positive regulation of glial cell differentiation	-3.52086
GO:0000280	nuclear division	-3.51087
GO:0002312	B cell activation involved in immune response	-3.51029
GO:2000300	regulation of synaptic vesicle exocytosis	-3.51029
GO:0051495	positive regulation of cytoskeleton organization	-3.50882
GO:0002698	negative regulation of immune effector process	-3.50521
GO:0032271	regulation of protein polymerization	-3.49621
GO:0003014	renal system process	-3.48647
GO:0035270	endocrine system development	-3.48647
GO:0001974	blood vessel remodeling	-3.48291
GO:0006775	fat-soluble vitamin metabolic process	-3.48291
GO:0034198	cellular response to amino acid starvation	-3.48291
GO:0051985	negative regulation of chromosome segregation	-3.48291

GO:0002862	negative regulation of inflammatory response to antigenic stimulus	-3.46356
GO:0010759	positive regulation of macrophage chemotaxis	-3.46356
GO:0034393	positive regulation of smooth muscle cell apoptotic process	-3.46356
GO:0035728	response to hepatocyte growth factor	-3.46356
GO:0060252	positive regulation of glial cell proliferation	-3.46356
GO:1902170	cellular response to reactive nitrogen species	-3.46356
GO:0002446	neutrophil mediated immunity	-3.45001
GO:0002639	positive regulation of immunoglobulin production	-3.44588
GO:0005978	glycogen biosynthetic process	-3.44588
GO:0009250	glucan biosynthetic process	-3.44588
GO:0010761	fibroblast migration	-3.44588
GO:0010863	positive regulation of phospholipase C activity	-3.44588
GO:0001570	vasculogenesis	-3.436
GO:0001937	negative regulation of endothelial cell proliferation	-3.436
GO:0014855	striated muscle cell proliferation	-3.436
GO:0060560	developmental growth involved in morphogenesis	-3.43412
GO:0031647	regulation of protein stability	-3.42856
GO:0097061	dendritic spine organization	-3.41191
GO:0007157	heterophilic cell-cell adhesion via plasma membrane cell adhesion molecules	-3.40973
GO:0032480	negative regulation of type I interferon production	-3.40973
GO:0045776	negative regulation of blood pressure	-3.40973
GO:0070849	response to epidermal growth factor	-3.40973

GO:0006525	arginine metabolic process	-3.39532
GO:0007252	I-kappaB phosphorylation	-3.39532
GO:0032495	response to muramyl dipeptide	-3.39532
GO:0032727	positive regulation of interferon-alpha production	-3.39532
GO:0071731	response to nitric oxide	-3.39532
GO:2000193	positive regulation of fatty acid transport	-3.39532
GO:0090288	negative regulation of cellular response to growth factor stimulus	-3.39334
GO:2001257	regulation of cation channel activity	-3.39334
GO:0031110	regulation of microtubule polymerization or depolymerization	-3.38815
GO:0050803	regulation of synapse structure or activity	-3.38561
GO:0045667	regulation of osteoblast differentiation	-3.37757
GO:0014911	positive regulation of smooth muscle cell migration	-3.37442
GO:0042220	response to cocaine	-3.37442
GO:1900271	regulation of long-term synaptic potentiation	-3.37442
GO:1990928	response to amino acid starvation	-3.37442
GO:1903522	regulation of blood circulation	-3.36586
GO:0014910	regulation of smooth muscle cell migration	-3.3647
GO:0045445	myoblast differentiation	-3.3647
GO:0070664	negative regulation of leukocyte proliferation	-3.3647
GO:0097756	negative regulation of blood vessel diameter	-3.3647
GO:1902803	regulation of synaptic vesicle transport	-3.3647
GO:0060291	long-term synaptic potentiation	-3.34155

GO:0006692	prostanoid metabolic process	-3.33992
GO:0006693	prostaglandin metabolic process	-3.33992
GO:0007595	lactation	-3.33992
GO:0032663	regulation of interleukin-2 production	-3.33992
GO:0045058	T cell selection	-3.33992
GO:0048512	circadian behavior	-3.33992
GO:1900087	positive regulation of G1/S transition of mitotic cell cycle	-3.33992
GO:1900274	regulation of phospholipase C activity	-3.33992
GO:0016540	protein autoprocessing	-3.33072
GO:0017000	antibiotic biosynthetic process	-3.33072
GO:0030728	ovulation	-3.33072
GO:0030809	negative regulation of nucleotide biosynthetic process	-3.33072
GO:0043651	linoleic acid metabolic process	-3.33072
GO:0046716	muscle cell cellular homeostasis	-3.33072
GO:0060065	uterus development	-3.33072
GO:0090201	negative regulation of release of cytochrome c from mitochondria	-3.33072
GO:0090312	positive regulation of protein deacetylation	-3.33072
GO:1900372	negative regulation of purine nucleotide biosynthetic process	-3.33072
GO:1902414	protein localization to cell junction	-3.33072
GO:2000737	negative regulation of stem cell differentiation	-3.33072
GO:0070588	calcium ion transmembrane transport	-3.32493

GO:0045333	cellular respiration	-3.32287
GO:0032436	positive regulation of proteasomal ubiquitin-dependent protein catabolic process	-3.31871
GO:0070098	chemokine-mediated signaling pathway	-3.31871
GO:0046718	viral entry into host cell	-3.30815
GO:0048565	digestive tract development	-3.30815
GO:0007622	rhythmic behavior	-3.30618
GO:0097300	programmed necrotic cell death	-3.30618
GO:0043506	regulation of JUN kinase activity	-3.29616
GO:0010565	regulation of cellular ketone metabolic process	-3.29532
GO:0048598	embryonic morphogenesis	-3.27485
GO:0032273	positive regulation of protein polymerization	-3.27434
GO:0045540	regulation of cholesterol biosynthetic process	-3.27319
GO:0045646	regulation of erythrocyte differentiation	-3.27319
GO:0045912	negative regulation of carbohydrate metabolic process	-3.27319
GO:0106118	regulation of sterol biosynthetic process	-3.27319
GO:1903573	negative regulation of response to endoplasmic reticulum stress	-3.27319
GO:0000717	nucleotide-excision repair, DNA duplex unwinding	-3.26939
GO:0032700	negative regulation of interleukin-17 production	-3.26939
GO:0042749	regulation of circadian sleep/wake cycle	-3.26939
GO:0045655	regulation of monocyte differentiation	-3.26939
GO:0050802	circadian sleep/wake cycle, sleep	-3.26939
GO:0097306	cellular response to alcohol	-3.25192

GO:0045839	negative regulation of mitotic nuclear division	-3.2409
GO:0071622	regulation of granulocyte chemotaxis	-3.2409
GO:2000677	regulation of transcription regulatory region DNA binding	-3.2409
GO:0006644	phospholipid metabolic process	-3.23833
GO:0031058	positive regulation of histone modification	-3.23021
GO:0000715	nucleotide-excision repair, DNA damage recognition	-3.21104
GO:0031281	positive regulation of cyclase activity	-3.21104
GO:0035162	embryonic hemopoiesis	-3.21104
GO:0045723	positive regulation of fatty acid biosynthetic process	-3.21104
GO:1900017	positive regulation of cytokine production involved in inflammatory response	-3.21104
GO:1901032	negative regulation of response to reactive oxygen species	-3.21104
GO:1903077	negative regulation of protein localization to plasma membrane	-3.21104
GO:1903206	negative regulation of hydrogen peroxide-induced cell death	-3.21104
GO:2001039	negative regulation of cellular response to drug	-3.21104
GO:0045599	negative regulation of fat cell differentiation	-3.2093
GO:0045661	regulation of myoblast differentiation	-3.2093
GO:0014909	smooth muscle cell migration	-3.20877
GO:0045185	maintenance of protein location	-3.20877
GO:0051702	interaction with symbiont	-3.20877
GO:0051952	regulation of amine transport	-3.20877
GO:1905954	positive regulation of lipid localization	-3.20877
GO:0090092	regulation of transmembrane receptor protein serine/threonine kinase signaling pathway	-3.20115

GO:2000027	regulation of animal organ morphogenesis	-3.20115
GO:0007292	female gamete generation	-3.1923
GO:0014706	striated muscle tissue development	-3.19206
GO:0034329	cell junction assembly	-3.1791
GO:0009620	response to fungus	-3.17835
GO:0010043	response to zinc ion	-3.17835
GO:0032623	interleukin-2 production	-3.17835
GO:0071320	cellular response to cAMP	-3.17835
GO:0003073	regulation of systemic arterial blood pressure	-3.16667
GO:0060337	type I interferon signaling pathway	-3.16667
GO:0071357	cellular response to type I interferon	-3.16667
GO:0007626	locomotory behavior	-3.16267
GO:0010256	endomembrane system organization	-3.16095
GO:0106106	cold-induced thermogenesis	-3.16043
GO:0120161	regulation of cold-induced thermogenesis	-3.16043
GO:0050851	antigen receptor-mediated signaling pathway	-3.15829
GO:0006297	nucleotide-excision repair, DNA gap filling	-3.15539
GO:0044321	response to leptin	-3.15539
GO:0051195	negative regulation of cofactor metabolic process	-3.15539
GO:0060396	growth hormone receptor signaling pathway	-3.15539
GO:0060444	branching involved in mammary gland duct morphogenesis	-3.15539
GO:1901889	negative regulation of cell junction assembly	-3.15539

GO:2000637	positive regulation of gene silencing by miRNA	-3.15539
GO:0002931	response to ischemia	-3.14803
GO:0008347	glial cell migration	-3.14803
GO:0046631	alpha-beta T cell activation	-3.12908
GO:0007589	body fluid secretion	-3.12557
GO:0046634	regulation of alpha-beta T cell activation	-3.12557
GO:1990868	response to chemokine	-3.12557
GO:1990869	cellular response to chemokine	-3.12557
GO:0016447	somatic recombination of immunoglobulin gene segments	-3.11832
GO:0031295	T cell costimulation	-3.11832
GO:0051588	regulation of neurotransmitter transport	-3.1136
GO:1902850	microtubule cytoskeleton organization involved in mitosis	-3.1136
GO:0030036	actin cytoskeleton organization	-3.1115
GO:0032412	regulation of ion transmembrane transporter activity	-3.10362
GO:0009404	toxin metabolic process	-3.10221
GO:0022410	circadian sleep/wake cycle process	-3.10221
GO:0045662	negative regulation of myoblast differentiation	-3.10221
GO:0050927	positive regulation of positive chemotaxis	-3.10221
GO:0060148	positive regulation of posttranscriptional gene silencing	-3.10221
GO:0061436	establishment of skin barrier	-3.10221
GO:0071378	cellular response to growth hormone stimulus	-3.10221
GO:1904376	negative regulation of protein localization to cell periphery	-3.10221

GO:2000679	positive regulation of transcription regulatory region DNA binding	-3.10221
GO:0031644	regulation of neurological system process	-3.09824
GO:2001251	negative regulation of chromosome organization	-3.09824
GO:0045747	positive regulation of Notch signaling pathway	-3.08919
GO:0048016	inositol phosphate-mediated signaling	-3.08919
GO:0034340	response to type I interferon	-3.08543
GO:0015850	organic hydroxy compound transport	-3.07204
GO:0090263	positive regulation of canonical Wnt signaling pathway	-3.06789
GO:0031294	lymphocyte costimulation	-3.06063
GO:1903749	positive regulation of establishment of protein localization to mitochondrion	-3.06063
GO:0051604	protein maturation	-3.05903
GO:0007276	gamete generation	-3.05887
GO:0031333	negative regulation of protein complex assembly	-3.05289
GO:1903169	regulation of calcium ion transmembrane transport	-3.05289
GO:0002068	glandular epithelial cell development	-3.0513
GO:0006309	apoptotic DNA fragmentation	-3.0513
GO:0006706	steroid catabolic process	-3.0513
GO:0035902	response to immobilization stress	-3.0513
GO:0042832	defense response to protozoan	-3.0513
GO:0050926	regulation of positive chemotaxis	-3.0513
GO:0070911	global genome nucleotide-excision repair	-3.0513
GO:1903649	regulation of cytoplasmic transport	-3.0513

GO:0007215	glutamate receptor signaling pathway	-3.04622
GO:0010522	regulation of calcium ion transport into cytosol	-3.04622
GO:0015837	amine transport	-3.04622
GO:0019217	regulation of fatty acid metabolic process	-3.04622
GO:0042102	positive regulation of T cell proliferation	-3.04622
GO:0002886	regulation of myeloid leukocyte mediated immunity	-3.03261
GO:0060043	regulation of cardiac muscle cell proliferation	-3.03261
GO:0055024	regulation of cardiac muscle tissue development	-3.02695
GO:0060537	muscle tissue development	-3.02687
GO:0019229	regulation of vasoconstriction	-3.00512
GO:0022029	telencephalon cell migration	-3.00512
GO:0050994	regulation of lipid catabolic process	-3.00512
GO:0051784	negative regulation of nuclear division	-3.00512
GO:0061077	chaperone-mediated protein folding	-3.00512
GO:1902808	positive regulation of cell cycle G1/S phase transition	-3.00512
GO:0001562	response to protozoan	-3.00249
GO:0002825	regulation of T-helper 1 type immune response	-3.00249
GO:0009065	glutamine family amino acid catabolic process	-3.00249
GO:0009651	response to salt stress	-3.00249
GO:1900120	regulation of receptor binding	-3.00249
GO:1903203	regulation of oxidative stress-induced neuron death	-3.00249
GO:1904357	negative regulation of telomere maintenance via telomere lengthening	-3.00249

GO:0006260	DNA replication	-3.00006
GO:0043312	neutrophil degranulation	-2.99846
GO:1990845	adaptive thermogenesis	-2.99407
GO:0022898	regulation of transmembrane transporter activity	-2.98997
GO:0008360	regulation of cell shape	-2.97965
GO:0033013	tetrapyrrole metabolic process	-2.97814
GO:0002283	neutrophil activation involved in immune response	-2.97709
GO:0110053	regulation of actin filament organization	-2.96992
GO:0022011	myelination in peripheral nervous system	-2.95561
GO:0032292	peripheral nervous system axon ensheathment	-2.95561
GO:0033561	regulation of water loss via skin	-2.95561
GO:0042745	circadian sleep/wake cycle	-2.95561
GO:0044331	cell-cell adhesion mediated by cadherin	-2.95561
GO:0045932	negative regulation of muscle contraction	-2.95561
GO:0090200	positive regulation of release of cytochrome c from mitochondria	-2.95561
GO:1903579	negative regulation of ATP metabolic process	-2.95561
GO:0002456	T cell mediated immunity	-2.95199
GO:0014812	muscle cell migration	-2.95199
GO:0032414	positive regulation of ion transmembrane transporter activity	-2.95199
GO:0034446	substrate adhesion-dependent cell spreading	-2.95199
GO:0046632	alpha-beta T cell differentiation	-2.95199
GO:0046928	regulation of neurotransmitter secretion	-2.95199

GO:1905269	positive regulation of chromatin organization	-2.95199	
GO:0002294	CD4-positive, alpha-beta T cell differentiation involved in immune response	-2.95165	
GO:0010518	positive regulation of phospholipase activity	-2.95165	
GO:0010676	positive regulation of cellular carbohydrate metabolic process	-2.95165	
GO:0048645	animal organ formation	-2.95165	
GO:0055025	positive regulation of cardiac muscle tissue development	-2.95165	
GO:0055081	anion homeostasis	-2.95165	
GO:2001244	positive regulation of intrinsic apoptotic signaling pathway	-2.95165	
GO:0055067	monovalent inorganic cation homeostasis	-2.95114	
GO:0060402	calcium ion transport into cytosol	-2.95114	
GO:1903707	negative regulation of hemopoiesis	-2.95114	
GO:0006412	translation	-2.93426	
GO:0097164	ammonium ion metabolic process	-2.93177	
GO:0002287	alpha-beta T cell activation involved in immune response	-2.92563	
GO:0002293	alpha-beta T cell differentiation involved in immune response	-2.92563	
GO:0046622	positive regulation of organ growth	-2.92563	
GO:0070527	platelet aggregation	-2.92563	
GO:0051017	actin filament bundle assembly	-2.92305	
GO:0006294	nucleotide-excision repair, preincision complex assembly	-2.91052	
GO:0007263	nitric oxide mediated signal transduction	-2.91052	
GO:0008209	androgen metabolic process	-2.91052	
GO:0019835	cytolysis		-2.9105

		2
GO:0046637	regulation of alpha-beta T cell differentiation	-2.90007
GO:0051898	negative regulation of protein kinase B signaling	-2.90007
GO:1900449	regulation of glutamate receptor signaling pathway	-2.90007
GO:2000401	regulation of lymphocyte migration	-2.90007
GO:0018958	phenol-containing compound metabolic process	-2.89788
GO:0042119	neutrophil activation	-2.89339
GO:0007229	integrin-mediated signaling pathway	-2.88023
GO:0015696	ammonium transport	-2.88023
GO:1901800	positive regulation of proteasomal protein catabolic process	-2.88023
GO:0032729	positive regulation of interferon-gamma production	-2.87495
GO:0051926	negative regulation of calcium ion transport	-2.87495
GO:0070265	necrotic cell death	-2.87495
GO:1905953	negative regulation of lipid localization	-2.87495
GO:0061572	actin filament bundle organization	-2.86813
GO:0031116	positive regulation of microtubule polymerization	-2.8671
GO:0036475	neuron death in response to oxidative stress	-2.8671
GO:0070168	negative regulation of biomineral tissue development	-2.8671
GO:0034440	lipid oxidation	-2.86277
GO:1901605	alpha-amino acid metabolic process	-2.85291
GO:0036230	granulocyte activation	-2.85258
GO:0016445	somatic diversification of immunoglobulins	-2.85027

GO:0090181	regulation of cholesterol metabolic process	-2.85027
GO:0098586	cellular response to virus	-2.85027
GO:1905818	regulation of chromosome separation	-2.85027
GO:2000378	negative regulation of reactive oxygen species metabolic process	-2.85027
GO:0007264	small GTPase mediated signal transduction	-2.84585
GO:0006289	nucleotide-excision repair	-2.84548
GO:0032409	regulation of transporter activity	-2.84399
GO:0046486	glycerolipid metabolic process	-2.84245
GO:0017158	regulation of calcium ion-dependent exocytosis	-2.82838
GO:1903510	mucopolysaccharide metabolic process	-2.82838
GO:0007015	actin filament organization	-2.82771
GO:0006879	cellular iron ion homeostasis	-2.826
GO:0036498	IRE1-mediated unfolded protein response	-2.826
GO:0046626	regulation of insulin receptor signaling pathway	-2.826
GO:0002675	positive regulation of acute inflammatory response	-2.82523
GO:0002828	regulation of type 2 immune response	-2.82523
GO:0010954	positive regulation of protein processing	-2.82523
GO:0061082	myeloid leukocyte cytokine production	-2.82523
GO:0070229	negative regulation of lymphocyte apoptotic process	-2.82523
GO:0090075	relaxation of muscle	-2.82523
GO:1900543	negative regulation of purine nucleotide metabolic process	-2.82523
GO:1900745	positive regulation of p38MAPK cascade	-2.82523

GO:1902230	negative regulation of intrinsic apoptotic signaling pathway in response to DNA damage	-2.82523
GO:1905476	negative regulation of protein localization to membrane	-2.82523
GO:0032411	positive regulation of transporter activity	-2.81145
GO:0002562	somatic diversification of immune receptors via germline recombination within a single locus	-2.80214
GO:0006625	protein targeting to peroxisome	-2.80214
GO:0016444	somatic cell DNA recombination	-2.80214
GO:0071479	cellular response to ionizing radiation	-2.80214
GO:0072662	protein localization to peroxisome	-2.80214
GO:0072663	establishment of protein localization to peroxisome	-2.80214
GO:0090307	mitotic spindle assembly	-2.80214
GO:0048640	negative regulation of developmental growth	-2.7947
GO:0002724	regulation of T cell cytokine production	-2.78481
GO:0002888	positive regulation of myeloid leukocyte mediated immunity	-2.78481
GO:0010743	regulation of macrophage derived foam cell differentiation	-2.78481
GO:0032673	regulation of interleukin-4 production	-2.78481
GO:0042744	hydrogen peroxide catabolic process	-2.78481
GO:0045980	negative regulation of nucleotide metabolic process	-2.78481
GO:0060603	mammary gland duct morphogenesis	-2.78481
GO:1902235	regulation of endoplasmic reticulum stress-induced intrinsic apoptotic signaling pathway	-2.78481
GO:0043574	peroxisomal transport	-2.77867
GO:0060038	cardiac muscle cell proliferation	-2.77867
GO:0060964	regulation of gene silencing by miRNA	-2.77811

GO:0030307	positive regulation of cell growth	-2.77581
GO:0002244	hematopoietic progenitor cell differentiation	-2.76299
GO:0051258	protein polymerization	-2.75197
GO:0060401	cytosolic calcium ion transport	-2.75027
GO:0035587	purinergic receptor signaling pathway	-2.74576
GO:1903319	positive regulation of protein maturation	-2.74576
GO:0051209	release of sequestered calcium ion into cytosol	-2.74544
GO:0051817	modification of morphology or physiology of other organism involved in symbiotic interaction	-2.74544
GO:0002292	T cell differentiation involved in immune response	-2.73287
GO:0033627	cell adhesion mediated by integrin	-2.73287
GO:0050795	regulation of behavior	-2.73287
GO:0050891	multicellular organismal water homeostasis	-2.73287
GO:0031109	microtubule polymerization or depolymerization	-2.72935
GO:0051283	negative regulation of sequestering of calcium ion	-2.72935
GO:0007269	neurotransmitter secretion	-2.72509
GO:0045216	cell-cell junction organization	-2.72509
GO:0099643	signal release from synapse	-2.72509
GO:2000736	regulation of stem cell differentiation	-2.71341
GO:0033047	regulation of mitotic sister chromatid segregation	-2.71051
GO:0033692	cellular polysaccharide biosynthetic process	-2.71051
GO:0070988	demethylation	-2.71051
GO:0000737	DNA catabolic process, endonucleolytic	-2.70797

GO:0002691	regulation of cellular extravasation	-2.70797
GO:0007094	mitotic spindle assembly checkpoint	-2.70797
GO:0008608	attachment of spindle microtubules to kinetochore	-2.70797
GO:0010762	regulation of fibroblast migration	-2.70797
GO:0030851	granulocyte differentiation	-2.70797
GO:0031112	positive regulation of microtubule polymerization or depolymerization	-2.70797
GO:0031577	spindle checkpoint	-2.70797
GO:0035633	maintenance of permeability of blood-brain barrier	-2.70797
GO:0071173	spindle assembly checkpoint	-2.70797
GO:0071174	mitotic spindle checkpoint	-2.70797
GO:2001024	negative regulation of response to drug	-2.70797
GO:0002792	negative regulation of peptide secretion	-2.69763
GO:0016079	synaptic vesicle exocytosis	-2.69763
GO:0031398	positive regulation of protein ubiquitination	-2.69763
GO:0051282	regulation of sequestering of calcium ion	-2.69763
GO:0002709	regulation of T cell mediated immunity	-2.6885
GO:0005977	glycogen metabolic process	-2.6885
GO:0010611	regulation of cardiac muscle hypertrophy	-2.6885
GO:0030218	erythrocyte differentiation	-2.682
GO:0010769	regulation of cell morphogenesis involved in differentiation	-2.6725
GO:0000083	regulation of transcription involved in G1/S transition of mitotic cell cycle	-2.67138
GO:0030262	apoptotic nuclear changes	-2.67138

GO:0030947	regulation of vascular endothelial growth factor receptor signaling pathway	-2.67138
GO:0032228	regulation of synaptic transmission, GABAergic	-2.67138
GO:0032633	interleukin-4 production	-2.67138
GO:0034390	smooth muscle cell apoptotic process	-2.67138
GO:0034391	regulation of smooth muscle cell apoptotic process	-2.67138
GO:0043304	regulation of mast cell degranulation	-2.67138
GO:0045648	positive regulation of erythrocyte differentiation	-2.67138
GO:0060338	regulation of type I interferon-mediated signaling pathway	-2.67138
GO:0070884	regulation of calcineurin-NFAT signaling cascade	-2.67138
GO:0071868	cellular response to monoamine stimulus	-2.67138
GO:0071870	cellular response to catecholamine stimulus	-2.67138
GO:0098801	regulation of renal system process	-2.67138
GO:0006073	cellular glucan metabolic process	-2.66683
GO:0010517	regulation of phospholipase activity	-2.66683
GO:0044042	glucan metabolic process	-2.66683
GO:0048675	axon extension	-2.66653
GO:0032535	regulation of cellular component size	-2.66582
GO:0001654	eye development	-2.65822
GO:0051208	sequestering of calcium ion	-2.6512
GO:0001910	regulation of leukocyte mediated cytotoxicity	-2.64549
GO:0050805	negative regulation of synaptic transmission	-2.64549
GO:0008361	regulation of cell size	-2.63965

GO:0070613	regulation of protein processing	-2.63965
GO:0003382	epithelial cell morphogenesis	-2.63593
GO:0032205	negative regulation of telomere maintenance	-2.63593
GO:0033006	regulation of mast cell activation involved in immune response	-2.63593
GO:0043368	positive T cell selection	-2.63593
GO:0045841	negative regulation of mitotic metaphase/anaphase transition	-2.63593
GO:0071634	regulation of transforming growth factor beta production	-2.63593
GO:0106056	regulation of calcineurin-mediated signaling	-2.63593
GO:0150063	visual system development	-2.62807
GO:0006695	cholesterol biosynthetic process	-2.62447
GO:0008306	associative learning	-2.62447
GO:0014743	regulation of muscle hypertrophy	-2.62447
GO:0033077	T cell differentiation in thymus	-2.62447
GO:0060193	positive regulation of lipase activity	-2.62447
GO:1902653	secondary alcohol biosynthetic process	-2.62447
GO:1903524	positive regulation of blood circulation	-2.62447
GO:0016358	dendrite development	-2.62385
GO:0007416	synapse assembly	-2.61598
GO:1903317	regulation of protein maturation	-2.61598
GO:1903531	negative regulation of secretion by cell	-2.61598
GO:0006081	cellular aldehyde metabolic process	-2.60376
GO:0032720	negative regulation of tumor necrosis factor production	-2.60376

GO:0006296	nucleotide-excision repair, DNA incision, 5'-to lesion	-2.60153
GO:0006471	protein ADP-ribosylation	-2.60153
GO:0006921	cellular component disassembly involved in execution phase of apoptosis	-2.60153
GO:0043243	positive regulation of protein complex disassembly	-2.60153
GO:0090050	positive regulation of cell migration involved in sprouting angiogenesis	-2.60153
GO:1902100	negative regulation of metaphase/anaphase transition of cell cycle	-2.60153
GO:0050770	regulation of axonogenesis	-2.59263
GO:0048880	sensory system development	-2.58365
GO:0042310	vasoconstriction	-2.58336
GO:0043367	CD4-positive, alpha-beta T cell differentiation	-2.58336
GO:0048639	positive regulation of developmental growth	-2.58106
GO:0034763	negative regulation of transmembrane transport	-2.57669
GO:0051147	regulation of muscle cell differentiation	-2.56958
GO:0010661	positive regulation of muscle cell apoptotic process	-2.56815
GO:0032094	response to food	-2.56815
GO:0032689	negative regulation of interferon-gamma production	-2.56815
GO:0042769	DNA damage response, detection of DNA damage	-2.56815
GO:0045923	positive regulation of fatty acid metabolic process	-2.56815
GO:0051954	positive regulation of amine transport	-2.56815
GO:0051955	regulation of amino acid transport	-2.56815
GO:0060045	positive regulation of cardiac muscle cell proliferation	-2.56815
GO:0071604	transforming growth factor beta production	-2.56815

GO:0002200	somatic diversification of immune receptors	-2.56326
GO:0030104	water homeostasis	-2.56326
GO:0050672	negative regulation of lymphocyte proliferation	-2.56326
GO:1903556	negative regulation of tumor necrosis factor superfamily cytokine production	-2.56326
GO:0006470	protein dephosphorylation	-2.56292
GO:0008544	epidermis development	-2.54487
GO:0032945	negative regulation of mononuclear cell proliferation	-2.54344
GO:0055021	regulation of cardiac muscle tissue growth	-2.54344
GO:0006656	phosphatidylcholine biosynthetic process	-2.53572
GO:0006778	porphyrin-containing compound metabolic process	-2.53572
GO:0033683	nucleotide-excision repair, DNA incision	-2.53572
GO:0038179	neurotrophin signaling pathway	-2.53572
GO:0045022	early endosome to late endosome transport	-2.53572
GO:0055090	acylglycerol homeostasis	-2.53572
GO:0070328	triglyceride homeostasis	-2.53572
GO:0071867	response to monoamine	-2.53572
GO:0071869	response to catecholamine	-2.53572
GO:1902229	regulation of intrinsic apoptotic signaling pathway in response to DNA damage	-2.53572
GO:2000249	regulation of actin cytoskeleton reorganization	-2.53572
GO:2000816	negative regulation of mitotic sister chromatid separation	-2.53572
GO:0032434	regulation of proteasomal ubiquitin-dependent protein catabolic process	-2.53362
GO:0043010	camera-type eye development	-2.53037

GO:0051279	regulation of release of sequestered calcium ion into cytosol	-2.52391
GO:0042177	negative regulation of protein catabolic process	-2.51952
GO:0090101	negative regulation of transmembrane receptor protein serine/threonine kinase signaling pathway	-2.51952
GO:0061136	regulation of proteasomal protein catabolic process	-2.51326
GO:0045995	regulation of embryonic development	-2.50555
GO:0016126	sterol biosynthetic process	-2.50465
GO:0008207	C21-steroid hormone metabolic process	-2.50418
GO:0010613	positive regulation of cardiac muscle hypertrophy	-2.50418
GO:1905819	negative regulation of chromosome separation	-2.50418
GO:0007031	peroxisome organization	-2.48565
GO:0006997	nucleus organization	-2.47797
GO:0051225	spindle assembly	-2.47797
GO:0007212	dopamine receptor signaling pathway	-2.47351
GO:0009112	nucleobase metabolic process	-2.47351
GO:0014742	positive regulation of muscle hypertrophy	-2.47351
GO:0033574	response to testosterone	-2.47351
GO:0046825	regulation of protein export from nucleus	-2.47351
GO:0050832	defense response to fungus	-2.47351
GO:2000008	regulation of protein localization to cell surface	-2.47351
GO:0051216	cartilage development	-2.4695
GO:0015844	monoamine transport	-2.46692
GO:00330	regulation of sister chromatid segregation	-2.46692

45		
GO:0051651	maintenance of location in cell	-2.46692
GO:0055072	iron ion homeostasis	-2.46692
GO:0030534	adult behavior	-2.46436
GO:0045844	positive regulation of striated muscle tissue development	-2.44844
GO:0048636	positive regulation of muscle organ development	-2.44844
GO:0110110	positive regulation of animal organ morphogenesis	-2.44844
GO:0051590	positive regulation of neurotransmitter transport	-2.44365
GO:0072595	maintenance of protein localization in organelle	-2.44365
GO:0098927	vesicle-mediated transport between endosomal compartments	-2.44365
GO:0006112	energy reserve metabolic process	-2.43022
GO:1901863	positive regulation of muscle tissue development	-2.43022
GO:0031032	actomyosin structure organization	-2.42684
GO:0097553	calcium ion transmembrane import into cytosol	-2.42425
GO:0006308	DNA catabolic process	-2.41457
GO:0046006	regulation of activated T cell proliferation	-2.41457
GO:0050850	positive regulation of calcium-mediated signaling	-2.41457
GO:0050856	regulation of T cell receptor signaling pathway	-2.41457
GO:0071470	cellular response to osmotic stress	-2.41457
GO:0001942	hair follicle development	-2.41223
GO:0030512	negative regulation of transforming growth factor beta receptor signaling pathway	-2.41223
GO:0034109	homotypic cell-cell adhesion	-2.41223

GO:0060420	regulation of heart growth	-2.41223
GO:0007043	cell-cell junction assembly	-2.41111
GO:1903322	positive regulation of protein modification by small protein conjugation or removal	-2.41111
GO:0045017	glycerolipid biosynthetic process	-2.41069
GO:0007051	spindle organization	-2.39554
GO:0002028	regulation of sodium ion transport	-2.39448
GO:0033173	calcineurin-NFAT signaling cascade	-2.38623
GO:0042088	T-helper 1 type immune response	-2.38623
GO:0042181	ketone biosynthetic process	-2.38623
GO:0022404	molting cycle process	-2.37697
GO:0022405	hair cycle process	-2.37697
GO:1903845	negative regulation of cellular response to transforming growth factor beta stimulus	-2.37697
GO:0007265	Ras protein signal transduction	-2.37532
GO:0050852	T cell receptor signaling pathway	-2.37499
GO:0060041	retina development in camera-type eye	-2.37235
GO:0043588	skin development	-2.36817
GO:0098773	skin epidermis development	-2.35968
GO:0010677	negative regulation of cellular carbohydrate metabolic process	-2.35859
GO:0021795	cerebral cortex cell migration	-2.35859
GO:0031018	endocrine pancreas development	-2.35859
GO:0042771	intrinsic apoptotic signaling pathway in response to DNA damage by p53 class mediator	-2.35859
GO:00903	regulation of protein deacetylation	-2.35859

11		
GO:0140115	export across plasma membrane	-2.35859
GO:0044344	cellular response to fibroblast growth factor stimulus	-2.34705
GO:0048813	dendrite morphogenesis	-2.34705
GO:0045921	positive regulation of exocytosis	-2.34261
GO:0051492	regulation of stress fiber assembly	-2.34261
GO:2000177	regulation of neural precursor cell proliferation	-2.34261
GO:0042742	defense response to bacterium	-2.33844
GO:0098742	cell-cell adhesion via plasma-membrane adhesion molecules	-2.33362
GO:0014014	negative regulation of gliogenesis	-2.33162
GO:0097028	dendritic cell differentiation	-2.33162
GO:1903321	negative regulation of protein modification by small protein conjugation or removal	-2.32576
GO:0034332	adherens junction organization	-2.30988
GO:0008542	visual learning	-2.3053
GO:0033003	regulation of mast cell activation	-2.3053
GO:0035850	epithelial cell differentiation involved in kidney development	-2.3053
GO:0046638	positive regulation of alpha-beta T cell differentiation	-2.3053
GO:1904738	vascular associated smooth muscle cell migration	-2.3053
GO:1904752	regulation of vascular associated smooth muscle cell migration	-2.3053
GO:0001501	skeletal system development	-2.30036
GO:0035264	multicellular organism growth	-2.29769
GO:0030641	regulation of cellular pH	-2.29268

GO:0045778	positive regulation of ossification	-2.29268
GO:0051304	chromosome separation	-2.29268
GO:0016331	morphogenesis of embryonic epithelium	-2.2856
GO:0002820	negative regulation of adaptive immune response	-2.27959
GO:0031952	regulation of protein autophosphorylation	-2.27959
GO:0033628	regulation of cell adhesion mediated by integrin	-2.27959
GO:0043370	regulation of CD4-positive, alpha-beta T cell differentiation	-2.27959
GO:0050798	activated T cell proliferation	-2.27959
GO:0051932	synaptic transmission, GABAergic	-2.27959
GO:0055023	positive regulation of cardiac muscle tissue growth	-2.27959
GO:0060324	face development	-2.27959
GO:0061001	regulation of dendritic spine morphogenesis	-2.27959
GO:1900744	regulation of p38MAPK cascade	-2.27959
GO:0048814	regulation of dendrite morphogenesis	-2.27645
GO:0070167	regulation of biomineral tissue development	-2.27645
GO:0099003	vesicle-mediated transport in synapse	-2.2759
GO:0007605	sensory perception of sound	-2.27361
GO:0016202	regulation of striated muscle tissue development	-2.26171
GO:0031056	regulation of histone modification	-2.26171
GO:0071774	response to fibroblast growth factor	-2.26171
GO:0031279	regulation of cyclase activity	-2.25447
GO:0042059	negative regulation of epidermal growth factor receptor signaling pathway	-2.25447

GO:0043277	apoptotic cell clearance	-2.25447
GO:0070231	T cell apoptotic process	-2.25447
GO:0097720	calcineurin-mediated signaling	-2.25447
GO:0017156	calcium ion regulated exocytosis	-2.24991
GO:0006605	protein targeting	-2.24671
GO:0030516	regulation of axon extension	-2.24458
GO:0035710	CD4-positive, alpha-beta T cell activation	-2.24458
GO:0120162	positive regulation of cold-induced thermogenesis	-2.24458
GO:0010976	positive regulation of neuron projection development	-2.24376
GO:0060485	mesenchyme development	-2.24376
GO:0030509	BMP signaling pathway	-2.2382
GO:0050773	regulation of dendrite development	-2.2382
GO:1902115	regulation of organelle assembly	-2.23789
GO:0008038	neuron recognition	-2.22992
GO:0031648	protein destabilization	-2.22992
GO:0043300	regulation of leukocyte degranulation	-2.22992
GO:0043303	mast cell degranulation	-2.22992
GO:0048546	digestive tract morphogenesis	-2.22992
GO:0010771	negative regulation of cell morphogenesis involved in differentiation	-2.22893
GO:0008277	regulation of G protein-coupled receptor signaling pathway	-2.22659
GO:1901861	regulation of muscle tissue development	-2.22659
GO:0007163	establishment or maintenance of cell polarity	-2.21922

GO:0048489	synaptic vesicle transport	-2.21506
GO:0048634	regulation of muscle organ development	-2.21506
GO:0097480	establishment of synaptic vesicle localization	-2.21506
GO:0002204	somatic recombination of immunoglobulin genes involved in immune response	-2.20591
GO:0002208	somatic diversification of immunoglobulins involved in immune response	-2.20591
GO:0002279	mast cell activation involved in immune response	-2.20591
GO:0045190	isotype switching	-2.20591
GO:0006790	sulfur compound metabolic process	-2.2046
GO:0007519	skeletal muscle tissue development	-2.20363
GO:0030203	glycosaminoglycan metabolic process	-2.20363
GO:2000241	regulation of reproductive process	-2.20363
GO:0043583	ear development	-2.20076
GO:2001020	regulation of response to DNA damage stimulus	-2.18251
GO:0001912	positive regulation of leukocyte mediated cytotoxicity	-2.18242
GO:0002448	mast cell mediated immunity	-2.18242
GO:0002704	negative regulation of leukocyte mediated immunity	-2.18242
GO:0046580	negative regulation of Ras protein signal transduction	-2.18242
GO:0060421	positive regulation of heart growth	-2.18242
GO:0060425	lung morphogenesis	-2.18242
GO:0044264	cellular polysaccharide metabolic process	-2.16816
GO:0060191	regulation of lipase activity	-2.16816
GO:0110020	regulation of actomyosin structure organization	-2.16816

GO:0007632	visual behavior	-2.15943
GO:0032964	collagen biosynthetic process	-2.15943
GO:0038066	p38MAPK cascade	-2.15943
GO:2000772	regulation of cellular senescence	-2.15943
GO:0019827	stem cell population maintenance	-2.15877
GO:0002042	cell migration involved in sprouting angiogenesis	-2.1534
GO:0006885	regulation of pH	-2.1534
GO:0006959	humoral immune response	-2.15105
GO:0002673	regulation of acute inflammatory response	-2.14776
GO:0051494	negative regulation of cytoskeleton organization	-2.14776
GO:0046474	glycerophospholipid biosynthetic process	-2.14663
GO:0048762	mesenchymal cell differentiation	-2.14663
GO:0003300	cardiac muscle hypertrophy	-2.13881
GO:0032231	regulation of actin filament bundle assembly	-2.13881
GO:0006584	catecholamine metabolic process	-2.13692
GO:0006968	cellular defense response	-2.13692
GO:0009712	catechol-containing compound metabolic process	-2.13692
GO:0044764	multi-organism cellular process	-2.13692
GO:1901185	negative regulation of ERBB signaling pathway	-2.13692
GO:0000070	mitotic sister chromatid segregation	-2.13685
GO:0098727	maintenance of cell number	-2.13685
GO:0048738	cardiac muscle tissue development	-2.12899

GO:0002824	positive regulation of adaptive immune response based on somatic recombination of immune receptors built from immunoglobulin superfamily domains	-2.12439
GO:1902106	negative regulation of leukocyte differentiation	-2.12439
GO:0001954	positive regulation of cell-matrix adhesion	-2.11488
GO:0042304	regulation of fatty acid biosynthetic process	-2.11488
GO:0002708	positive regulation of lymphocyte mediated immunity	-2.11013
GO:0007631	feeding behavior	-2.11013
GO:0019395	fatty acid oxidation	-2.11013
GO:0014897	striated muscle hypertrophy	-2.09603
GO:0055017	cardiac muscle tissue growth	-2.09603
GO:0099175	regulation of postsynapse organization	-2.09603
GO:0060538	skeletal muscle organ development	-2.09398
GO:0071772	response to BMP	-2.09398
GO:0071773	cellular response to BMP stimulus	-2.09398
GO:0002712	regulation of B cell mediated immunity	-2.09328
GO:0002889	regulation of immunoglobulin mediated immune response	-2.09328
GO:0030071	regulation of mitotic metaphase/anaphase transition	-2.09328
GO:0043113	receptor clustering	-2.09328
GO:0060688	regulation of morphogenesis of a branching structure	-2.09328
GO:0071825	protein-lipid complex subunit organization	-2.09328
GO:0090183	regulation of kidney development	-2.09328
GO:1903307	positive regulation of regulated secretory pathway	-2.09328

GO:0048588	developmental cell growth	-2.08574
GO:0006024	glycosaminoglycan biosynthetic process	-2.08208
GO:0061005	cell differentiation involved in kidney development	-2.07211
GO:0014896	muscle hypertrophy	-2.06828
GO:0051224	negative regulation of protein transport	-2.06266
GO:0090305	nucleic acid phosphodiester bond hydrolysis	-2.06266
GO:0031346	positive regulation of cell projection organization	-2.05481
GO:0002821	positive regulation of adaptive immune response	-2.05464
GO:0006641	triglyceride metabolic process	-2.05464
GO:0006022	aminoglycan metabolic process	-2.05238
GO:1990138	neuron projection extension	-2.05238
GO:0034249	negative regulation of cellular amide metabolic process	-2.052
GO:0007091	metaphase/anaphase transition of mitotic cell cycle	-2.05136
GO:0010656	negative regulation of muscle cell apoptotic process	-2.05136
GO:0051055	negative regulation of lipid biosynthetic process	-2.05136
GO:1902099	regulation of metaphase/anaphase transition of cell cycle	-2.05136
GO:0051149	positive regulation of muscle cell differentiation	-2.04114
GO:0061387	regulation of extent of cell growth	-2.04114
GO:0043030	regulation of macrophage activation	-2.03101
GO:0051058	negative regulation of small GTPase mediated signal transduction	-2.03101
GO:0043087	regulation of GTPase activity	-2.03038
GO:0006941	striated muscle contraction	-2.02196

GO:0010921	regulation of phosphatase activity	-2.02196
GO:1904950	negative regulation of establishment of protein localization	-2.02196
GO:0002223	stimulatory C-type lectin receptor signaling pathway	-2.01458
GO:0030004	cellular monovalent inorganic cation homeostasis	-2.01458
GO:0042093	T-helper cell differentiation	-2.01104
GO:0044784	metaphase/anaphase transition of cell cycle	-2.01104

Cellular component

GO	Description	LogP
GO:1902911	protein kinase complex	-13.4395
GO:1902554	serine/threonine protein kinase complex	-11.6033
GO:0061695	transferase complex, transferring phosphorus-containing groups	-11.1846
GO:0098552	side of membrane	-10.3754
GO:0045121	membrane raft	-10.2864
GO:0098857	membrane microdomain	-10.2649
GO:0098589	membrane region	-9.9929
GO:0031983	vesicle lumen	-9.18179
GO:0035976	transcription factor AP-1 complex	-8.55394
GO:0060205	cytoplasmic vesicle lumen	-8.31367
GO:0009897	external side of plasma membrane	-7.85534
GO:0034774	secretory granule lumen	-7.75074
GO:0000307	cyclin-dependent protein kinase holoenzyme complex	-7.58061

GO:0031093	platelet alpha granule lumen	-7.48831
GO:0005667	transcription factor complex	-7.33899
GO:0044853	plasma membrane raft	-6.77073
GO:0005901	caveola	-6.75021
GO:0031012	extracellular matrix	-6.62528
GO:0031091	platelet alpha granule	-6.43856
GO:0048471	perinuclear region of cytoplasm	-6.33181
GO:0043025	neuronal cell body	-5.76736
GO:0005635	nuclear envelope	-5.61878
GO:0005788	endoplasmic reticulum lumen	-5.4972
GO:0062023	collagen-containing extracellular matrix	-5.43312
GO:1990234	transferase complex	-5.35132
GO:0043235	receptor complex	-5.1432
GO:0044297	cell body	-5.07546
GO:0005911	cell-cell junction	-5.06478
GO:0098984	neuron to neuron synapse	-4.71992
GO:0005815	microtubule organizing center	-4.68465
GO:0098794	postsynapse	-4.65222
GO:0000323	lytic vacuole	-4.65007
GO:0005764	lysosome	-4.65007
GO:0090575	RNA polymerase II transcription factor complex	-4.64391
GO:0030425	dendrite	-4.63587

GO:0030424	axon	-4.62772
GO:0097447	dendritic tree	-4.61959
GO:0019897	extrinsic component of plasma membrane	-4.38032
GO:0005770	late endosome	-4.3768
GO:0014069	postsynaptic density	-4.37511
GO:0044798	nuclear transcription factor complex	-4.34472
GO:0032279	asymmetric synapse	-4.30604
GO:0005912	adherens junction	-4.19225
GO:0005902	microvillus	-4.19157
GO:0005925	focal adhesion	-4.1845
GO:0005924	cell-substrate adherens junction	-4.15501
GO:0099572	postsynaptic specialization	-4.10765
GO:0030055	cell-substrate junction	-4.10646
GO:0070161	anchoring junction	-4.06144
GO:0005819	spindle	-4.04429
GO:0016323	basolateral plasma membrane	-4.02146
GO:0045177	apical part of cell	-4.02091
GO:0005813	centrosome	-3.95878
GO:0015629	actin cytoskeleton	-3.94216
GO:0005876	spindle microtubule	-3.90409
GO:0032993	protein-DNA complex	-3.84788
GO:0009898	cytoplasmic side of plasma membrane	-3.66766

GO:0031968	organelle outer membrane	-3.66566
GO:0019867	outer membrane	-3.6388
GO:0098858	actin-based cell projection	-3.6388
GO:0030659	cytoplasmic vesicle membrane	-3.63512
GO:0031965	nuclear membrane	-3.39701
GO:0030496	midbody	-3.39334
GO:0019898	extrinsic component of membrane	-3.34531
GO:0098562	cytoplasmic side of membrane	-3.30905
GO:0030139	endocytic vesicle	-3.27471
GO:0005741	mitochondrial outer membrane	-3.25464
GO:0031234	extrinsic component of cytoplasmic side of plasma membrane	-3.08543
GO:0033267	axon part	-3.05903
GO:0043204	perikaryon	-2.97965
GO:0031252	cell leading edge	-2.93288
GO:0098687	chromosomal region	-2.88179
GO:0030666	endocytic vesicle membrane	-2.86813
GO:0000780	condensed nuclear chromosome, centromeric region	-2.78481
GO:0005740	mitochondrial envelope	-2.76785
GO:0030135	coated vesicle	-2.76099
GO:0098793	presynapse	-2.74701
GO:0098797	plasma membrane protein complex	-2.71795
GO:0097386	glial cell projection	-2.70797

GO:0005923	bicellular tight junction	-2.682
GO:1904813	ficolin-1-rich granule lumen	-2.6512
GO:0051233	spindle midzone	-2.63593
GO:0031594	neuromuscular junction	-2.62447
GO:0048786	presynaptic active zone	-2.62447
GO:0101002	ficolin-1-rich granule	-2.60426
GO:0070160	tight junction	-2.57669
GO:0001772	immunological synapse	-2.53572
GO:0030136	clathrin-coated vesicle	-2.48034
GO:0098685	Schaffer collateral - CA1 synapse	-2.46692
GO:0043296	apical junction complex	-2.38516
GO:0031970	organelle envelope lumen	-2.35968
GO:0035578	azurophil granule lumen	-2.34261
GO:0098978	glutamatergic synapse	-2.27383
GO:0031253	cell projection membrane	-2.26679
GO:0016324	apical plasma membrane	-2.25977
GO:0009925	basal plasma membrane	-2.20591
GO:0000793	condensed chromosome	-2.20076
GO:0000794	condensed nuclear chromosome	-2.1534
GO:0005796	Golgi lumen	-2.1534
GO:0000781	chromosome, telomeric region	-2.14776
GO:0000922	spindle pole	-2.12601

GO:0031966	mitochondrial membrane	-2.11924
GO:0030863	cortical cytoskeleton	-2.11013
GO:0150034	distal axon	-2.09881
GO:0005775	vacuolar lumen	-2.04216
GO:0005637	nuclear inner membrane	-2.01104

Molecular function

ID	Description	LogP
GO:0016773	phosphotransferase activity, alcohol group as acceptor	-30.1752
GO:0004672	protein kinase activity	-29.9415
GO:0016301	kinase activity	-28.4354
GO:0019900	kinase binding	-19.4127
GO:0004674	protein serine/threonine kinase activity	-18.6932
GO:0019901	protein kinase binding	-17.3769
GO:0005126	cytokine receptor binding	-17.212
GO:0008134	transcription factor binding	-16.0449
GO:0030545	receptor regulator activity	-14.474
GO:00199	protein domain specific binding	-

04		13.9246
GO:0016491	oxidoreductase activity	-13.2629
GO:0050839	cell adhesion molecule binding	-13.0033
GO:0020037	heme binding	-12.9582
GO:0070851	growth factor receptor binding	-12.8189
GO:0005125	cytokine activity	-12.7838
GO:0048018	receptor ligand activity	-12.6537
GO:0046906	tetrapyrrole binding	-12.5063
GO:0031072	heat shock protein binding	-12.2138
GO:0019902	phosphatase binding	-11.9094
GO:0005178	integrin binding	-11.4578
GO:0042803	protein homodimerization activity	-11.4034
GO:0004713	protein tyrosine kinase activity	-10.4628
GO:0001228	DNA-binding transcription activator activity, RNA polymerase II-specific	-10.4042
GO:0048037	cofactor binding	-9.68905

GO:0004497	monooxygenase activity	-9.60715
GO:0016705	oxidoreductase activity, acting on paired donors, with incorporation or reduction of molecular oxygen	-9.59211
GO:0070330	aromatase activity	-9.35846
GO:0051879	Hsp90 protein binding	-9.16273
GO:0019903	protein phosphatase binding	-8.94469
GO:0035173	histone kinase activity	-8.78031
GO:0008083	growth factor activity	-8.52853
GO:0016538	cyclin-dependent protein serine/threonine kinase regulator activity	-8.52661
GO:0004879	nuclear receptor activity	-8.3854
GO:0098531	transcription factor activity, direct ligand regulated sequence-specific DNA binding	-8.3854
GO:0016712	oxidoreductase activity, acting on paired donors, with incorporation or reduction of molecular oxygen, reduced flavin or flavoprotein as one donor, and incorporation of one atom of oxygen	-8.2396
GO:0019825	oxygen binding	-8.14838
GO:0008395	steroid hydroxylase activity	-7.97427
GO:0016209	antioxidant activity	-7.84315
GO:00055	iron ion binding	-

06		7.787 78
GO:0004714	transmembrane receptor protein tyrosine kinase activity	-7.648 53
GO:0008559	xenobiotic transmembrane transporting ATPase activity	-7.382 85
GO:0051087	chaperone binding	-7.116 12
GO:0001221	transcription cofactor binding	-7.046 69
GO:0044389	ubiquitin-like protein ligase binding	-7.000 38
GO:0019199	transmembrane receptor protein kinase activity	-6.750 21
GO:0045296	cadherin binding	-6.722 41
GO:0101020	estrogen 16-alpha-hydroxylase activity	-6.718 84
GO:0051219	phosphoprotein binding	-6.669 25
GO:0031625	ubiquitin protein ligase binding	-6.478 8
GO:0016725	oxidoreductase activity, acting on CH or CH2 groups	-6.246 72
GO:0042562	hormone binding	-6.122 61
GO:0004712	protein serine/threonine/tyrosine kinase activity	-6.036 15
GO:0016709	oxidoreductase activity, acting on paired donors, with incorporation or reduction of molecular oxygen, NAD(P)H as one donor, and incorporation of one atom of oxygen	-6.036 15

GO:0005496	steroid binding	-6.02433
GO:0035257	nuclear hormone receptor binding	-5.90973
GO:0001085	RNA polymerase II transcription factor binding	-5.88463
GO:0033695	oxidoreductase activity, acting on CH or CH2 groups, quinone or similar compound as acceptor	-5.81257
GO:0034875	caffeine oxidase activity	-5.81257
GO:0097200	cysteine-type endopeptidase activity involved in execution phase of apoptosis	-5.72213
GO:0001223	transcription coactivator binding	-5.68524
GO:0035258	steroid hormone receptor binding	-5.64964
GO:0097153	cysteine-type endopeptidase activity involved in apoptotic process	-5.4463
GO:0004089	carbonate dehydratase activity	-5.32385
GO:0019207	kinase regulator activity	-5.32343
GO:0004175	endopeptidase activity	-5.25097
GO:0051427	hormone receptor binding	-5.17235
GO:0043559	insulin binding	-5.11831
GO:0002039	p53 binding	-5.044

		16
GO:0003682	chromatin binding	-5.03919
GO:0019887	protein kinase regulator activity	-5.01744
GO:0005518	collagen binding	-4.89542
GO:0030235	nitric-oxide synthase regulator activity	-4.87762
GO:0001784	phosphotyrosine residue binding	-4.86371
GO:0030544	Hsp70 protein binding	-4.86371
GO:0046982	protein heterodimerization activity	-4.56707
GO:0016702	oxidoreductase activity, acting on single donors with incorporation of molecular oxygen, incorporation of two atoms of oxygen	-4.50428
GO:0042910	xenobiotic transmembrane transporter activity	-4.50428
GO:0003707	steroid hormone receptor activity	-4.43422
GO:0016701	oxidoreductase activity, acting on single donors with incorporation of molecular oxygen	-4.43422
GO:0008013	beta-catenin binding	-4.383
GO:0045309	protein phosphorylated amino acid binding	-4.36399
GO:0043560	insulin receptor substrate binding	-4.34957
GO:0097199	cysteine-type endopeptidase activity involved in apoptotic signaling pathway	-4.349

		57
GO:0050661	NADP binding	-4.32437
GO:0070011	peptidase activity, acting on L-amino acid peptides	-4.26439
GO:0016684	oxidoreductase activity, acting on peroxide as acceptor	-4.24751
GO:0004693	cyclin-dependent protein serine/threonine kinase activity	-4.24065
GO:0097472	cyclin-dependent protein kinase activity	-4.24065
GO:0030546	receptor activator activity	-4.21362
GO:0030332	cyclin binding	-4.18099
GO:0043548	phosphatidylinositol 3-kinase binding	-4.18099
GO:0002020	protease binding	-4.15379
GO:0019838	growth factor binding	-4.15379
GO:0016836	hydro-lyase activity	-4.13775
GO:0050662	coenzyme binding	-4.12502
GO:0008233	peptidase activity	-4.12489
GO:0004861	cyclin-dependent protein serine/threonine kinase inhibitor activity	-4.09103
GO:00083	RNA polymerase II CTD heptapeptide repeat kinase activity	-

53		4.091 03
GO:0015562	efflux transmembrane transporter activity	-3.87705
GO:0042805	actinin binding	-3.86359
GO:0047485	protein N-terminus binding	-3.85345
GO:0016651	oxidoreductase activity, acting on NAD(P)H	-3.80929
GO:0035259	glucocorticoid receptor binding	-3.78249
GO:0009055	electron transfer activity	-3.76605
GO:0004697	protein kinase C activity	-3.69466
GO:0004698	calcium-dependent protein kinase C activity	-3.69466
GO:0016835	carbon-oxygen lyase activity	-3.64137
GO:0001618	virus receptor activity	-3.61438
GO:0104005	hijacked molecular function	-3.61438
GO:0008201	heparin binding	-3.58799
GO:0004708	MAP kinase kinase activity	-3.53585
GO:0050660	flavin adenine dinucleotide binding	-3.48518

GO:0001091	RNA polymerase II basal transcription factor binding	-3.46356
GO:0004715	non-membrane spanning protein tyrosine kinase activity	-3.44588
GO:0004707	MAP kinase activity	-3.39532
GO:0032813	tumor necrosis factor receptor superfamily binding	-3.37442
GO:0033218	amide binding	-3.25256
GO:0016829	lyase activity	-3.24126
GO:0017171	serine hydrolase activity	-3.24126
GO:0004601	peroxidase activity	-3.2409
GO:0042277	peptide binding	-3.22551
GO:0005158	insulin receptor binding	-3.21104
GO:0009931	calcium-dependent protein serine/threonine kinase activity	-3.21104
GO:0070412	R-SMAD binding	-3.21104
GO:0010857	calcium-dependent protein kinase activity	-3.15539
GO:0070696	transmembrane receptor protein serine/threonine kinase binding	-3.15539
GO:0005516	calmodulin binding	-3.149

		85
GO:0005080	protein kinase C binding	-3.14803
GO:0008289	lipid binding	-3.12875
GO:0004190	aspartic-type endopeptidase activity	-3.10221
GO:0035586	purinergic receptor activity	-3.10221
GO:0070001	aspartic-type peptidase activity	-3.0513
GO:0005507	copper ion binding	-3.00512
GO:0097110	scaffold protein binding	-3.00512
GO:0046875	ephrin receptor binding	-3.00249
GO:0051393	alpha-actinin binding	-3.00249
GO:0004222	metalloendopeptidase activity	-2.91572
GO:0005520	insulin-like growth factor binding	-2.91052
GO:0033612	receptor serine/threonine kinase binding	-2.91052
GO:0031406	carboxylic acid binding	-2.86399
GO:0005164	tumor necrosis factor receptor binding	-2.82523
GO:00302	protein serine/threonine kinase inhibitor activity	-

91		2.825 23
GO:00510 59	NF-kappaB binding	- 2.825 23
GO:00428 26	histone deacetylase binding	- 2.794 7
GO:00042 52	serine-type endopeptidase activity	- 2.775 81
GO:00055 39	glycosaminoglycan binding	- 2.755 92
GO:00423 79	chemokine receptor binding	- 2.755 58
GO:00431 77	organic acid binding	-2.735
GO:00010 98	basal transcription machinery binding	- 2.710 51
GO:00010 99	basal RNA polymerase II transcription machinery binding	- 2.710 51
GO:00080 47	enzyme activator activity	- 2.708 59
GO:00051 54	epidermal growth factor receptor binding	- 2.707 97
GO:00463 32	SMAD binding	- 2.563 26
GO:00082 36	serine-type peptidase activity	- 2.558 17
GO:00336 13	activating transcription factor binding	- 2.543 44
GO:00015 40	amyloid-beta binding	- 2.523 91
GO:00421	SH2 domain binding	-

69		2.473 51
GO:19016 81	sulfur compound binding	- 2.463 97
GO:00303 31	estrogen receptor binding	- 2.443 65
GO:00430 28	cysteine-type endopeptidase regulator activity involved in apoptotic process	- 2.414 57
GO:00511 17	ATPase binding	- 2.394 48
GO:00481 56	tau protein binding	- 2.358 59
GO:00512 13	dioxygenase activity	- 2.342 61
GO:00010 46	core promoter sequence-specific DNA binding	- 2.305 3
GO:00010 47	core promoter binding	- 2.305 3
GO:00011 02	RNA polymerase II activating transcription factor binding	- 2.305 3
GO:00080 09	chemokine activity	- 2.254 47
GO:00048 96	cytokine receptor activity	- 2.244 58
GO:00016 64	G protein-coupled receptor binding	- 2.220 04
GO:00170 46	peptide hormone binding	- 2.205 91
GO:00436 21	protein self-association	- 2.072 11

GO:0015238	drug transmembrane transporter activity	-2.01458
GO:0016597	amino acid binding	-2.01104

Table S5. 49 signaling pathways in KEGG pathway enrichment

ID	Description	LogP
hsa05200	Pathways in cancer	-49.811
hsa04668	TNF signaling pathway	-34.1958
hsa04657	IL-17 signaling pathway	-33.2423
hsa04933	AGE-RAGE signaling pathway in diabetic complications	-30.0409
hsa04068	foxo signaling pathway	-25.599
hsa04151	PI3K-Akt signaling pathway	-24.9743
hsa04010	MAPK signaling pathway	-21.2708
hsa04660	T cell receptor signaling pathway	-19.8973
hsa04066	HIF-1 signaling pathway	-19.5263
hsa04064	NF-kappa B signaling pathway	-18.9481
hsa04625	c-type lectin receptor signaling pathway	-18.9481
hsa04620	Toll-like receptor signaling pathway	-18.6471
hsa04012	ErbB signaling pathway	-18.484
hsa04926	relaxin signaling pathway	-18.3847
hsa04115	p53 signaling pathway	-16.7018
hsa04664	Fc epsilon RI signaling pathway	-16.6144
hsa04062	Chemokine signaling pathway	-16.3209
hsa04621	NOD-like receptor signaling pathway	-15.7797
hsa04917	Prolactin signaling pathway	-15.5108
hsa04014	Ras signaling pathway	-15.3423
hsa04015	Rap1 signaling pathway	-14.1374
hsa04630	JAK-STAT signaling pathway	-13.9678
hsa04915	Estrogen signaling pathway	-13.8456
hsa04662	B cell receptor signaling pathway	-12.107
hsa04370	VEGF signaling pathway	-11.8037
hsa04722	Neurotrophin signaling pathway	-11.0973
hsa04912	GnRH signaling pathway	-11.094
hsa04211	Longevity regulating pathway	-10.5382
hsa04024	cAMP signaling pathway	-9.85272
hsa04071	Sphingolipid signaling pathway	-9.71165
hsa04910	Insulin signaling pathway	-8.74773

hsa04919	thyroid hormone signaling pathway	-8.72817
hsa04622	RIG-I-like receptor signaling pathway	-8.53742
hsa04152	AMPK signaling pathway	-8.50766
hsa04072	Phospholipase D signaling pathway	-8.49809
hsa04213	Longevity regulating pathway - multiple species	-7.76007
hsa04623	Cytosolic DNA-sensing pathway	-7.64853
hsa04150	mTOR signaling pathway	-7.58329
hsa04921	Oxytocin signaling pathway	-7.49878
hsa04920	Adipocytokine signaling pathway	-5.99848
hsa04371	Apelin signaling pathway	-4.89243
hsa04550	Signaling pathways regulating pluripotency of stem cells	-4.74471
hsa04310	Wnt signaling pathway	-3.58799
hsa04350	TGF-beta signaling pathway	-3.20877
hsa04020	Calcium signaling pathway	-3.07456
hsa04922	Glucagon signaling pathway	-2.93376
hsa04022	cGMP-PKG signaling pathway	-2.75027
hsa04390	Hippo signaling pathway	-2.73763
hsa04340	Hedgehog signaling pathway	-2.25447
